# Rice straw structure changes following green pretreatment with petha wastewater for economically viable bioethanol production

**DOI:** 10.1038/s41598-022-14627-7

**Published:** 2022-06-21

**Authors:** Dolly Kumari, Radhika Singh

**Affiliations:** Biohydrogen Production Lab, Department of Chemistry, Faculty of Science, Dayalbagh Educational Institute, Dayalbagh, Agra, 282005 India

**Keywords:** Environmental sciences, Chemistry

## Abstract

Energy efficient and environment friendly pretreatment processes for the production of biofuel have remained elusive and the research is further compounded by the high cost of processing lignocellulosic biomass—an essential factor for producing sustainable biofuels. In the last few decades, a number of pretreatment methods have been proposed, specifically chemical pretreatments but are either expensive or harmful to the environment. To address this urgent need, we propose a green pretreatment method that utilises the highly alkaline by-product, petha wastewater to pretreat the lignocellulosic waste rice straw (RS). The effectiveness of the pretreatment was analysed by monitoring both enhanced cellulose content and reducing sugar yield along with removal of hemicellulose and lignin. We found that PWW pretreatment yielded five times more reducing sugar than native RS with 10.12% increment in cellulose content. SEM and EDX studies further revealed that our process enhanced surface roughness and carbon content (from 32.19% increased to 41.59% and 41.66% for A and D, respectively) along with reduction in silica content (from 8.68% in RS to 4.30% and 7.72% for A and D, respectively). XRD and FTIR analyses indicate crystallinity index (CI) and alteration in lignocellulosic structure of RS, respectively. Decrease in CI was about 43.4% in A whereas only 4.5% in D as compared to native RS (CI 54.55%). Thereby we found PWW to be better substitute of an alkali for pretreatment of RS with negligible environmental impacts.

## Introduction

### Importance and meaning of biofuels

Biofuels, the fuels derived from biomass provide solutions for three big worldwide energy problems: energy crisis, environmental pollution, and waste organization^1^. Among various biofuels, bioethanol is considered a popular oxygenated improver of gasoline due its high oxygen content (35%) and a high-research octane number (RON 109) (octane number is a measure of antiknocking property of a fuel). Higher RON would result in greater thermal efficiency in future engines by higher compression ratio. High oxygen content is beneficial in reducing NOx emission and is known to improve the efficiency of engines^2^. About 89% of world’s ethanol production is carried out in USA and Brazil from food crops such as corn and sugarcane juice and known as first-generation biofuel. Use of food crops could lead to food versus energy crisis in the future and hence finding alternative sources for production of ethanol should be a priority. So far, lignocellulosic biomass (rice straw) is considered a good alternative for bioethanol production, also termed as second-generation biofuels^1^. Production of third-generation biofuels takes place from algal feedstock i.e. blue-green algae, water hyacinth etc. Fourth-generation biofuels are produced from solar energy by algae and cyanobacteria. Natural anaerobic digestion of moistened RS releases methane directly to the environment and leads to global warming. The primary current practices utilized for RS disposal (mainly field burning) adds to carbon dioxide content in greenhouse gases^3^.

India, global agricultural powerhouse, generates a wide variety and huge amounts of agronomic residues such as rice straw, wheat straw, oil crop residue, corn cobs, and sugarcane bagasse etc. It is estimated that about 600 MT/year of this yield can be utilised for generation of biofuels^4^. Rice, which is the third major crop grown in India, accounts for about 20% of total rice production all around the world and produces approximately 100 to 110 MT RS every year^5^. Rice straw is chemically composed of cellulose (32–47%), hemicellulose (19–27%), lignin (5–24%), ash (10–17%) and silica content (10–18%)^6^. Although RS has a good amount of cellulose, its high ash and silica content restricts its direct conversion into biofuel, rendering pretreatment a prerequisite for the purpose of generation of biofuel. During the pretreatment process, removal of lignin and hemicellulose resulted in the exposure of cellulose to hydrolytic enzymes and microbial activity which drives biofuel yields. The pretreatment results an increase in surface area, reduction in crystallinity index, and also disintegrate several lignin fractions^3^. Osman et al., in their recently published article, listed various kinds of feedstocks (i.e., agricultural biomass, oil crop residues, algal biomass, blue-green algae etc.) used for production of biofuels in different generations (first, second, third and fourth-generation) with a number of pretreatments used. They have also mentioned that production of third and fourth-generation biofuels will be the future demand of the society and there is an immediate need to devise technologies for fulfilling the increasing future energy demand^7^.

### Literature review

A review of literature reveals that chemical pretreatment methods are not typically environmental friendly whereas, physical pretreatment methods are operationally weak. However, upon combining the two techniques, a higher efficiency has been reported in the pretreatment process. It has been concluded that the combined pretreatment methods are found more efficient as compared to single pretreatment process^3^. A recent review by Osman et al.^8^ mentioned several pretreatment methods and biomass conversions (i.e. biochemical and thermochemical conversion) which elucidated the advantages and drawbacks of certain methods. Thermochemical methods also typically require high energy consumption along with addition of a solvent or a catalyst. Biochemical methods on the other hand have a prolonged cycle and are less efficient in breaking down refractory biomass materials. It has also been reported that the incorporation of thermochemical and biochemical processes could pose extraordinary challenges-the pretreatment process could eventually kill the microorganisms as these processes utilize catalysts or solvents that are used to suppress the production of poison or other inhibitors during the process. Another drawback of this integration is that it is not cost effective^8^. Pretreatment is highly expensive processing technique in bioethanol production and can cost up to 19% of the total expense of cellulosic ethanol production^9^. A variety pretreatment processes—physical (milling, microwave, ultrasonication, freezing etc.), chemical (acid, alkali, ionic liquid, ozonolysis, organosolv etc.), physico-chemical (glycerol thermal, ultrasonication assisted acid and alkali, microwave assisted acid and alkali etc.), biological (fungal and microbial consortium) and combined (combination of two or more pretreatments) are available^3,4,8–15^. Widely used and effective acid and alkali pretreatments are expensive and detrimental to the environment^3^. These challenges call for a better, energy efficient and cheaper solution of biofuel production.

### The gaps of literature

A wide review of literature in the biofuel field reveals that several lab scale methods are available for the pretreatment of lignocellulosic biomass to produce second, third and fourth-generation biofuels. However, very few have focussed on industrialisation of these methods. Higher production costs and low conversion rates of raw biomass are not particularly attractive to biofuel industries, looking to making hefty margins from such processes. According to a recent review, a considerable gap in the literature alarming the latter with a recent research interest concerning techno-economic investigation and life cycle consideration. This can only be attained when the used technology has been sustained to be financially practicable via techno-economic evaluation, and more future research has been accompanied in the emphasised focus areas^7^.

### Objective of the present study

Agra, the city of the Taj Mahal, is well known for its petha sweet, an ash gourd based delicacy. Petha is prepared in more than 500 registered cottage units^16^. These petha cottage units utilise lime for processing of petha sweet from petha fruit. Use of lime (CaO) makes the petha wastewater (about 2000 L per day) highly alkaline (pH = 11–13) due to formation of calcium hydroxide (Ca(OH)_2_) which is typically drained off without any treatment. Our method utilizes this highly alkaline petha wastewater (PWW) for pretreatment of rice straw (RS). As it uses wastewater from petha production, our method is economical and environmentally safe. The highly alkaline nature of PWW is capable of degrading lignin linkage of RS which can then be used for production of second-generation biofuel (methane, ethanol etc.). We used the highly alkaline PWW for the pretreatment of RS and the subsequent change in chemical composition and ultra-structure of native and pretreated RS were compared. To the best of our knowledge, PWW, or in fact any wastewater, has been used first time for the pretreatment of lignocellulosic biomass. This pretreatment method is free of any chemical (normally alkali has been used) and hence can be categorised as green pretreatment of RS. The goal of our study is to develop a chemical free and environmentally benign method to cut the cost of the chemical pretreatment process, utilized for biofuel production. RS is a lignocellulosic waste and PWW is an industrial waste, hence this is a waste to waste pretreatment approach. PWW has not been used earlier for the pretreatment process therefor this is a novel pretreatment method. Disposal of RS has become a global problem whereas PWW disposal is a local problem.

## Materials and methods

### Nomenclature used

RS— Rice straw; A—PWW pretreated RS; D—distilled water pretreated RS.

### Collection and processing of RS and PWW

For the experiment, RS (a cultivated plant waste) which is generally disposed off by burning after harvesting of rice crop was used. Our study complies with relevant institutional, national and international guidelines and legislation. Permissions were obtained from the institute and the farmers for collecting and using the crop waste in this study. RS was pretreated with PWW and its compositional and structural modifications favourable for biofuel production process were evaluated. RS was collected from the nearby farms in Agra. RS was chopped into small pieces (1–2 cm) and was dried under the sun first followed by drying in a hot air oven at 105 °C for about 24 h. The oven dried RS was used for pretreatment with PWW and compositional analyses of RS samples carried out. A blank reference was taken by treating RS with distilled water (neutral pH). Accurately weighed 2.5 g (oven dried) RS was subjected to Soxhlet extraction for removal of extractives (waxes, phenols etc.) with 150 mL acetone at 60 °C for 4 h^17^.

PWW (pH 12.3) was collected from petha industry situated in the narrow streets of Noori Darwaja, Agra. PWW has been earlier used for biohydrogen production by AD process^16^.

### Pretreatment of RS with PWW

For the pretreatment process 5 g RS (5% w/v) was soaked in 100 mL PWW for 7 days at room temperature (35–40 °C). Same amount of RS was also soaked in distilled water for 7 days to compare the effect of PWW on composition of RS. After 7 days RS was removed from PWW and distilled water, both samples were washed and oven dried (105 °C) for 24 h and stored in polybags at room temperature for further use.

### Characterisation of PWW

Characterisation of PWW before and after soaking RS was done for various parameters like total solids (TS), total dissolved solids (TDS), total suspended solids (TSS), volatile solids (VS), ash etc. Biochemical oxygen demand (BOD), chemical oxygen demand (COD), glucose, pH etc. (before and after soaking RS) were also analysed using standard methods^15^.

### Characterisation of native, PWW and distilled water pretreated RS

Native RS, PWW pretreated and distilled water pretreated RS were analysed for several characteristics like cellulose, hemicellulose and lignin using different techniques. Scanning electron microscopy (SEM) and energy dispersive X-ray spectroscopy (EDX) were used for surface and elemental analyses, respectively. Fourier transform infrared (FTIR) was conducted to monitor structural modifications and X-ray diffraction (XRD) was carried out to measure crystallinity index. All analyses were performed in triplicates and average values have been reported. Results were also compared with distilled water pretreated RS.

## Results and discussions

### Change in chemical composition

PWW pretreatment of RS was found effective as indicated by its compositional analysis done using standard methods^18^. Alkali pretreatment solubilise cellulose, hemicellulose, lignin and silica content of lignocellulosic biomass thereby increasing accessibility to cellulose as lignin and acetyl groups are removed^19^. Various % components of native, PWW pretreated (A) and distilled water pretreated RS (D) are given in Table 1 which shows higher increase in cellulose content for A as compared to D. We also found that hemicellulose and lignin content decreased in RS (A). This can be attributed to the alkaline nature of PWW. Hemicellulose is a susceptible component of RS for acid and alkali pretreatment because it solubilises in alkali. Aggarwal et al.^20^ recently reported using organosolv and alkaline pretreatment of RS for cellulose production with about 82% total removal of hemicellulose and lignin along with 90.4% of silica removal from RS. It was noted that % lignin removal was higher for A (9.2%) as compared to D (0.54%), % extractives removal was lower for A and no change was reported for D. Quick lime used during preparation of petha sweet, resulted in increase of ash content due to absorption of Ca^2+^ ions in A^15^. EDX analysis indicated % calcium enhancement for A. As indicated in the EDX analysis, silica content was estimated for both A and D. The decrease in silica content is favourable for using RS for biofuel production and as well as fodder for cattle. Reducing sugar yield enhanced by five folds and 1.5 folds for A and D, respectively as compared to native RS which is due to the degradation of cellulose and hemicellulose^13^. Dev et al.^21^ used seawater as a reaction medium for microwave-NaOH pretreatment and saccharification of RS. This resulted in cellulose and sugar release of about 65.43% and 0.554 g/g, respectively. The cellulose release was comparable to our study as PWW pretreatment of RS have achieved about 53.16% cellulose without using any chemical. Decrease in VS of A was a result of enhanced ash content as compared to D. Moisture content was reduced for both A and D and reduction in protein content of both A and D was observed. Phosphorus and C/N ratio was enhanced for A which is good indicator for biofuel production^22^. Other components e.g. K, Fe, Zn, S also changed after pretreatment (Supplementary Information).

**Table 1:** Compositional analysis results of native (RS), PWW pretreated (A) and distilled water pretreated (D) rice straw with standard deviation.

Components analysed (%)	RS	A	D
Cellulose	43.04 ± 2.02	53.16 ± 1.84	44.79 ± 1.34
Hemicellulose	28.59 ± 1.7	21.36 ± 2.46	27.44 ± 1.47
Lignin	19.06 ± 1.3	9.86 ± 1.36	18.88 ± 1.05
Extractives	1.97 ± 0.32	1.57 ± 0.22	1.84 ± 0.43
Ash content	11.82 ± 0.65	17.67 ± 0.83	11.22 ± 0.31
Acid soluble silica	89.60 ± 3.56	55.30 ± 4.12	88.98 ± 4.73
Reducing sugar	3.26 ± 0.34	16.83 ± 1.24	5.28 ± 0.27
Volatile solids	88.28 ± 4.5	82.33 ± 4.26	88.78 ± 0.19
Crystallinity index	54.55 ± 0.68	31.90 ± 0.33	52.10 ± 0.51
Moisture content	4.29 ± 0.14	3.99 ± 0.13	4.21 ± 0.12
Protein	4.28 ± 0.20	3.02 ± 0.14	4.19 ± 0.17
Phosphorus	0.09 ± 0.02	0.25 ± 0.08	0.08 ± 0.03
Nitrogen	0.71 ± 0.10	0.52 ± 0.05	0.65 ± 0.07
C/N ratio	45.34 ± 1.12	79.98 ± 1.33	64.09 ± 1.01
Potassium	1.61 ± 0.11	1.03 ± 0.07	1.32 ± 0.16
Iron*	727.62 ± 0.23	501.40 ± 0.25	654.21 ± 0.12
Zinc*	14.96 ± 0.08	18.07 ± 0.11	14.45 ± 0.07
Sulphur*	17.81 ± 0.13	17.62 ± 0.27	17.21 ± 0.09

*μg/g_Dry weight_.

### Scanning electron microscopy (SEM)

**Figure 1:**
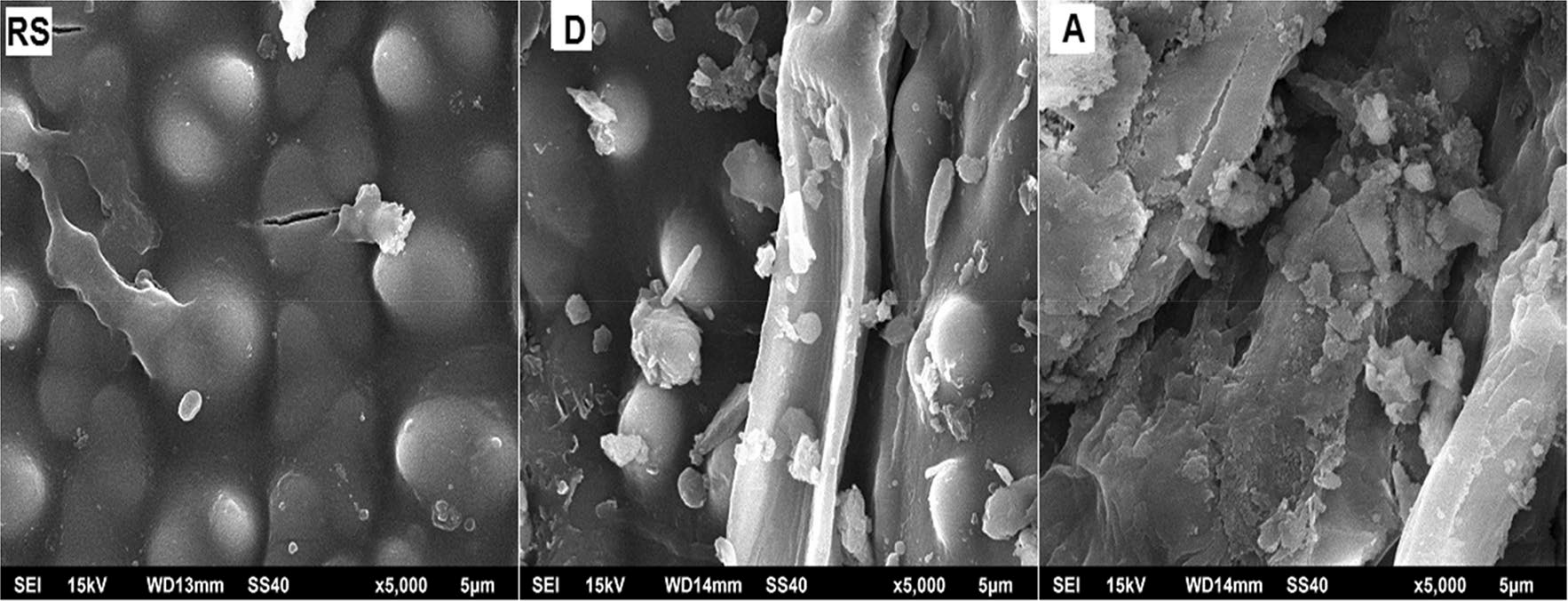
Scanning electron micrograph of native (**RS**), distilled water pretreated (**D**) and PWW pretreated (**A**) rice straw.

Scanning electron micrographs (JEOL JSM-6510LV, Tokyo, Japan) are shown for RS, D and A in Fig. 1 which indicate the changes in the surface morphology of RS. The surface roughness was enhanced after pretreatment which was higher for A as compared to D. Hence, PWW was more effective in enhancing the surface area of RS which is favourable for enzymatic and microbial attack leading biofuel production. This increment in surface area of RS can be attributed to lignin and hemicellulose degradation which increases the amorphous nature of cellulose^15^.

### Energy dispersive X-ray spectroscopy (EDX) analysis

**Figure 2:**
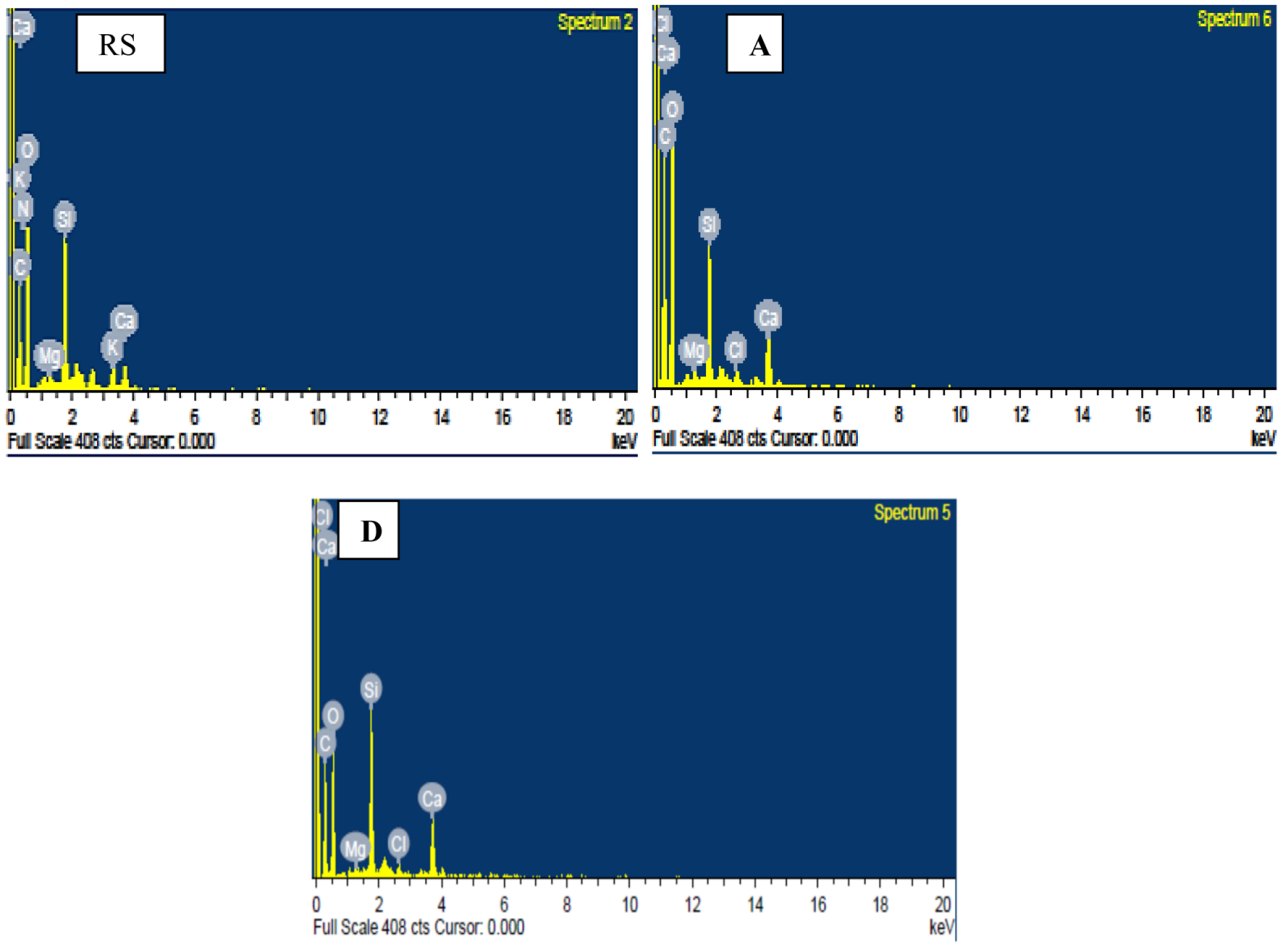
Energy dispersive X-ray spectrographs of native (**RS**), PWW pretreated (**A**) and distilled water pretreated (**D**) rice straw.

EDX spectrum was obtained by using energy dispersive X-ray spectrometer coupled with SEM (Oxford INCAx-act) and spectra for native and pretreated RS is shown in Fig. 2. Change in elemental composition was monitored using EDX. Spectrum showed enhancement in C content (32.19% to 41.59% and 41.66% for A and D, respectively) and Ca content (2.07% to 4.87% and 2.97% for A and D, respectively). Malik et al.^23^ reported an improvement in the digestibility of RS by pretreatment with lime (CaO) with an enhanced Ca content. Decrease in Oxygen content (52.90% to 50.08% and 44.92% for A and D, respectively), Si content (8.68% in RS to 4.30% and 7.72% for A and D, respectively), Mg content (0.58% in RS to 0.38% and 0.23% for A and D, respectively) and Cl content (1.01% in RS to 0.70% and 0.60%, respectively) were observed in the present study. Decrease in % Si content favours enhanced biofuel (ethanol) yield^13^. Khaleghian et al.^24^ reported about 91% silica removal by alkali pretreatment of RS with calcium carbonate (CaCO_3_) to enhance the enzymatic hydrolysis for bioethanol production.

### Fourier transform infrared (FTIR) spectroscopy

**Figure 3:**
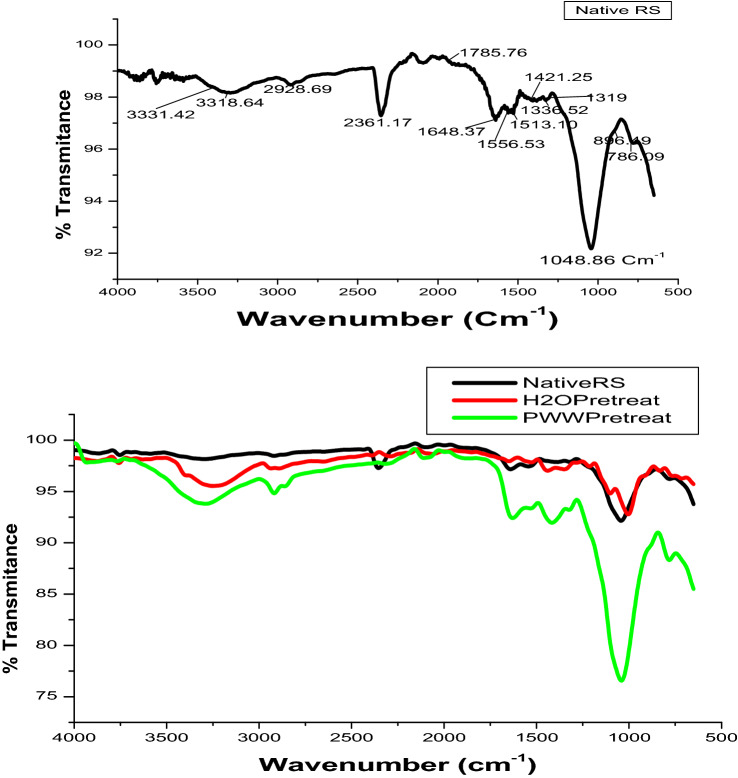
FTIR spectrum of native, PWW pretreated and distilled water (H_2_O) pretreated RS.

**Figure 4:**
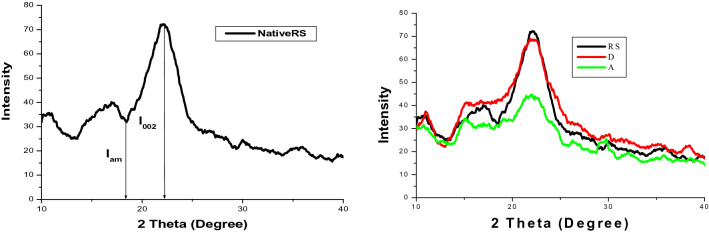
X-ray diffraction graphs for native (**RS**), PWW pretreated (**A**) and distilled water pretreated (**D**) rice straw.

FTIR spectrum of native and pretreated RS samples was recorded (Fig. 3). The first peak observed at 786 cm^−1^ corresponds to C–H group present in lignin and for out of plane vibration of C–H group in lignin. This effect was observed strongly in A as compared to D as reported by decrease in transmittance. Peaks at 896 cm^−1^ and 1048 cm^−1^ correspond to the C–H deformation in cellulose and C–O stretching in cellulose and hemicellulose, respectively^13,25^. Decrease in transmittance for C–H deformation and C–O stretching in A indicates the removal of cellulose and hemicellulose linked with lignin and each other. An increase in transmission was observed for D as compared to native RS indicating less lignin removal and the subsequent effectiveness of PWW for pretreatment of RS. Peaks at 1165 cm^−1^, 1239 cm^−1^ and 1265 cm^−1^ correspond to C–O–C vibrations in cellulose and hemicellulose^25^, C–O stretching of aryl group in lignin^26^ and C–O stretching in guaiacyl aromatic methoxyl groups of lignin along with ether linkage between lignin and carbohydrates^13,27^, respectively. A sharp decrease in transmittance or an increase in absorbance was observed for these groups for A, due to deformation of cellulose, hemicellulose and lignin linkage. Slight change was observed for D due to no effect on RS. Peaks at 1319 cm^−1^ and 1372 cm^−1^ correspond to pure cellulose^28^, 1325 cm^−1^ and 1375 cm^−1^ due to C–H vibration in cellulose and deformation of C–H linkage in cellulose and hemicellulose, respectively^13^. Decrease in these peaks indicates the increase in cellulose % which also corresponds to compositional analyses. Gabhane et al.^13^ observed similar peaks for enhancement in deformation of C–H linkage due to solubilisation of lignin. Peaks at 1421 cm^−1^ to 1435 cm^−1^ can be attributed to a symmetric CH_2_ bending vibration corresponding to crystalline cellulose^29,30^. Decrease in these peaks is due to reduction in the amount of crystalline cellulose which was also confirmed by the XRD pattern with decrease in % crystallinity index of pretreated RS samples (Fig. 4). Adsorption spectra at 1513 cm^−1^, 1601 cm^−1^ and 1648 cm^−1^ corresponds to C=O stretching of carbonyl related to hemicelluloses and lignin^28^, aromatic skeletal vibration^31^ and H–O–H bending of adsorption^25^, respectively.

The results show that C–H deformation enhanced after degradation of lignin in A, whereas removal of aromatic skeletal takes place in D. Peaks at 1741 cm^−1^ and 2916 cm^−1^ correspond to unconjugated C=O stretching in xylans^13,32^ and asymmetric stretching of CH_2_ group^33^, respectively. Decrease in these peaks in the present study was observed due to deformation and removal of hemicellulose which is also supported by compositional analyses. Sharp peaks at 3301 cm^−1^ and 3318 cm^−1^ are due to O–H stretching of lignin^13^ and hydrogen-bonded O–H groups^34^. This indicates the removal of lignin which was more apparent in A as compared to D. Table 2 represents FTIR spectral peak assignments for various functional groups and linkages.

**Table 2:** FTIR spectral peak assignments for various functional groups and linkages.

Bond assignments	Bond (cm^−1^)	% Transmittance		
		RS	A	D
Out of plane C–H vibration in lignin	786	96.10	88.09	97.17
Deformation of C–H linkage in cellulose	896	96.54	89.29	96.89
Stretching of C–O in cellulose and hemicellulose	1049	92.16	76.48	93.66
C–O–C vibrations in cellulose and hemicellulose	1165	96.18	87.57	97.39
C–O stretching of the aryl group in lignin	1239	97.56	92.22	97.96
C–O stretching in guaiacyl aromatic methoxyl groups	1265	98.03	93.86	97.87
Typical of pure cellulose	1319	97.60	92.68	97.53
C–H vibration in cellulose	1325	97.89	93.05	97.47
Typical of pure cellulose	1372	97.90	92.62	97.11
Deformation of C–H linkage in cellulose and hemicellulose	1375	97.60	92.38	96.76
A symmetric CH_2_ bending vibration attributed to crystalline cellulose	1435–1421	98.29–97.37	92.52–91.50	97.71–96.13
C=O stretching of carbonyl related to hemicelluloses and lignin	1513	97.73	93.34	97.29
Aromatic skeletal vibrations	1601	97.62	92.78	98.43
H–O–H bending of adsorption	1648	97.29	92.85	98.37
Unconjugated C=O stretching in xylans	1741	98.97	97.76	98.82
Asymmetric stretching of CH_2_ group^33^	2916	98.44	94.49	96.92
O–H stretching of lignin	3301	98.10	93.83	95.71
Hydrogen-bonded O–H groups^34^	3318	98.23	93.92	95.85

### Crystallinity index (CI)

X-ray spectrum of RS, D and A were recorded using X-ray diffractometer (Brucker, AXS D8 Advance made in Germany) in our previous study^15^ and graphs were plotted between intensity and 2θ (Theta) degree (Fig. 4). The crystallinity index (CI) was determined as the percentage of the crystalline material in biomass as shown in Eq. (1)^35^.

CI decreased from 54.55 to 30.90 for A and to 52.10 for D, respectively (Table 1) which may be due to conversion of crystalline cellulose into its amorphous form which aids in biodegradability of cellulose. Hence, 43.4% CI was decreased in A whereas only 4.5% CI decrease was observed in D as compared to native RS. Shifting of the peaks (I_002_) towards lower 2 theta values from 22.14 to 22.00 also corresponds to the conversion of crystalline cellulose into amorphous cellulose^36^. Gabhane et al.^13^ also reported the decrease in % crystallinity index for hydrothermal pretreatment of RS because of more solubilisation of hemicellulose rather than lignin from rice straw. Alkaline agents such as NaOH destroy cellulolytic areas with improved inner surface areas and pores ratio. Osman et al.^37^ reported CI of waste berry pomace as 37.5% due to the presence of large fraction of amorphous cellulose. About 23% decrease in CI was reported by Zhang et al.^38^ for methane production from yard waste by biological pretreatment.

1 $${\text{CI}} = ( {{\text{I}}_{{002}} - {\text{ I}}_{{{\text{am}}}} } ) \times 100\%/{\text{I}}_{{002}}$$

where: CI = Relative degree of crystallinity; I_002_ = Intensity of the diffraction from the 002 plane at 2θ = 22.4°; I_am_ = Intensity of the background scatter at 2θ = 18.5°.

### Phenolic compounds, Furfural and 5-hydroxymethylfurfural (HMF) analysis

Liquid samples (lignocellulosic hydrolysate) obtained after soaking of RS in distilled water and PWW were analysed for furfural, 5-hydroxymethylfurfural, and phenolic compounds using high performance liquid chromatography (HPLC)^39^. The results obtained from analyses revealed absence of furfural and HMF in the samples which is favourable for the biofuel production^40^. The detection limits used for the analysis were 0.1 g/100 L for furfural, 0.05% for phenolic compounds and 1 mg/kg for HMF. The concentration of furfural (3 g/L) has antagonistic effect on microbial growth for ethanol production^41^. 1.2 g/L furfural and 1.3 g/L HMF has been reported to decrease the cell growth very slightly when compared with reference fermentation^42^. Another study reported that furfural concentration less than 1 g/L is favourable for biohydrogen production, while a concentration more than 4 g/L is highly inhibitory^43^. Muñoz-Páez et al.^44^ evaluated the effect of fufural (0.10, 0.50, and 1.00 g/L) and HMF (0.02, 0.09, 0.19 g/L). They reported that furfural alone did not inhibit the hydrogen production; instead the inoculum completely degraded the furfural with the presence of furoic acid. HMF was partially degraded with its middle/low concentration and resulted in higher hydrogen production. Mixture of furfural and HMF had an inhibitory impact on hydrogen production. Prasad et al.^45^ pretreated wheat straw with 2–4% sulphuric acid at different temperatures. Maximum furfural and HMF were reported at 180–220 °C for 4% sulphuric acid pretreatment which was minimised by activated charcoal amendment for maximum bioethanol yield of 5.29% (v/v). A few batch studies were also performed for ethanol and methane production from PWW pretreated RS followed by microwave pretreatment in our lab^46^. We also explored various pretreatment methods for extraction of value added products from carbohydrate rich effluents and lignocellulosic biomass^47,48^. Studies on pretreatment of inoculum to enhance biohydrogen production have also been done^49^.

## Conclusion

The green pretreatment of RS done in the present study is very effective for economic and ecological biofuel production. Moreover, this green pretreatment does not result in phenolic compounds like furfural and 5-hydroxy methyl furfural, which are reported to be present in normal alkaline pretreatment, inhibit biofuel production and are toxic to the environment^50^. The effect of distilled water pretreatment on RS composition was negligible. PWW pretreatment was found efficient as compared to distilled water pretreatment. PWW pretreatment improved cellulose (43.04% for RS, 53.16% for A and 44.79% for D), increased reducing sugar (3.26% for RS, 16.83% for A and 5.28% for D) and lower lignin (19.06% for RS, 9.86% for A and 18.88% for D) yields. Results from SEM indicate improved surface area and porosity. EDX studies reveal that removal of silica was 4.38% for PWW. XRD analyses indicate that decrease in CI was about 43.4% for A whereas only 4.5% for D. Finally, FTIR revealed that pretreatment was more effective for A which provides further evidence for the efficacy of the green pretreatment of RS for bioethanol and other biofuel production. We also found PWW to be better substitute of an alkali for pretreatment of RS with negligible environmental impacts. Thereby PWW can be used in place of an alkali for pretreatment of lignocellulosic biomass in near future. There is no limitation of the proposed pretreatment process at lab scale but may occur at pilot scale plan. Further studies would be carried out to check the feasibility of the process at pilot scale.

## Supplementary Information

Supplementary Information.

## Data Availability

All data generated or analysed during this study are included in this published article and its supplementary information files.

## Abbreviations

RS: Rice straw
PWW: Petha wastewater
D: Distilled water pretreated rice straw
A: Petha wastewater pretreated rice straw
RON: Research octane number
CI: Crystallinity index
SEM: Scanning electron microscopy
EDX: Energy dispersive X-ray spectroscopy
FTIR: Fourier transform infrared
XRD: X-ray diffraction
HMF: 5-Hydroxymethylfurfural
