# Supplementary material for: Rice straw structure changes following green pretreatment with petha wastewater for economically viable bioethanol production
Source: Sci Rep. 2022 Jun 21;12:10443. doi: 10.1038/s41598-022-14627-7 (PMC9213452; doi:10.1038/s41598-022-14627-7)
Supplement: Supplementary file 1 — Supplementary Information. [file 41598_2022_14627_MOESM1_ESM.docx]

**Raw data Nature Paper**

**FTIR Plots of RS, A and D**

**
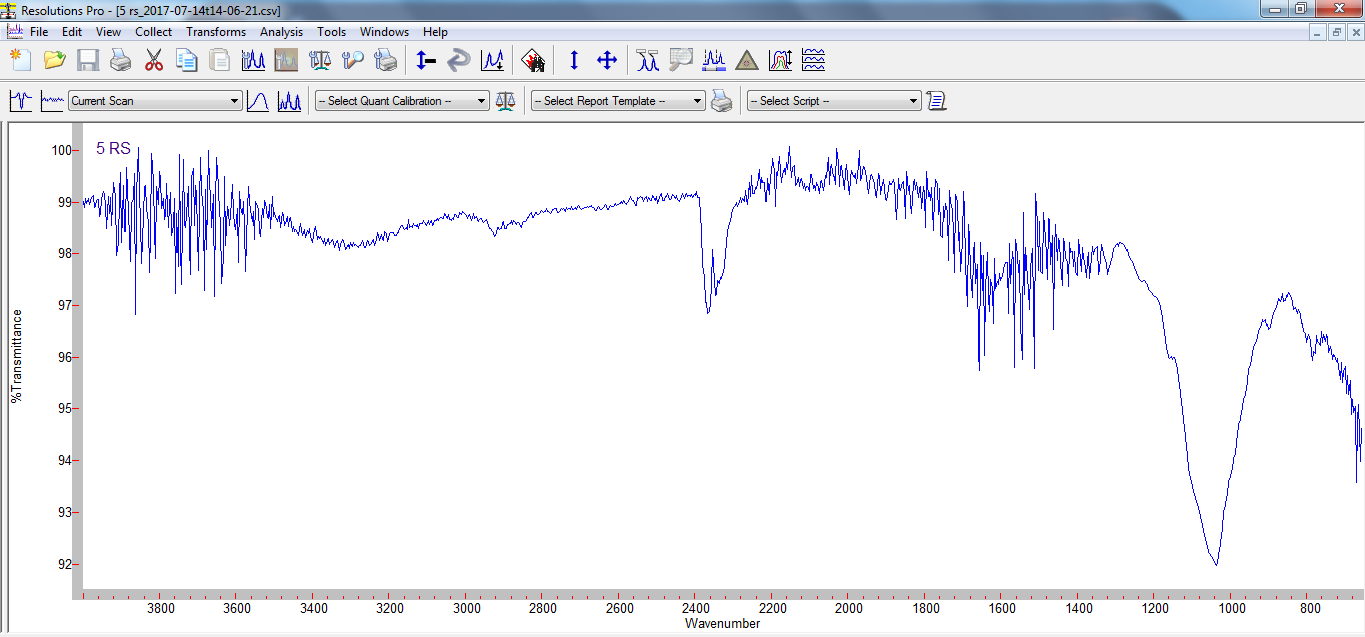
**

**Supplementary Figure 1: FTIR Plot of Native RS**

**
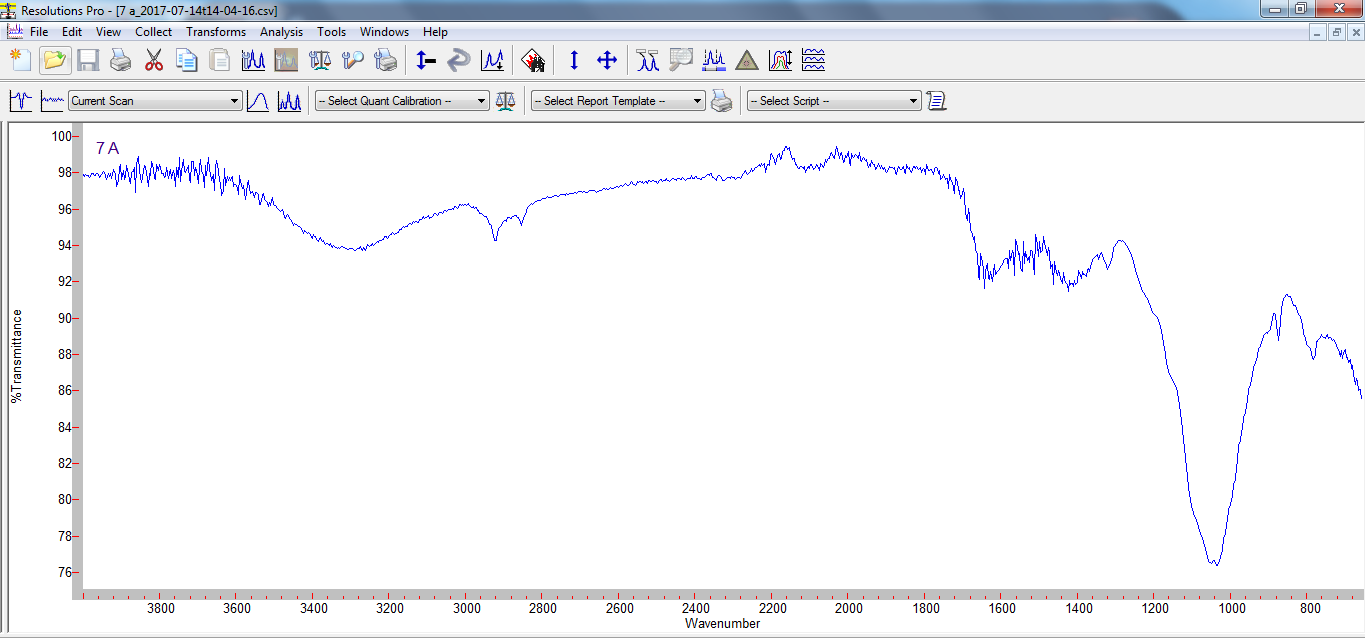
**

**Supplementary Figure 2: FTIR Plot of PWW pretreated RS (A)**

**
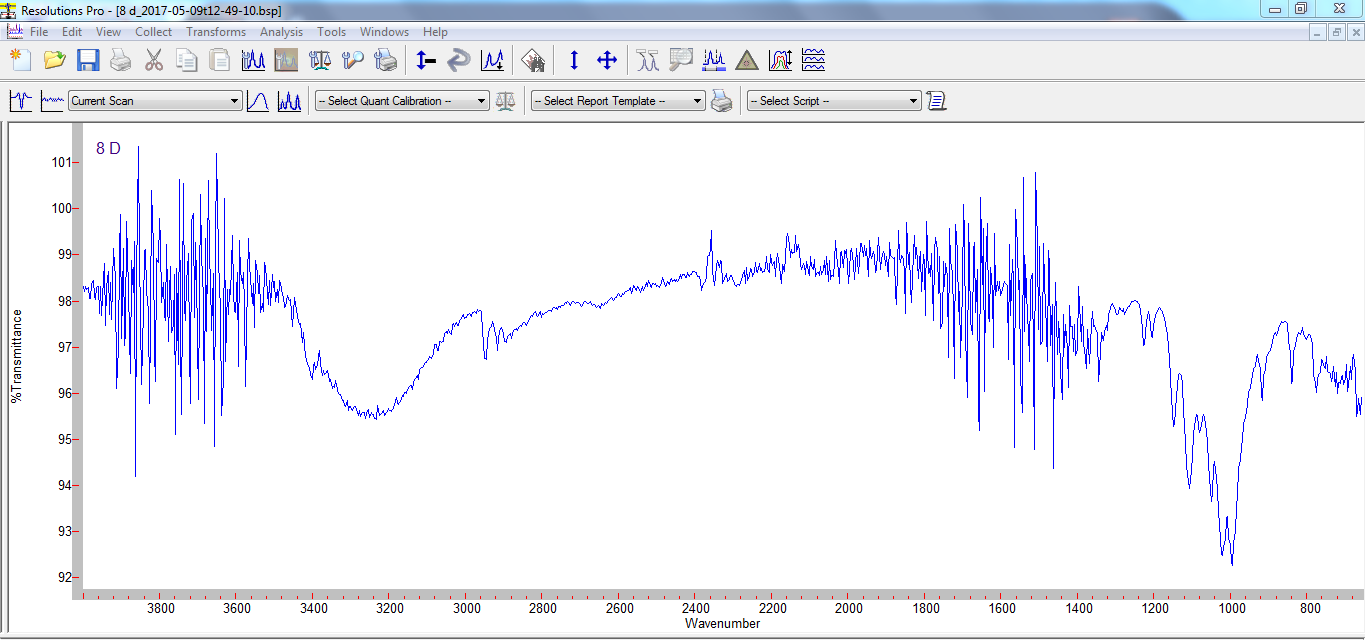
**

**Supplementary Figure 3: FTIR Plot of Distilled water pretreated RS (D)**

**Supplementary Table 1: FTIR raw data of RS, A and D**

| **RS** |  |  | **A** | Wavenumber | | **D** | Wavenumber |
| --- | --- | --- | --- | --- | --- | --- | --- |
| YLabel | %Transmittance | | YLabel | %Transmittance | | YLabel | %Transmittance |
| FileType | %Transmittance | | FileType | %Transmittance | | FileType | %Transmittance |
| DisplayDirection | 20300 |  | DisplayDirection | 20300 |  | DisplayDirection | 20300 |
| PeakDirection | 20311 |  | PeakDirection | 20311 |  | PeakDirection | 20311 |
| 650.61213 | 93.214197 |  | 650.61213 | 85.16625 |  | 650.61213 | 95.62309 |
| 652.47635 | 93.411907 |  | 652.47635 | 84.912933 |  | 652.47635 | 95.948982 |
| 654.34057 | 94.574544 |  | 654.34057 | 85.544453 |  | 654.34057 | 95.557329 |
| 656.20479 | 94.639528 |  | 656.20479 | 86.093496 |  | 656.20479 | 95.929478 |
| 658.069 | 93.985328 |  | 658.069 | 85.73773 |  | 658.069 | 95.905451 |
| 659.93322 | 94.458308 |  | 659.93322 | 85.836655 |  | 659.93322 | 95.539074 |
| 661.79744 | 95.086896 |  | 661.79744 | 86.184386 |  | 661.79744 | 95.89172 |
| 663.66166 | 94.819788 |  | 663.66166 | 86.007069 |  | 663.66166 | 95.895145 |
| 665.52588 | 94.804168 |  | 665.52588 | 86.539746 |  | 665.52588 | 95.593236 |
| 667.3901 | 93.913347 |  | 667.3901 | 86.705613 |  | 667.3901 | 95.487905 |
| 669.25431 | 93.572633 |  | 669.25431 | 86.358277 |  | 669.25431 | 95.711469 |
| 671.11853 | 94.911143 |  | 671.11853 | 86.692161 |  | 671.11853 | 96.221394 |
| 672.98275 | 95.05513 |  | 672.98275 | 86.657691 |  | 672.98275 | 96.478027 |
| 674.84697 | 94.938273 |  | 674.84697 | 86.899372 |  | 674.84697 | 96.740121 |
| 676.71119 | 95.175496 |  | 676.71119 | 87.254216 |  | 676.71119 | 96.840586 |
| 678.5754 | 95.188044 |  | 678.5754 | 87.386096 |  | 678.5754 | 96.712664 |
| 680.43962 | 94.971611 |  | 680.43962 | 87.398126 |  | 680.43962 | 96.295183 |
| 682.30384 | 94.901153 |  | 682.30384 | 87.221463 |  | 682.30384 | 96.30321 |
| 684.16806 | 95.244725 |  | 684.16806 | 87.29455 |  | 684.16806 | 96.577809 |
| 686.03228 | 95.546392 |  | 686.03228 | 87.725421 |  | 686.03228 | 96.328063 |
| 687.89649 | 95.313495 |  | 687.89649 | 87.696587 |  | 687.89649 | 96.196268 |
| 689.76071 | 95.277681 |  | 689.76071 | 87.545478 |  | 689.76071 | 96.172469 |
| 691.62493 | 95.64812 |  | 691.62493 | 87.816982 |  | 691.62493 | 96.033998 |
| 693.48915 | 95.631949 |  | 693.48915 | 87.984001 |  | 693.48915 | 96.407776 |
| 695.35337 | 95.291118 |  | 695.35337 | 87.830425 |  | 695.35337 | 96.631748 |
| 697.21759 | 95.406176 |  | 697.21759 | 87.859423 |  | 697.21759 | 96.49701 |
| 699.0818 | 95.811951 |  | 699.0818 | 88.258108 |  | 699.0818 | 96.327285 |
| 700.94602 | 95.718139 |  | 700.94602 | 88.138671 |  | 700.94602 | 96.194979 |
| 702.81024 | 95.513436 |  | 702.81024 | 87.783028 |  | 702.81024 | 96.194859 |
| 704.67446 | 95.708637 |  | 704.67446 | 88.002111 |  | 704.67446 | 96.375218 |
| 706.53868 | 95.903345 |  | 706.53868 | 88.211863 |  | 706.53868 | 96.372287 |
| 708.40289 | 95.730148 |  | 708.40289 | 87.927147 |  | 708.40289 | 96.252032 |
| 710.26711 | 95.572166 |  | 710.26711 | 87.877373 |  | 710.26711 | 96.202778 |
| 712.13133 | 95.636508 |  | 712.13133 | 88.173005 |  | 712.13133 | 96.259351 |
| 713.99555 | 95.819284 |  | 713.99555 | 88.259521 |  | 713.99555 | 96.22498 |
| 715.85977 | 95.983527 |  | 715.85977 | 88.296096 |  | 715.85977 | 96.106321 |
| 717.72398 | 95.884242 |  | 717.72398 | 88.360849 |  | 717.72398 | 96.004151 |
| 719.5882 | 95.911864 |  | 719.5882 | 88.569562 |  | 719.5882 | 96.269425 |
| 721.45242 | 95.931304 |  | 721.45242 | 88.583969 |  | 721.45242 | 96.458116 |
| 723.31664 | 95.968313 |  | 723.31664 | 88.554824 |  | 723.31664 | 96.237957 |
| 725.18086 | 95.98903 |  | 725.18086 | 88.675083 |  | 725.18086 | 96.137133 |
| 727.04508 | 96.047809 |  | 727.04508 | 88.81306 |  | 727.04508 | 96.447326 |
| 728.90929 | 95.995269 |  | 728.90929 | 88.775922 |  | 728.90929 | 96.581387 |
| 730.77351 | 95.965592 |  | 730.77351 | 88.770561 |  | 730.77351 | 96.360633 |
| 732.63773 | 96.138553 |  | 732.63773 | 88.91528 |  | 732.63773 | 96.261155 |
| 734.50195 | 96.207114 |  | 734.50195 | 88.938619 |  | 734.50195 | 96.320094 |
| 736.36617 | 96.155911 |  | 736.36617 | 88.900209 |  | 736.36617 | 96.369452 |
| 738.23038 | 96.172167 |  | 738.23038 | 88.96012 |  | 738.23038 | 96.436915 |
| 740.0946 | 96.086085 |  | 740.0946 | 88.8731 |  | 740.0946 | 96.445813 |
| 741.95882 | 96.217478 |  | 741.95882 | 88.981425 |  | 741.95882 | 96.498993 |
| 743.82304 | 96.402358 |  | 743.82304 | 89.117951 |  | 743.82304 | 96.500366 |
| 745.68726 | 96.435125 |  | 745.68726 | 89.003023 |  | 745.68726 | 96.63304 |
| 747.55148 | 96.351951 |  | 747.55148 | 88.865262 |  | 747.55148 | 96.798513 |
| 749.41569 | 96.295972 |  | 749.41569 | 88.87686 |  | 749.41569 | 96.726007 |
| 751.27991 | 96.340559 |  | 751.27991 | 88.865011 |  | 751.27991 | 96.529647 |
| 753.14413 | 96.438278 |  | 753.14413 | 88.967811 |  | 753.14413 | 96.591364 |
| 755.00835 | 96.314526 |  | 755.00835 | 88.993983 |  | 755.00835 | 96.70305 |
| 756.87257 | 96.290018 |  | 756.87257 | 89.004875 |  | 756.87257 | 96.680523 |
| 758.73678 | 96.500739 |  | 758.73678 | 89.108471 |  | 758.73678 | 96.679493 |
| 760.601 | 96.489652 |  | 760.601 | 89.083617 |  | 760.601 | 96.740295 |
| 762.46522 | 96.26898 |  | 762.46522 | 88.97942 |  | 762.46522 | 96.663476 |
| 764.32944 | 96.233865 |  | 764.32944 | 88.927673 |  | 764.32944 | 96.459703 |
| 766.19366 | 96.292698 |  | 766.19366 | 88.854176 |  | 766.19366 | 96.371163 |
| 768.05787 | 96.280926 |  | 768.05787 | 88.764013 |  | 768.05787 | 96.412972 |
| 769.92209 | 96.309574 |  | 769.92209 | 88.706521 |  | 769.92209 | 96.303693 |
| 771.78631 | 96.415965 |  | 771.78631 | 88.695703 |  | 771.78631 | 96.134149 |
| 773.65053 | 96.297059 |  | 773.65053 | 88.435551 |  | 773.65053 | 96.017135 |
| 775.51475 | 96.068808 |  | 775.51475 | 88.092457 |  | 775.51475 | 96.043653 |
| 777.37897 | 96.10452 |  | 777.37897 | 87.955314 |  | 777.37897 | 96.205226 |
| 779.24318 | 96.113218 |  | 779.24318 | 87.81891 |  | 779.24318 | 96.404304 |
| 781.1074 | 95.989162 |  | 781.1074 | 87.755565 |  | 781.1074 | 96.591927 |
| 782.97162 | 95.945685 |  | 782.97162 | 87.768355 |  | 782.97162 | 96.883219 |
| 784.83584 | 95.944945 |  | 784.83584 | 87.830185 |  | 784.83584 | 97.088352 |
| 786.70006 | 96.09969 |  | 786.70006 | 88.086148 |  | 786.70006 | 97.168171 |
| 788.56427 | 96.226729 |  | 788.56427 | 88.246612 |  | 788.56427 | 97.233602 |
| 790.42849 | 96.322176 |  | 790.42849 | 88.342509 |  | 790.42849 | 97.262425 |
| 792.29271 | 96.363601 |  | 792.29271 | 88.452609 |  | 792.29271 | 97.265746 |
| 794.15693 | 96.400644 |  | 794.15693 | 88.469194 |  | 794.15693 | 97.237938 |
| 796.02115 | 96.438094 |  | 796.02115 | 88.478196 |  | 796.02115 | 97.213862 |
| 797.88536 | 96.355404 |  | 797.88536 | 88.526457 |  | 797.88536 | 97.258173 |
| 799.74958 | 96.284915 |  | 799.74958 | 88.59514 |  | 799.74958 | 97.185355 |
| 801.6138 | 96.372043 |  | 801.6138 | 88.753407 |  | 801.6138 | 97.167362 |
| 803.47802 | 96.45771 |  | 803.47802 | 88.967603 |  | 803.47802 | 97.203949 |
| 805.34224 | 96.500989 |  | 805.34224 | 89.162573 |  | 805.34224 | 97.279399 |
| 807.20646 | 96.600382 |  | 807.20646 | 89.363987 |  | 807.20646 | 97.409815 |
| 809.07067 | 96.646112 |  | 809.07067 | 89.529962 |  | 809.07067 | 97.429312 |
| 810.93489 | 96.648055 |  | 810.93489 | 89.686684 |  | 810.93489 | 97.319711 |
| 812.79911 | 96.657835 |  | 812.79911 | 89.830344 |  | 812.79911 | 97.311683 |
| 814.66333 | 96.68978 |  | 814.66333 | 89.952139 |  | 814.66333 | 97.308517 |
| 816.52755 | 96.844791 |  | 816.52755 | 90.108154 |  | 816.52755 | 97.281767 |
| 818.39176 | 96.914055 |  | 818.39176 | 90.184818 |  | 818.39176 | 97.265851 |
| 820.25598 | 96.891439 |  | 820.25598 | 90.227667 |  | 820.25598 | 97.318487 |
| 822.1202 | 96.853974 |  | 822.1202 | 90.304501 |  | 822.1202 | 97.32311 |
| 823.98442 | 96.856352 |  | 823.98442 | 90.412842 |  | 823.98442 | 97.234254 |
| 825.84864 | 96.913354 |  | 825.84864 | 90.530371 |  | 825.84864 | 97.192826 |
| 827.71286 | 96.950171 |  | 827.71286 | 90.632935 |  | 827.71286 | 97.179454 |
| 829.57707 | 96.965476 |  | 829.57707 | 90.711572 |  | 829.57707 | 97.038011 |
| 831.44129 | 96.930033 |  | 831.44129 | 90.713387 |  | 831.44129 | 96.903442 |
| 833.30551 | 96.999474 |  | 833.30551 | 90.785537 |  | 833.30551 | 96.81093 |
| 835.16973 | 97.057011 |  | 835.16973 | 90.89213 |  | 835.16973 | 96.585006 |
| 837.03395 | 97.067131 |  | 837.03395 | 90.923191 |  | 837.03395 | 96.26545 |
| 838.89816 | 97.16155 |  | 838.89816 | 91.020669 |  | 838.89816 | 96.221112 |
| 840.76238 | 97.197238 |  | 840.76238 | 91.14982 |  | 840.76238 | 96.507515 |
| 842.6266 | 97.175513 |  | 842.6266 | 91.174607 |  | 842.6266 | 96.886814 |
| 844.49082 | 97.208869 |  | 844.49082 | 91.174989 |  | 844.49082 | 97.140127 |
| 846.35504 | 97.256431 |  | 846.35504 | 91.247955 |  | 846.35504 | 97.322657 |
| 848.21925 | 97.18391 |  | 848.21925 | 91.250116 |  | 848.21925 | 97.449199 |
| 850.08347 | 97.16986 |  | 850.08347 | 91.286713 |  | 850.08347 | 97.509169 |
| 851.94769 | 97.185753 |  | 851.94769 | 91.313232 |  | 851.94769 | 97.529163 |
| 853.81191 | 97.142779 |  | 853.81191 | 91.268287 |  | 853.81191 | 97.550651 |
| 855.67613 | 97.09636 |  | 855.67613 | 91.195068 |  | 855.67613 | 97.545983 |
| 857.54035 | 97.079473 |  | 857.54035 | 91.088796 |  | 857.54035 | 97.542846 |
| 859.40456 | 97.124263 |  | 859.40456 | 90.939785 |  | 859.40456 | 97.535801 |
| 861.26878 | 97.221932 |  | 861.26878 | 90.851546 |  | 861.26878 | 97.523908 |
| 863.133 | 97.223889 |  | 863.133 | 90.748094 |  | 863.133 | 97.530874 |
| 864.99722 | 97.137196 |  | 864.99722 | 90.566361 |  | 864.99722 | 97.561047 |
| 866.86144 | 97.079855 |  | 866.86144 | 90.26839 |  | 866.86144 | 97.550488 |
| 868.72565 | 97.122581 |  | 868.72565 | 89.791325 |  | 868.72565 | 97.488049 |
| 870.58987 | 97.144522 |  | 870.58987 | 89.195029 |  | 870.58987 | 97.411751 |
| 872.45409 | 97.076776 |  | 872.45409 | 88.780641 |  | 872.45409 | 97.396883 |
| 874.31831 | 97.047043 |  | 874.31831 | 88.866447 |  | 874.31831 | 97.3701 |
| 876.18253 | 97.035095 |  | 876.18253 | 89.263169 |  | 876.18253 | 97.292641 |
| 878.04675 | 96.973179 |  | 878.04675 | 89.624817 |  | 878.04675 | 97.261127 |
| 879.91096 | 96.923471 |  | 879.91096 | 89.948673 |  | 879.91096 | 97.321841 |
| 881.77518 | 96.922727 |  | 881.77518 | 90.218397 |  | 881.77518 | 97.298771 |
| 883.6394 | 96.852379 |  | 883.6394 | 90.271488 |  | 883.6394 | 97.253858 |
| 885.50362 | 96.802637 |  | 885.50362 | 90.196942 |  | 885.50362 | 97.193758 |
| 887.36784 | 96.772299 |  | 887.36784 | 90.05582 |  | 887.36784 | 97.160237 |
| 889.23205 | 96.760235 |  | 889.23205 | 89.90739 |  | 889.23205 | 97.103301 |
| 891.09627 | 96.704829 |  | 891.09627 | 89.740668 |  | 891.09627 | 97.021131 |
| 892.96049 | 96.600205 |  | 892.96049 | 89.53136 |  | 892.96049 | 96.958085 |
| 894.82471 | 96.573272 |  | 894.82471 | 89.40579 |  | 894.82471 | 96.929995 |
| 896.68893 | 96.544883 |  | 896.68893 | 89.293844 |  | 896.68893 | 96.889682 |
| 898.55314 | 96.58928 |  | 898.55314 | 89.250507 |  | 898.55314 | 96.879376 |
| 900.41736 | 96.626092 |  | 900.41736 | 89.223946 |  | 900.41736 | 96.837958 |
| 902.28158 | 96.609385 |  | 902.28158 | 89.185839 |  | 902.28158 | 96.82578 |
| 904.1458 | 96.621255 |  | 904.1458 | 89.191051 |  | 904.1458 | 96.787896 |
| 906.01002 | 96.685139 |  | 906.01002 | 89.210897 |  | 906.01002 | 96.654916 |
| 907.87424 | 96.731175 |  | 907.87424 | 89.173425 |  | 907.87424 | 96.515432 |
| 909.73845 | 96.694784 |  | 909.73845 | 89.05035 |  | 909.73845 | 96.389373 |
| 911.60267 | 96.688711 |  | 911.60267 | 88.923285 |  | 911.60267 | 96.195635 |
| 913.46689 | 96.727243 |  | 913.46689 | 88.839127 |  | 913.46689 | 95.973492 |
| 915.33111 | 96.689624 |  | 915.33111 | 88.755746 |  | 915.33111 | 95.848059 |
| 917.19533 | 96.624943 |  | 917.19533 | 88.661244 |  | 917.19533 | 95.968571 |
| 919.05954 | 96.576959 |  | 919.05954 | 88.560594 |  | 919.05954 | 96.250552 |
| 920.92376 | 96.566703 |  | 920.92376 | 88.464791 |  | 920.92376 | 96.526577 |
| 922.78798 | 96.526764 |  | 922.78798 | 88.340782 |  | 922.78798 | 96.717214 |
| 924.6522 | 96.489487 |  | 924.6522 | 88.207055 |  | 924.6522 | 96.818806 |
| 926.51642 | 96.461596 |  | 926.51642 | 88.052905 |  | 926.51642 | 96.824763 |
| 928.38063 | 96.386727 |  | 928.38063 | 87.908594 |  | 928.38063 | 96.843336 |
| 930.24485 | 96.305384 |  | 930.24485 | 87.749234 |  | 930.24485 | 96.767455 |
| 932.10907 | 96.287745 |  | 932.10907 | 87.633324 |  | 932.10907 | 96.651748 |
| 933.97329 | 96.256423 |  | 933.97329 | 87.499512 |  | 933.97329 | 96.581442 |
| 935.83751 | 96.187685 |  | 935.83751 | 87.318328 |  | 935.83751 | 96.540302 |
| 937.70173 | 96.138618 |  | 937.70173 | 87.161964 |  | 937.70173 | 96.510826 |
| 939.56594 | 96.12722 |  | 939.56594 | 87.011555 |  | 939.56594 | 96.469338 |
| 941.43016 | 96.066719 |  | 941.43016 | 86.837732 |  | 941.43016 | 96.365551 |
| 943.29438 | 95.957487 |  | 943.29438 | 86.661063 |  | 943.29438 | 96.286218 |
| 945.1586 | 95.883011 |  | 945.1586 | 86.462451 |  | 945.1586 | 96.183395 |
| 947.02282 | 95.836458 |  | 947.02282 | 86.27413 |  | 947.02282 | 96.10421 |
| 948.88703 | 95.774828 |  | 948.88703 | 86.071707 |  | 948.88703 | 96.036984 |
| 950.75125 | 95.72201 |  | 950.75125 | 85.862296 |  | 950.75125 | 95.96001 |
| 952.61547 | 95.618435 |  | 952.61547 | 85.645206 |  | 952.61547 | 95.873144 |
| 954.47969 | 95.517296 |  | 954.47969 | 85.42124 |  | 954.47969 | 95.7392 |
| 956.34391 | 95.417363 |  | 956.34391 | 85.194157 |  | 956.34391 | 95.629415 |
| 958.20813 | 95.343854 |  | 958.20813 | 84.978875 |  | 958.20813 | 95.568311 |
| 960.07234 | 95.26084 |  | 960.07234 | 84.753095 |  | 960.07234 | 95.46292 |
| 961.93656 | 95.202651 |  | 961.93656 | 84.5591 |  | 961.93656 | 95.342743 |
| 963.80078 | 95.161717 |  | 963.80078 | 84.390945 |  | 963.80078 | 95.206569 |
| 965.665 | 95.079229 |  | 965.665 | 84.165519 |  | 965.665 | 95.086028 |
| 967.52922 | 95.003608 |  | 967.52922 | 83.946026 |  | 967.52922 | 94.974351 |
| 969.39343 | 94.956098 |  | 969.39343 | 83.745773 |  | 969.39343 | 94.863171 |
| 971.25765 | 94.889975 |  | 971.25765 | 83.521635 |  | 971.25765 | 94.714271 |
| 973.12187 | 94.795808 |  | 973.12187 | 83.280433 |  | 973.12187 | 94.535959 |
| 974.98609 | 94.700919 |  | 974.98609 | 83.016572 |  | 974.98609 | 94.362637 |
| 976.85031 | 94.618846 |  | 976.85031 | 82.735139 |  | 976.85031 | 94.202011 |
| 978.71452 | 94.536814 |  | 978.71452 | 82.446635 |  | 978.71452 | 94.007381 |
| 980.57874 | 94.479904 |  | 980.57874 | 82.14313 |  | 980.57874 | 93.783016 |
| 982.44296 | 94.37642 |  | 982.44296 | 81.815559 |  | 982.44296 | 93.533185 |
| 984.30718 | 94.240663 |  | 984.30718 | 81.45862 |  | 984.30718 | 93.270972 |
| 986.1714 | 94.127181 |  | 986.1714 | 81.124874 |  | 986.1714 | 92.990617 |
| 988.03562 | 94.050773 |  | 988.03562 | 80.847438 |  | 988.03562 | 92.765796 |
| 989.89983 | 93.949023 |  | 989.89983 | 80.583891 |  | 989.89983 | 92.552307 |
| 991.76405 | 93.870143 |  | 991.76405 | 80.377376 |  | 991.76405 | 92.367358 |
| 993.62827 | 93.823543 |  | 993.62827 | 80.196015 |  | 993.62827 | 92.257328 |
| 995.49249 | 93.791474 |  | 995.49249 | 80.034815 |  | 995.49249 | 92.323853 |
| 997.35671 | 93.743553 |  | 997.35671 | 79.871807 |  | 997.35671 | 92.523782 |
| 999.22092 | 93.673102 |  | 999.22092 | 79.68979 |  | 999.22092 | 92.713377 |
| 1001.0851 | 93.590693 |  | 1001.0851 | 79.474283 |  | 1001.0851 | 92.852087 |
| 1002.9494 | 93.505617 |  | 1002.9494 | 79.237435 |  | 1002.9494 | 93.021895 |
| 1004.8136 | 93.41741 |  | 1004.8136 | 79.022797 |  | 1004.8136 | 93.194516 |
| 1006.6778 | 93.354399 |  | 1006.6778 | 78.846505 |  | 1006.6778 | 93.314796 |
| 1008.542 | 93.282727 |  | 1008.542 | 78.656153 |  | 1008.542 | 93.281661 |
| 1010.4062 | 93.221399 |  | 1010.4062 | 78.431525 |  | 1010.4062 | 93.076876 |
| 1012.2705 | 93.141597 |  | 1012.2705 | 78.159266 |  | 1012.2705 | 92.824225 |
| 1014.1347 | 93.012548 |  | 1014.1347 | 77.875305 |  | 1014.1347 | 92.656668 |
| 1015.9989 | 92.875091 |  | 1015.9989 | 77.621831 |  | 1015.9989 | 92.605554 |
| 1017.8631 | 92.787799 |  | 1017.8631 | 77.432606 |  | 1017.8631 | 92.555941 |
| 1019.7273 | 92.679475 |  | 1019.7273 | 77.233766 |  | 1019.7273 | 92.473979 |
| 1021.5915 | 92.545451 |  | 1021.5915 | 77.031364 |  | 1021.5915 | 92.509463 |
| 1023.4558 | 92.427143 |  | 1023.4558 | 76.849822 |  | 1023.4558 | 92.689634 |
| 1025.32 | 92.343732 |  | 1025.32 | 76.724944 |  | 1025.32 | 92.927992 |
| 1027.1842 | 92.260002 |  | 1027.1842 | 76.616633 |  | 1027.1842 | 93.159392 |
| 1029.0484 | 92.187846 |  | 1029.0484 | 76.533621 |  | 1029.0484 | 93.423368 |
| 1030.9126 | 92.098512 |  | 1030.9126 | 76.449059 |  | 1030.9126 | 93.662464 |
| 1032.7769 | 92.012529 |  | 1032.7769 | 76.386192 |  | 1032.7769 | 93.901351 |
| 1034.6411 | 91.969007 |  | 1034.6411 | 76.380481 |  | 1034.6411 | 94.178172 |
| 1036.5053 | 91.991564 |  | 1036.5053 | 76.449579 |  | 1036.5053 | 94.408891 |
| 1038.3695 | 92.023465 |  | 1038.3695 | 76.555742 |  | 1038.3695 | 94.51186 |
| 1040.2337 | 92.047657 |  | 1040.2337 | 76.645897 |  | 1040.2337 | 94.508078 |
| 1042.0979 | 92.068351 |  | 1042.0979 | 76.665307 |  | 1042.0979 | 94.398419 |
| 1043.9622 | 92.105433 |  | 1043.9622 | 76.614914 |  | 1043.9622 | 94.159667 |
| 1045.8264 | 92.135254 |  | 1045.8264 | 76.535628 |  | 1045.8264 | 93.84792 |
| 1047.6906 | 92.141193 |  | 1047.6906 | 76.497623 |  | 1047.6906 | 93.658924 |
| 1049.5548 | 92.16199 |  | 1049.5548 | 76.484318 |  | 1049.5548 | 93.659034 |
| 1051.419 | 92.187421 |  | 1051.419 | 76.498723 |  | 1051.419 | 93.850396 |
| 1053.2833 | 92.208225 |  | 1053.2833 | 76.554634 |  | 1053.2833 | 94.155988 |
| 1055.1475 | 92.246979 |  | 1055.1475 | 76.649058 |  | 1055.1475 | 94.464504 |
| 1057.0117 | 92.286858 |  | 1057.0117 | 76.759268 |  | 1057.0117 | 94.695936 |
| 1058.8759 | 92.34584 |  | 1058.8759 | 76.891424 |  | 1058.8759 | 94.929121 |
| 1060.7401 | 92.391818 |  | 1060.7401 | 77.042501 |  | 1060.7401 | 95.174339 |
| 1062.6043 | 92.441689 |  | 1062.6043 | 77.209097 |  | 1062.6043 | 95.331426 |
| 1064.4686 | 92.489928 |  | 1064.4686 | 77.375192 |  | 1064.4686 | 95.40143 |
| 1066.3328 | 92.566579 |  | 1066.3328 | 77.546786 |  | 1066.3328 | 95.471629 |
| 1068.197 | 92.646582 |  | 1068.197 | 77.684341 |  | 1068.197 | 95.527935 |
| 1070.0612 | 92.702374 |  | 1070.0612 | 77.813117 |  | 1070.0612 | 95.539119 |
| 1071.9254 | 92.767751 |  | 1071.9254 | 77.923392 |  | 1071.9254 | 95.460197 |
| 1073.7896 | 92.819639 |  | 1073.7896 | 78.006006 |  | 1073.7896 | 95.309155 |
| 1075.6539 | 92.847464 |  | 1075.6539 | 78.0937 |  | 1075.6539 | 95.18171 |
| 1077.5181 | 92.923136 |  | 1077.5181 | 78.205809 |  | 1077.5181 | 95.141972 |
| 1079.3823 | 93.01264 |  | 1079.3823 | 78.340436 |  | 1079.3823 | 95.148839 |
| 1081.2465 | 93.084874 |  | 1081.2465 | 78.489299 |  | 1081.2465 | 95.275804 |
| 1083.1107 | 93.136805 |  | 1083.1107 | 78.622556 |  | 1083.1107 | 95.417031 |
| 1084.975 | 93.174279 |  | 1084.975 | 78.741823 |  | 1084.975 | 95.509468 |
| 1086.8392 | 93.212384 |  | 1086.8392 | 78.854404 |  | 1086.8392 | 95.545961 |
| 1088.7034 | 93.262143 |  | 1088.7034 | 78.958487 |  | 1088.7034 | 95.518229 |
| 1090.5676 | 93.312251 |  | 1090.5676 | 79.053246 |  | 1090.5676 | 95.426019 |
| 1092.4318 | 93.36298 |  | 1092.4318 | 79.125109 |  | 1092.4318 | 95.293855 |
| 1094.296 | 93.395295 |  | 1094.296 | 79.227886 |  | 1094.296 | 95.113283 |
| 1096.1603 | 93.456739 |  | 1096.1603 | 79.363689 |  | 1096.1603 | 94.905853 |
| 1098.0245 | 93.523276 |  | 1098.0245 | 79.502518 |  | 1098.0245 | 94.639571 |
| 1099.8887 | 93.574959 |  | 1099.8887 | 79.662688 |  | 1099.8887 | 94.328289 |
| 1101.7529 | 93.633137 |  | 1101.7529 | 79.830851 |  | 1101.7529 | 94.060696 |
| 1103.6171 | 93.697869 |  | 1103.6171 | 80.052325 |  | 1103.6171 | 93.942843 |
| 1105.4814 | 93.749065 |  | 1105.4814 | 80.335308 |  | 1105.4814 | 93.97832 |
| 1107.3456 | 93.841309 |  | 1107.3456 | 80.649643 |  | 1107.3456 | 94.095271 |
| 1109.2098 | 93.97283 |  | 1109.2098 | 81.000486 |  | 1109.2098 | 94.146769 |
| 1111.074 | 94.120454 |  | 1111.074 | 81.364706 |  | 1111.074 | 94.208686 |
| 1112.9382 | 94.246366 |  | 1112.9382 | 81.750089 |  | 1112.9382 | 94.471586 |
| 1114.8024 | 94.375497 |  | 1114.8024 | 82.168972 |  | 1114.8024 | 94.883405 |
| 1116.6667 | 94.531644 |  | 1116.6667 | 82.570049 |  | 1116.6667 | 95.181367 |
| 1118.5309 | 94.666227 |  | 1118.5309 | 82.913803 |  | 1118.5309 | 95.349383 |
| 1120.3951 | 94.774246 |  | 1120.3951 | 83.24504 |  | 1120.3951 | 95.575004 |
| 1122.2593 | 94.889247 |  | 1122.2593 | 83.594216 |  | 1122.2593 | 95.908295 |
| 1124.1235 | 94.999371 |  | 1124.1235 | 83.948108 |  | 1124.1235 | 96.206006 |
| 1125.9878 | 95.123319 |  | 1125.9878 | 84.301037 |  | 1125.9878 | 96.376842 |
| 1127.852 | 95.254033 |  | 1127.852 | 84.630379 |  | 1127.852 | 96.423751 |
| 1129.7162 | 95.36188 |  | 1129.7162 | 84.936921 |  | 1129.7162 | 96.426177 |
| 1131.5804 | 95.469426 |  | 1131.5804 | 85.230754 |  | 1131.5804 | 96.413459 |
| 1133.4446 | 95.598785 |  | 1133.4446 | 85.526362 |  | 1133.4446 | 96.403279 |
| 1135.3088 | 95.688857 |  | 1135.3088 | 85.766782 |  | 1135.3088 | 96.317792 |
| 1137.1731 | 95.759875 |  | 1137.1731 | 85.962552 |  | 1137.1731 | 96.152405 |
| 1139.0373 | 95.856103 |  | 1139.0373 | 86.131408 |  | 1139.0373 | 95.928817 |
| 1140.9015 | 95.932892 |  | 1140.9015 | 86.238883 |  | 1140.9015 | 95.695454 |
| 1142.7657 | 95.955448 |  | 1142.7657 | 86.311234 |  | 1142.7657 | 95.458986 |
| 1144.6299 | 95.995163 |  | 1144.6299 | 86.399486 |  | 1144.6299 | 95.310901 |
| 1146.4942 | 96.000449 |  | 1146.4942 | 86.464007 |  | 1146.4942 | 95.271122 |
| 1148.3584 | 95.977923 |  | 1148.3584 | 86.524633 |  | 1148.3584 | 95.362884 |
| 1150.2226 | 95.961387 |  | 1150.2226 | 86.604792 |  | 1150.2226 | 95.582194 |
| 1152.0868 | 95.989365 |  | 1152.0868 | 86.691288 |  | 1152.0868 | 95.898607 |
| 1153.951 | 96.01309 |  | 1153.951 | 86.771319 |  | 1153.951 | 96.224655 |
| 1155.8152 | 95.99271 |  | 1155.8152 | 86.84586 |  | 1155.8152 | 96.537343 |
| 1157.6795 | 95.985437 |  | 1157.6795 | 86.941285 |  | 1157.6795 | 96.782523 |
| 1159.5437 | 96.013729 |  | 1159.5437 | 87.054743 |  | 1159.5437 | 96.999764 |
| 1161.4079 | 96.059582 |  | 1161.4079 | 87.17995 |  | 1161.4079 | 97.170078 |
| 1163.2721 | 96.120369 |  | 1163.2721 | 87.355382 |  | 1163.2721 | 97.305665 |
| 1165.1363 | 96.180575 |  | 1165.1363 | 87.567232 |  | 1165.1363 | 97.387453 |
| 1167.0006 | 96.272365 |  | 1167.0006 | 87.817945 |  | 1167.0006 | 97.464545 |
| 1168.8648 | 96.383119 |  | 1168.8648 | 88.091687 |  | 1168.8648 | 97.546072 |
| 1170.729 | 96.497938 |  | 1170.729 | 88.37034 |  | 1170.729 | 97.630006 |
| 1172.5932 | 96.601653 |  | 1172.5932 | 88.632918 |  | 1172.5932 | 97.689467 |
| 1174.4574 | 96.699396 |  | 1174.4574 | 88.866732 |  | 1174.4574 | 97.750849 |
| 1176.3216 | 96.782329 |  | 1176.3216 | 89.078252 |  | 1176.3216 | 97.782738 |
| 1178.1859 | 96.868292 |  | 1178.1859 | 89.292062 |  | 1178.1859 | 97.80367 |
| 1180.0501 | 96.940562 |  | 1180.0501 | 89.464855 |  | 1180.0501 | 97.818179 |
| 1181.9143 | 97.009334 |  | 1181.9143 | 89.61656 |  | 1181.9143 | 97.857841 |
| 1183.7785 | 97.061429 |  | 1183.7785 | 89.756238 |  | 1183.7785 | 97.841796 |
| 1185.6427 | 97.08971 |  | 1185.6427 | 89.874246 |  | 1185.6427 | 97.819975 |
| 1187.507 | 97.100773 |  | 1187.507 | 89.958554 |  | 1187.507 | 97.800969 |
| 1189.3712 | 97.137012 |  | 1189.3712 | 90.037021 |  | 1189.3712 | 97.776991 |
| 1191.2354 | 97.161821 |  | 1191.2354 | 90.111778 |  | 1191.2354 | 97.757397 |
| 1193.0996 | 97.172483 |  | 1193.0996 | 90.170635 |  | 1193.0996 | 97.712716 |
| 1194.9638 | 97.171639 |  | 1194.9638 | 90.203269 |  | 1194.9638 | 97.64459 |
| 1196.828 | 97.178892 |  | 1196.828 | 90.232685 |  | 1196.828 | 97.575448 |
| 1198.6923 | 97.195633 |  | 1198.6923 | 90.269111 |  | 1198.6923 | 97.466586 |
| 1200.5565 | 97.207287 |  | 1200.5565 | 90.309703 |  | 1200.5565 | 97.330968 |
| 1202.4207 | 97.225035 |  | 1202.4207 | 90.384585 |  | 1202.4207 | 97.213288 |
| 1204.2849 | 97.254181 |  | 1204.2849 | 90.482741 |  | 1204.2849 | 97.225395 |
| 1206.1491 | 97.266053 |  | 1206.1491 | 90.590388 |  | 1206.1491 | 97.384098 |
| 1208.0134 | 97.287217 |  | 1208.0134 | 90.719167 |  | 1208.0134 | 97.571613 |
| 1209.8776 | 97.297528 |  | 1209.8776 | 90.827046 |  | 1209.8776 | 97.695323 |
| 1211.7418 | 97.3298 |  | 1211.7418 | 90.931561 |  | 1211.7418 | 97.767194 |
| 1213.606 | 97.360308 |  | 1213.606 | 91.016242 |  | 1213.606 | 97.730434 |
| 1215.4702 | 97.398831 |  | 1215.4702 | 91.091756 |  | 1215.4702 | 97.653983 |
| 1217.3344 | 97.42082 |  | 1217.3344 | 91.161014 |  | 1217.3344 | 97.563873 |
| 1219.1987 | 97.431407 |  | 1219.1987 | 91.217345 |  | 1219.1987 | 97.415621 |
| 1221.0629 | 97.457444 |  | 1221.0629 | 91.274717 |  | 1221.0629 | 97.213985 |
| 1222.9271 | 97.483917 |  | 1222.9271 | 91.348619 |  | 1222.9271 | 97.060996 |
| 1224.7913 | 97.48335 |  | 1224.7913 | 91.427365 |  | 1224.7913 | 97.03291 |
| 1226.6555 | 97.466026 |  | 1226.6555 | 91.481179 |  | 1226.6555 | 97.162735 |
| 1228.5198 | 97.459871 |  | 1228.5198 | 91.528512 |  | 1228.5198 | 97.388476 |
| 1230.384 | 97.471628 |  | 1230.384 | 91.623994 |  | 1230.384 | 97.617321 |
| 1232.2482 | 97.4689 |  | 1232.2482 | 91.744733 |  | 1232.2482 | 97.74989 |
| 1234.1124 | 97.485922 |  | 1234.1124 | 91.856305 |  | 1234.1124 | 97.848508 |
| 1235.9766 | 97.511059 |  | 1235.9766 | 91.968828 |  | 1235.9766 | 97.915851 |
| 1237.8408 | 97.546518 |  | 1237.8408 | 92.106692 |  | 1237.8408 | 97.954856 |
| 1239.7051 | 97.562448 |  | 1239.7051 | 92.220759 |  | 1239.7051 | 97.961513 |
| 1241.5693 | 97.594921 |  | 1241.5693 | 92.327569 |  | 1241.5693 | 97.97968 |
| 1243.4335 | 97.634592 |  | 1243.4335 | 92.465481 |  | 1243.4335 | 97.992483 |
| 1245.2977 | 97.658275 |  | 1245.2977 | 92.616722 |  | 1245.2977 | 97.991708 |
| 1247.1619 | 97.68012 |  | 1247.1619 | 92.758606 |  | 1247.1619 | 97.991007 |
| 1249.0262 | 97.734547 |  | 1249.0262 | 92.913274 |  | 1249.0262 | 98.006577 |
| 1250.8904 | 97.804362 |  | 1250.8904 | 93.078498 |  | 1250.8904 | 97.991107 |
| 1252.7546 | 97.854153 |  | 1252.7546 | 93.221224 |  | 1252.7546 | 97.996643 |
| 1254.6188 | 97.855695 |  | 1254.6188 | 93.330082 |  | 1254.6188 | 97.989706 |
| 1256.483 | 97.876066 |  | 1256.483 | 93.43358 |  | 1256.483 | 97.990701 |
| 1258.3472 | 97.917584 |  | 1258.3472 | 93.539832 |  | 1258.3472 | 97.988834 |
| 1260.2115 | 97.951745 |  | 1260.2115 | 93.646207 |  | 1260.2115 | 97.978148 |
| 1262.0757 | 97.956279 |  | 1262.0757 | 93.723652 |  | 1262.0757 | 97.880003 |
| 1263.9399 | 97.988257 |  | 1263.9399 | 93.788244 |  | 1263.9399 | 97.857576 |
| 1265.8041 | 98.030029 |  | 1265.8041 | 93.856915 |  | 1265.8041 | 97.871574 |
| 1267.6683 | 98.075893 |  | 1267.6683 | 93.949534 |  | 1267.6683 | 97.842682 |
| 1269.5326 | 98.091487 |  | 1269.5326 | 94.023822 |  | 1269.5326 | 97.804067 |
| 1271.3968 | 98.071045 |  | 1271.3968 | 94.063771 |  | 1271.3968 | 97.762769 |
| 1273.261 | 98.089213 |  | 1273.261 | 94.109115 |  | 1273.261 | 97.706754 |
| 1275.1252 | 98.163928 |  | 1275.1252 | 94.175582 |  | 1275.1252 | 97.742797 |
| 1276.9894 | 98.189749 |  | 1276.9894 | 94.212051 |  | 1276.9894 | 97.814818 |
| 1278.8536 | 98.188459 |  | 1278.8536 | 94.231196 |  | 1278.8536 | 97.836953 |
| 1280.7179 | 98.208981 |  | 1280.7179 | 94.256979 |  | 1280.7179 | 97.808959 |
| 1282.5821 | 98.202235 |  | 1282.5821 | 94.237039 |  | 1282.5821 | 97.768672 |
| 1284.4463 | 98.199739 |  | 1284.4463 | 94.237868 |  | 1284.4463 | 97.796606 |
| 1286.3105 | 98.223776 |  | 1286.3105 | 94.29345 |  | 1286.3105 | 97.865147 |
| 1288.1747 | 98.220606 |  | 1288.1747 | 94.30293 |  | 1288.1747 | 97.838847 |
| 1290.039 | 98.173711 |  | 1290.039 | 94.263137 |  | 1290.039 | 97.738029 |
| 1291.9032 | 98.142143 |  | 1291.9032 | 94.217876 |  | 1291.9032 | 97.707621 |
| 1293.7674 | 98.17042 |  | 1293.7674 | 94.192318 |  | 1293.7674 | 97.724228 |
| 1295.6316 | 98.203688 |  | 1295.6316 | 94.157868 |  | 1295.6316 | 97.70474 |
| 1297.4958 | 98.178199 |  | 1297.4958 | 94.097529 |  | 1297.4958 | 97.696946 |
| 1299.36 | 98.133188 |  | 1299.36 | 94.022662 |  | 1299.36 | 97.748841 |
| 1301.2243 | 98.131637 |  | 1301.2243 | 93.951759 |  | 1301.2243 | 97.8637 |
| 1303.0885 | 98.102901 |  | 1303.0885 | 93.841798 |  | 1303.0885 | 97.911962 |
| 1304.9527 | 98.043964 |  | 1304.9527 | 93.717579 |  | 1304.9527 | 97.924944 |
| 1306.8169 | 97.994693 |  | 1306.8169 | 93.589872 |  | 1306.8169 | 97.938504 |
| 1308.6811 | 97.9427 |  | 1308.6811 | 93.422874 |  | 1308.6811 | 97.925935 |
| 1310.5454 | 97.898789 |  | 1310.5454 | 93.248903 |  | 1310.5454 | 97.910295 |
| 1312.4096 | 97.864871 |  | 1312.4096 | 93.108511 |  | 1312.4096 | 97.932239 |
| 1314.2738 | 97.731103 |  | 1314.2738 | 92.915329 |  | 1314.2738 | 97.756897 |
| 1316.138 | 97.668239 |  | 1316.138 | 92.777027 |  | 1316.138 | 97.651237 |
| 1318.0022 | 97.693863 |  | 1318.0022 | 92.737685 |  | 1318.0022 | 97.697554 |
| 1319.8664 | 97.600997 |  | 1319.8664 | 92.676827 |  | 1319.8664 | 97.534256 |
| 1321.7307 | 97.610197 |  | 1321.7307 | 92.738144 |  | 1321.7307 | 97.364593 |
| 1323.5949 | 97.80414 |  | 1323.5949 | 92.921752 |  | 1323.5949 | 97.458131 |
| 1325.4591 | 97.89042 |  | 1325.4591 | 93.054727 |  | 1325.4591 | 97.469563 |
| 1327.3233 | 97.934553 |  | 1327.3233 | 93.193109 |  | 1327.3233 | 97.330889 |
| 1329.1875 | 98.006265 |  | 1329.1875 | 93.305155 |  | 1329.1875 | 97.211064 |
| 1331.0518 | 98.027818 |  | 1331.0518 | 93.356104 |  | 1331.0518 | 97.146062 |
| 1332.916 | 98.038442 |  | 1332.916 | 93.408338 |  | 1332.916 | 97.156593 |
| 1334.7802 | 98.10964 |  | 1334.7802 | 93.500666 |  | 1334.7802 | 97.233498 |
| 1336.6444 | 98.186516 |  | 1336.6444 | 93.616769 |  | 1336.6444 | 97.306346 |
| 1338.5086 | 98.054064 |  | 1338.5086 | 93.606996 |  | 1338.5086 | 97.131975 |
| 1340.3728 | 97.669921 |  | 1340.3728 | 93.385587 |  | 1340.3728 | 96.511642 |
| 1342.2371 | 97.608405 |  | 1342.2371 | 93.255112 |  | 1342.2371 | 96.261075 |
| 1344.1013 | 97.973057 |  | 1344.1013 | 93.401992 |  | 1344.1013 | 96.851417 |
| 1345.9655 | 98.139269 |  | 1345.9655 | 93.493601 |  | 1345.9655 | 97.28079 |
| 1347.8297 | 98.127982 |  | 1347.8297 | 93.476875 |  | 1347.8297 | 97.320325 |
| 1349.6939 | 98.123864 |  | 1349.6939 | 93.448638 |  | 1349.6939 | 97.332237 |
| 1351.5582 | 98.085541 |  | 1351.5582 | 93.383063 |  | 1351.5582 | 97.337141 |
| 1353.4224 | 98.116832 |  | 1353.4224 | 93.358934 |  | 1353.4224 | 97.479164 |
| 1355.2866 | 98.076513 |  | 1355.2866 | 93.284267 |  | 1355.2866 | 97.521324 |
| 1357.1508 | 97.993688 |  | 1357.1508 | 93.162412 |  | 1357.1508 | 97.443259 |
| 1359.015 | 98.060112 |  | 1359.015 | 93.166479 |  | 1359.015 | 97.549311 |
| 1360.8792 | 98.147237 |  | 1360.8792 | 93.210412 |  | 1360.8792 | 97.779212 |
| 1362.7435 | 97.927197 |  | 1362.7435 | 92.988419 |  | 1362.7435 | 97.487601 |
| 1364.6077 | 97.52089 |  | 1364.6077 | 92.555669 |  | 1364.6077 | 96.640128 |
| 1366.4719 | 97.726144 |  | 1366.4719 | 92.592432 |  | 1366.4719 | 96.771724 |
| 1368.3361 | 98.01723 |  | 1368.3361 | 92.749322 |  | 1368.3361 | 97.270298 |
| 1370.2003 | 97.873229 |  | 1370.2003 | 92.612051 |  | 1370.2003 | 97.03557 |
| 1372.0646 | 97.901348 |  | 1372.0646 | 92.621846 |  | 1372.0646 | 97.112555 |
| 1373.9288 | 97.986418 |  | 1373.9288 | 92.682972 |  | 1373.9288 | 97.375935 |
| 1375.793 | 97.600806 |  | 1375.793 | 92.377659 |  | 1375.793 | 96.757411 |
| 1377.6572 | 97.549046 |  | 1377.6572 | 92.271261 |  | 1377.6572 | 96.532091 |
| 1379.5214 | 97.86453 |  | 1379.5214 | 92.416974 |  | 1379.5214 | 96.968825 |
| 1381.3856 | 97.906954 |  | 1381.3856 | 92.405842 |  | 1381.3856 | 97.028331 |
| 1383.2499 | 97.967669 |  | 1383.2499 | 92.432221 |  | 1383.2499 | 97.226682 |
| 1385.1141 | 98.112664 |  | 1385.1141 | 92.530668 |  | 1385.1141 | 97.614027 |
| 1386.9783 | 98.122219 |  | 1386.9783 | 92.570007 |  | 1386.9783 | 97.873922 |
| 1388.8425 | 97.694125 |  | 1388.8425 | 92.25619 |  | 1388.8425 | 97.256143 |
| 1390.7067 | 97.664334 |  | 1390.7067 | 92.173002 |  | 1390.7067 | 97.111709 |
| 1392.571 | 98.124785 |  | 1392.571 | 92.508283 |  | 1392.571 | 97.925608 |
| 1394.4352 | 98.27021 |  | 1394.4352 | 92.644104 |  | 1394.4352 | 98.313465 |
| 1396.2994 | 97.801496 |  | 1396.2994 | 92.30436 |  | 1396.2994 | 97.503582 |
| 1398.1636 | 97.579759 |  | 1398.1636 | 92.068238 |  | 1398.1636 | 96.951341 |
| 1400.0278 | 97.69657 |  | 1400.0278 | 91.995438 |  | 1400.0278 | 96.9681 |
| 1401.892 | 97.662317 |  | 1401.892 | 91.863472 |  | 1401.892 | 96.807421 |
| 1403.7563 | 98.015681 |  | 1403.7563 | 92.068456 |  | 1403.7563 | 97.445953 |
| 1405.6205 | 98.054295 |  | 1405.6205 | 92.025647 |  | 1405.6205 | 97.497628 |
| 1407.4847 | 97.753101 |  | 1407.4847 | 91.741495 |  | 1407.4847 | 96.895104 |
| 1409.3489 | 97.927441 |  | 1409.3489 | 91.835147 |  | 1409.3489 | 97.191527 |
| 1411.2131 | 98.03764 |  | 1411.2131 | 91.887754 |  | 1411.2131 | 97.440332 |
| 1413.0774 | 97.940898 |  | 1413.0774 | 91.777793 |  | 1413.0774 | 97.28814 |
| 1414.9416 | 98.034603 |  | 1414.9416 | 91.848431 |  | 1414.9416 | 97.438568 |
| 1416.8058 | 98.228144 |  | 1416.8058 | 92.07736 |  | 1416.8058 | 97.892749 |
| 1418.67 | 98.112102 |  | 1418.67 | 92.101676 |  | 1418.67 | 97.848906 |
| 1420.5342 | 97.369616 |  | 1420.5342 | 91.554913 |  | 1420.5342 | 96.371122 |
| 1422.3984 | 97.365933 |  | 1422.3984 | 91.499465 |  | 1422.3984 | 96.127713 |
| 1424.2627 | 97.846295 |  | 1424.2627 | 91.836477 |  | 1424.2627 | 96.95279 |
| 1426.1269 | 97.753674 |  | 1426.1269 | 91.783398 |  | 1426.1269 | 96.769913 |
| 1427.9911 | 97.91925 |  | 1427.9911 | 91.942729 |  | 1427.9911 | 97.014301 |
| 1429.8553 | 98.192365 |  | 1429.8553 | 92.206502 |  | 1429.8553 | 97.487989 |
| 1431.7195 | 97.928179 |  | 1431.7195 | 92.076802 |  | 1431.7195 | 96.983126 |
| 1433.5838 | 97.953247 |  | 1433.5838 | 92.16381 |  | 1433.5838 | 96.990123 |
| 1435.448 | 98.297243 |  | 1435.448 | 92.521087 |  | 1435.448 | 97.708913 |
| 1437.3122 | 98.060402 |  | 1437.3122 | 92.425813 |  | 1437.3122 | 97.346691 |
| 1439.1764 | 97.384178 |  | 1439.1764 | 91.934044 |  | 1439.1764 | 95.867962 |
| 1441.0406 | 97.818716 |  | 1441.0406 | 92.196392 |  | 1441.0406 | 96.456977 |
| 1442.9048 | 98.252434 |  | 1442.9048 | 92.492132 |  | 1442.9048 | 97.184583 |
| 1444.7691 | 98.259155 |  | 1444.7691 | 92.544775 |  | 1444.7691 | 97.296198 |
| 1446.6333 | 98.290275 |  | 1446.6333 | 92.611991 |  | 1446.6333 | 97.5597 |
| 1448.4975 | 98.131281 |  | 1448.4975 | 92.508269 |  | 1448.4975 | 97.351202 |
| 1450.3617 | 97.876513 |  | 1450.3617 | 92.343643 |  | 1450.3617 | 96.829476 |
| 1452.2259 | 98.058282 |  | 1452.2259 | 92.507039 |  | 1452.2259 | 97.140997 |
| 1454.0902 | 98.330136 |  | 1454.0902 | 92.773778 |  | 1454.0902 | 97.782631 |
| 1455.9544 | 98.576539 |  | 1455.9544 | 93.121865 |  | 1455.9544 | 98.39955 |
| 1457.8186 | 98.077709 |  | 1457.8186 | 93.000114 |  | 1457.8186 | 97.379648 |
| 1459.6828 | 96.53411 |  | 1459.6828 | 91.845474 |  | 1459.6828 | 94.367789 |
| 1461.547 | 97.27038 |  | 1461.547 | 92.199117 |  | 1461.547 | 95.652947 |
| 1463.4112 | 98.1824 |  | 1463.4112 | 92.793282 |  | 1463.4112 | 97.402956 |
| 1465.2755 | 98.346836 |  | 1465.2755 | 92.984357 |  | 1465.2755 | 97.923208 |
| 1467.1397 | 97.799182 |  | 1467.1397 | 92.750914 |  | 1467.1397 | 97.041508 |
| 1469.0039 | 98.05808 |  | 1469.0039 | 93.032289 |  | 1469.0039 | 97.503048 |
| 1470.8681 | 98.668355 |  | 1470.8681 | 93.631071 |  | 1470.8681 | 98.777131 |
| 1472.7323 | 98.676266 |  | 1472.7323 | 93.9163 |  | 1472.7323 | 99.103107 |
| 1474.5966 | 97.57197 |  | 1474.5966 | 93.454592 |  | 1474.5966 | 97.090651 |
| 1476.4608 | 97.507485 |  | 1476.4608 | 93.454359 |  | 1476.4608 | 96.682031 |
| 1478.325 | 97.978795 |  | 1478.325 | 93.729572 |  | 1478.325 | 97.315563 |
| 1480.1892 | 98.325743 |  | 1480.1892 | 93.951902 |  | 1480.1892 | 97.935896 |
| 1482.0534 | 98.515775 |  | 1482.0534 | 94.124488 |  | 1482.0534 | 98.37685 |
| 1483.9176 | 98.422643 |  | 1483.9176 | 94.134287 |  | 1483.9176 | 98.310814 |
| 1485.7819 | 98.622483 |  | 1485.7819 | 94.304906 |  | 1485.7819 | 98.779753 |
| 1487.6461 | 98.80719 |  | 1487.6461 | 94.488901 |  | 1487.6461 | 99.242599 |
| 1489.5103 | 98.428822 |  | 1489.5103 | 94.325647 |  | 1489.5103 | 98.643624 |
| 1491.3745 | 97.611051 |  | 1491.3745 | 93.712188 |  | 1491.3745 | 97.118735 |
| 1493.2387 | 97.65687 |  | 1493.2387 | 93.621459 |  | 1493.2387 | 97.064498 |
| 1495.1029 | 98.444614 |  | 1495.1029 | 94.111608 |  | 1495.1029 | 98.551618 |
| 1496.9672 | 98.631429 |  | 1496.9672 | 94.225225 |  | 1496.9672 | 99.174239 |
| 1498.8314 | 97.823044 |  | 1498.8314 | 93.623933 |  | 1498.8314 | 97.804921 |
| 1500.6956 | 97.67177 |  | 1500.6956 | 93.407237 |  | 1500.6956 | 97.3866 |
| 1502.5598 | 98.016324 |  | 1502.5598 | 93.627536 |  | 1502.5598 | 97.99033 |
| 1504.424 | 98.587723 |  | 1504.424 | 94.087145 |  | 1504.424 | 99.237512 |
| 1506.2883 | 99.157955 |  | 1506.2883 | 94.620227 |  | 1506.2883 | 100.79694 |
| 1508.1525 | 97.338851 |  | 1508.1525 | 93.677869 |  | 1508.1525 | 97.848116 |
| 1510.0167 | 95.784453 |  | 1510.0167 | 92.38352 |  | 1510.0167 | 94.784505 |
| 1511.8809 | 97.023819 |  | 1511.8809 | 92.961649 |  | 1511.8809 | 96.627165 |
| 1513.7451 | 97.733369 |  | 1513.7451 | 93.344336 |  | 1513.7451 | 97.807765 |
| 1515.6093 | 98.102558 |  | 1515.6093 | 93.628496 |  | 1515.6093 | 98.593536 |
| 1517.4736 | 97.9873 |  | 1517.4736 | 93.692471 |  | 1517.4736 | 98.607623 |
| 1519.3378 | 97.494444 |  | 1519.3378 | 93.528989 |  | 1519.3378 | 97.841549 |
| 1521.202 | 97.725318 |  | 1521.202 | 93.703263 |  | 1521.202 | 98.455039 |
| 1523.0662 | 97.177452 |  | 1523.0662 | 93.288457 |  | 1523.0662 | 97.46609 |
| 1524.9304 | 96.931967 |  | 1524.9304 | 93.064141 |  | 1524.9304 | 96.690288 |
| 1526.7947 | 97.449466 |  | 1526.7947 | 93.323375 |  | 1526.7947 | 97.580392 |
| 1528.6589 | 97.309831 |  | 1528.6589 | 93.169577 |  | 1528.6589 | 97.36623 |
| 1530.5231 | 97.515414 |  | 1530.5231 | 93.298148 |  | 1530.5231 | 97.66041 |
| 1532.3873 | 98.115443 |  | 1532.3873 | 93.697402 |  | 1532.3873 | 98.792338 |
| 1534.2515 | 97.931332 |  | 1534.2515 | 93.531414 |  | 1534.2515 | 98.626364 |
| 1536.1157 | 97.221638 |  | 1536.1157 | 93.015342 |  | 1536.1157 | 97.34467 |
| 1537.98 | 97.979965 |  | 1537.98 | 93.554403 |  | 1537.98 | 98.776522 |
| 1539.8442 | 98.793019 |  | 1539.8442 | 94.208484 |  | 1539.8442 | 100.68752 |
| 1541.7084 | 96.851146 |  | 1541.7084 | 93.233938 |  | 1541.7084 | 97.461637 |
| 1543.5726 | 95.949045 |  | 1543.5726 | 92.563147 |  | 1543.5726 | 95.579753 |
| 1545.4368 | 96.619283 |  | 1545.4368 | 92.788933 |  | 1545.4368 | 96.330068 |
| 1547.3011 | 97.064344 |  | 1547.3011 | 92.974313 |  | 1547.3011 | 96.877031 |
| 1549.1653 | 97.742736 |  | 1549.1653 | 93.401205 |  | 1549.1653 | 98.070751 |
| 1551.0295 | 97.872122 |  | 1551.0295 | 93.546622 |  | 1551.0295 | 98.449567 |
| 1552.8937 | 97.643677 |  | 1552.8937 | 93.525306 |  | 1552.8937 | 98.25061 |
| 1554.7579 | 97.903487 |  | 1554.7579 | 93.776771 |  | 1554.7579 | 98.884211 |
| 1556.6221 | 97.977449 |  | 1556.6221 | 93.972641 |  | 1556.6221 | 99.194894 |
| 1558.4864 | 98.280496 |  | 1558.4864 | 94.331185 |  | 1558.4864 | 99.981046 |
| 1560.3506 | 96.69833 |  | 1560.3506 | 93.224978 |  | 1560.3506 | 96.856562 |
| 1562.2148 | 95.799801 |  | 1562.2148 | 92.332267 |  | 1562.2148 | 94.830098 |
| 1564.079 | 97.277454 |  | 1564.079 | 93.206207 |  | 1564.079 | 97.301291 |
| 1565.9432 | 97.856804 |  | 1565.9432 | 93.552321 |  | 1565.9432 | 98.400248 |
| 1567.8075 | 97.913718 |  | 1567.8075 | 93.624657 |  | 1567.8075 | 98.585238 |
| 1569.6717 | 98.047635 |  | 1569.6717 | 93.72265 |  | 1569.6717 | 98.907373 |
| 1571.5359 | 97.421839 |  | 1571.5359 | 93.221396 |  | 1571.5359 | 97.695528 |
| 1573.4001 | 97.4623 |  | 1573.4001 | 93.189593 |  | 1573.4001 | 97.697008 |
| 1575.2643 | 98.205872 |  | 1575.2643 | 93.703637 |  | 1575.2643 | 99.235688 |
| 1577.1285 | 97.837184 |  | 1577.1285 | 93.496921 |  | 1577.1285 | 98.74306 |
| 1578.9928 | 96.848202 |  | 1578.9928 | 92.765061 |  | 1578.9928 | 96.727834 |
| 1580.857 | 97.40047 |  | 1580.857 | 93.019609 |  | 1580.857 | 97.487283 |
| 1582.7212 | 97.84099 |  | 1582.7212 | 93.278618 |  | 1582.7212 | 98.331688 |
| 1584.5854 | 97.818532 |  | 1584.5854 | 93.270211 |  | 1584.5854 | 98.376664 |
| 1586.4496 | 97.821548 |  | 1586.4496 | 93.260294 |  | 1586.4496 | 98.38933 |
| 1588.3139 | 97.824968 |  | 1588.3139 | 93.254455 |  | 1588.3139 | 98.427815 |
| 1590.1781 | 97.777353 |  | 1590.1781 | 93.213648 |  | 1590.1781 | 98.419486 |
| 1592.0423 | 97.704636 |  | 1592.0423 | 93.14505 |  | 1592.0423 | 98.358731 |
| 1593.9065 | 97.664111 |  | 1593.9065 | 93.079377 |  | 1593.9065 | 98.339291 |
| 1595.7707 | 97.570226 |  | 1595.7707 | 92.971195 |  | 1595.7707 | 98.252126 |
| 1597.6349 | 97.49324 |  | 1597.6349 | 92.866037 |  | 1597.6349 | 98.117037 |
| 1599.4992 | 97.57824 |  | 1599.4992 | 92.828415 |  | 1599.4992 | 98.231945 |
| 1601.3634 | 97.616778 |  | 1601.3634 | 92.784006 |  | 1601.3634 | 98.42594 |
| 1603.2276 | 97.512574 |  | 1603.2276 | 92.699599 |  | 1603.2276 | 98.370109 |
| 1605.0918 | 97.415112 |  | 1605.0918 | 92.611516 |  | 1605.0918 | 98.237143 |
| 1606.956 | 97.468366 |  | 1606.956 | 92.617225 |  | 1606.956 | 98.369491 |
| 1608.8203 | 97.47217 |  | 1608.8203 | 92.589239 |  | 1608.8203 | 98.415529 |
| 1610.6845 | 97.323754 |  | 1610.6845 | 92.436041 |  | 1610.6845 | 98.149263 |
| 1612.5487 | 97.335485 |  | 1612.5487 | 92.418018 |  | 1612.5487 | 98.123697 |
| 1614.4129 | 97.630391 |  | 1614.4129 | 92.668691 |  | 1614.4129 | 98.725204 |
| 1616.2771 | 97.918391 |  | 1616.2771 | 92.951066 |  | 1616.2771 | 99.461195 |
| 1618.1413 | 97.182787 |  | 1618.1413 | 92.502262 |  | 1618.1413 | 98.163464 |
| 1620.0056 | 96.643319 |  | 1620.0056 | 92.035953 |  | 1620.0056 | 96.992619 |
| 1621.8698 | 97.325855 |  | 1621.8698 | 92.446595 |  | 1621.8698 | 98.232283 |
| 1623.734 | 97.498395 |  | 1623.734 | 92.597624 |  | 1623.734 | 98.737073 |
| 1625.5982 | 96.891049 |  | 1625.5982 | 92.132453 |  | 1625.5982 | 97.551666 |
| 1627.4624 | 97.061241 |  | 1627.4624 | 92.173699 |  | 1627.4624 | 97.702724 |
| 1629.3267 | 97.211476 |  | 1629.3267 | 92.265189 |  | 1629.3267 | 97.870825 |
| 1631.1909 | 97.144877 |  | 1631.1909 | 92.225841 |  | 1631.1909 | 97.693587 |
| 1633.0551 | 97.52655 |  | 1633.0551 | 92.561397 |  | 1633.0551 | 98.509818 |
| 1634.9193 | 98.029143 |  | 1634.9193 | 93.032506 |  | 1634.9193 | 99.683401 |
| 1636.7835 | 97.330617 |  | 1636.7835 | 92.612844 |  | 1636.7835 | 98.545098 |
| 1638.6477 | 96.03869 |  | 1638.6477 | 91.648579 |  | 1638.6477 | 96.03871 |
| 1640.512 | 96.689538 |  | 1640.512 | 92.008623 |  | 1640.512 | 96.918224 |
| 1642.3762 | 97.286796 |  | 1642.3762 | 92.404075 |  | 1642.3762 | 98.005604 |
| 1644.2404 | 97.589033 |  | 1644.2404 | 92.702228 |  | 1644.2404 | 98.720798 |
| 1646.1046 | 97.9726 |  | 1646.1046 | 93.112059 |  | 1646.1046 | 99.583922 |
| 1647.9688 | 97.290764 |  | 1647.9688 | 92.851074 |  | 1647.9688 | 98.365257 |
| 1649.8331 | 96.274752 |  | 1649.8331 | 92.234395 |  | 1649.8331 | 96.498448 |
| 1651.6973 | 97.363629 |  | 1651.6973 | 92.91963 |  | 1651.6973 | 98.555304 |
| 1653.5615 | 98.213513 |  | 1653.5615 | 93.539618 |  | 1653.5615 | 100.24606 |
| 1655.4257 | 95.744399 |  | 1655.4257 | 92.136642 |  | 1655.4257 | 95.194289 |
| 1657.2899 | 96.070401 |  | 1657.2899 | 92.318082 |  | 1657.2899 | 95.587464 |
| 1659.1541 | 97.222723 |  | 1659.1541 | 93.07319 |  | 1659.1541 | 97.594466 |
| 1661.0184 | 97.78709 |  | 1661.0184 | 93.57858 |  | 1661.0184 | 98.679325 |
| 1662.8826 | 97.892042 |  | 1662.8826 | 93.854158 |  | 1662.8826 | 98.836375 |
| 1664.7468 | 97.425432 |  | 1664.7468 | 93.7585 |  | 1664.7468 | 97.742513 |
| 1666.611 | 97.565229 |  | 1666.611 | 93.962523 |  | 1666.611 | 97.909033 |
| 1668.4752 | 98.221651 |  | 1668.4752 | 94.492167 |  | 1668.4752 | 99.275077 |
| 1670.3395 | 98.024508 |  | 1670.3395 | 94.637898 |  | 1670.3395 | 99.086083 |
| 1672.2037 | 97.149307 |  | 1672.2037 | 94.318306 |  | 1672.2037 | 97.263121 |
| 1674.0679 | 97.681653 |  | 1674.0679 | 94.676472 |  | 1674.0679 | 97.814467 |
| 1675.9321 | 98.104336 |  | 1675.9321 | 95.014237 |  | 1675.9321 | 98.428279 |
| 1677.7963 | 97.634614 |  | 1677.7963 | 94.876672 |  | 1677.7963 | 97.559964 |
| 1679.6605 | 97.981312 |  | 1679.6605 | 95.2494 |  | 1679.6605 | 98.152086 |
| 1681.5248 | 98.333106 |  | 1681.5248 | 95.674452 |  | 1681.5248 | 98.910115 |
| 1683.389 | 98.566856 |  | 1683.389 | 96.043713 |  | 1683.389 | 99.6866 |
| 1685.2532 | 98.117578 |  | 1685.2532 | 96.013108 |  | 1685.2532 | 98.628544 |
| 1687.1174 | 96.980707 |  | 1687.1174 | 95.391171 |  | 1687.1174 | 95.909881 |
| 1688.9816 | 97.795446 |  | 1688.9816 | 95.78168 |  | 1688.9816 | 97.248651 |
| 1690.8459 | 98.230557 |  | 1690.8459 | 96.096527 |  | 1690.8459 | 98.107363 |
| 1692.7101 | 98.264957 |  | 1692.7101 | 96.262321 |  | 1692.7101 | 98.22806 |
| 1694.5743 | 98.83895 |  | 1694.5743 | 96.733986 |  | 1694.5743 | 99.418186 |
| 1696.4385 | 99.206051 |  | 1696.4385 | 97.147699 |  | 1696.4385 | 100.10418 |
| 1698.3027 | 98.244987 |  | 1698.3027 | 96.851123 |  | 1698.3027 | 98.228756 |
| 1700.1669 | 98.420278 |  | 1700.1669 | 97.02106 |  | 1700.1669 | 98.489992 |
| 1702.0312 | 97.902161 |  | 1702.0312 | 96.74735 |  | 1702.0312 | 97.197098 |
| 1703.8954 | 97.661237 |  | 1703.8954 | 96.596214 |  | 1703.8954 | 96.780842 |
| 1705.7596 | 98.420922 |  | 1705.7596 | 97.040082 |  | 1705.7596 | 98.201434 |
| 1707.6238 | 98.292319 |  | 1707.6238 | 97.02667 |  | 1707.6238 | 97.905079 |
| 1709.488 | 98.255358 |  | 1709.488 | 97.043335 |  | 1709.488 | 97.765384 |
| 1711.3523 | 98.714704 |  | 1711.3523 | 97.338115 |  | 1711.3523 | 98.474165 |
| 1713.2165 | 99.006732 |  | 1713.2165 | 97.552358 |  | 1713.2165 | 99.079851 |
| 1715.0807 | 99.15045 |  | 1715.0807 | 97.732712 |  | 1715.0807 | 99.655023 |
| 1716.9449 | 98.843511 |  | 1716.9449 | 97.666716 |  | 1716.9449 | 99.35302 |
| 1718.8091 | 98.060358 |  | 1718.8091 | 97.221469 |  | 1718.8091 | 97.493199 |
| 1720.6733 | 97.630511 |  | 1720.6733 | 96.885295 |  | 1720.6733 | 96.328966 |
| 1722.5376 | 98.378279 |  | 1722.5376 | 97.212962 |  | 1722.5376 | 97.712384 |
| 1724.4018 | 98.84869 |  | 1724.4018 | 97.443636 |  | 1724.4018 | 98.611077 |
| 1726.266 | 98.83868 |  | 1726.266 | 97.485252 |  | 1726.266 | 98.553405 |
| 1728.1302 | 99.001316 |  | 1728.1302 | 97.622555 |  | 1728.1302 | 98.862317 |
| 1729.9944 | 99.232398 |  | 1729.9944 | 97.821094 |  | 1729.9944 | 99.352841 |
| 1731.8587 | 99.055161 |  | 1731.8587 | 97.81414 |  | 1731.8587 | 99.22785 |
| 1733.7229 | 99.118002 |  | 1733.7229 | 97.906866 |  | 1733.7229 | 99.58126 |
| 1735.5871 | 98.390882 |  | 1735.5871 | 97.486045 |  | 1735.5871 | 97.855508 |
| 1737.4513 | 97.863383 |  | 1737.4513 | 97.142137 |  | 1737.4513 | 96.784318 |
| 1739.3155 | 98.814983 |  | 1739.3155 | 97.669109 |  | 1739.3155 | 98.543949 |
| 1741.1797 | 98.97546 |  | 1741.1797 | 97.765966 |  | 1741.1797 | 98.815897 |
| 1743.044 | 98.706695 |  | 1743.044 | 97.656484 |  | 1743.044 | 98.320488 |
| 1744.9082 | 98.794019 |  | 1744.9082 | 97.779344 |  | 1744.9082 | 98.485819 |
| 1746.7724 | 98.838157 |  | 1746.7724 | 97.830047 |  | 1746.7724 | 98.628706 |
| 1748.6366 | 98.916115 |  | 1748.6366 | 97.932416 |  | 1748.6366 | 98.865753 |
| 1750.5008 | 98.704681 |  | 1750.5008 | 97.926115 |  | 1750.5008 | 98.458993 |
| 1752.3651 | 98.32406 |  | 1752.3651 | 97.738883 |  | 1752.3651 | 97.528226 |
| 1754.2293 | 98.350091 |  | 1754.2293 | 97.664076 |  | 1754.2293 | 97.402719 |
| 1756.0935 | 99.086443 |  | 1756.0935 | 98.039175 |  | 1756.0935 | 98.661995 |
| 1757.9577 | 99.255039 |  | 1757.9577 | 98.150666 |  | 1757.9577 | 98.968153 |
| 1759.8219 | 98.881787 |  | 1759.8219 | 97.940365 |  | 1759.8219 | 98.453276 |
| 1761.6861 | 99.144522 |  | 1761.6861 | 98.09729 |  | 1761.6861 | 98.967942 |
| 1763.5504 | 98.955775 |  | 1763.5504 | 98.063413 |  | 1763.5504 | 98.564159 |
| 1765.4146 | 98.759964 |  | 1765.4146 | 97.970938 |  | 1765.4146 | 98.15973 |
| 1767.2788 | 99.337904 |  | 1767.2788 | 98.243903 |  | 1767.2788 | 99.121336 |
| 1769.143 | 99.44342 |  | 1769.143 | 98.274486 |  | 1769.143 | 99.372722 |
| 1771.0072 | 99.16264 |  | 1771.0072 | 98.169243 |  | 1771.0072 | 99.147158 |
| 1772.8715 | 99.139475 |  | 1772.8715 | 98.22184 |  | 1772.8715 | 99.100814 |
| 1774.7357 | 98.45978 |  | 1774.7357 | 97.887753 |  | 1774.7357 | 97.569107 |
| 1776.5999 | 98.544341 |  | 1776.5999 | 97.901948 |  | 1776.5999 | 97.681674 |
| 1778.4641 | 98.984592 |  | 1778.4641 | 98.049411 |  | 1778.4641 | 98.495023 |
| 1780.3283 | 99.289079 |  | 1780.3283 | 98.205571 |  | 1780.3283 | 99.030855 |
| 1782.1925 | 99.098431 |  | 1782.1925 | 98.19151 |  | 1782.1925 | 98.676094 |
| 1784.0568 | 99.02566 |  | 1784.0568 | 98.155323 |  | 1784.0568 | 98.52758 |
| 1785.921 | 99.194168 |  | 1785.921 | 98.190787 |  | 1785.921 | 98.709361 |
| 1787.7852 | 99.11366 |  | 1787.7852 | 98.124721 |  | 1787.7852 | 98.590139 |
| 1789.6494 | 99.297843 |  | 1789.6494 | 98.263645 |  | 1789.6494 | 99.019138 |
| 1791.5136 | 99.588278 |  | 1791.5136 | 98.462078 |  | 1791.5136 | 99.725903 |
| 1793.3779 | 99.119254 |  | 1793.3779 | 98.235479 |  | 1793.3779 | 98.925822 |
| 1795.2421 | 98.50877 |  | 1795.2421 | 97.907577 |  | 1795.2421 | 97.691808 |
| 1797.1063 | 98.815994 |  | 1797.1063 | 98.031349 |  | 1797.1063 | 98.176173 |
| 1798.9705 | 99.16086 |  | 1798.9705 | 98.167357 |  | 1798.9705 | 98.811749 |
| 1800.8347 | 99.299631 |  | 1800.8347 | 98.225874 |  | 1800.8347 | 99.008567 |
| 1802.6989 | 99.00954 |  | 1802.6989 | 98.076883 |  | 1802.6989 | 98.477588 |
| 1804.5632 | 98.880595 |  | 1804.5632 | 98.028789 |  | 1804.5632 | 98.213056 |
| 1806.4274 | 99.122151 |  | 1806.4274 | 98.183916 |  | 1806.4274 | 98.671931 |
| 1808.2916 | 99.286384 |  | 1808.2916 | 98.277412 |  | 1808.2916 | 99.029795 |
| 1810.1558 | 99.294118 |  | 1810.1558 | 98.287814 |  | 1810.1558 | 99.07174 |
| 1812.02 | 99.256399 |  | 1812.02 | 98.259984 |  | 1812.02 | 98.873073 |
| 1813.8843 | 99.087086 |  | 1813.8843 | 98.168107 |  | 1813.8843 | 98.498948 |
| 1815.7485 | 99.213195 |  | 1815.7485 | 98.252706 |  | 1815.7485 | 98.79448 |
| 1817.6127 | 99.376782 |  | 1817.6127 | 98.337019 |  | 1817.6127 | 99.157669 |
| 1819.4769 | 99.295225 |  | 1819.4769 | 98.267106 |  | 1819.4769 | 99.012012 |
| 1821.3411 | 99.252894 |  | 1821.3411 | 98.244016 |  | 1821.3411 | 98.915179 |
| 1823.2053 | 99.361665 |  | 1823.2053 | 98.318537 |  | 1823.2053 | 99.146356 |
| 1825.0696 | 99.442184 |  | 1825.0696 | 98.37841 |  | 1825.0696 | 99.41301 |
| 1826.9338 | 99.168424 |  | 1826.9338 | 98.2378 |  | 1826.9338 | 98.823949 |
| 1828.798 | 99.199166 |  | 1828.798 | 98.25758 |  | 1828.798 | 98.769598 |
| 1830.6622 | 99.355864 |  | 1830.6622 | 98.345982 |  | 1830.6622 | 99.047869 |
| 1832.5264 | 98.774916 |  | 1832.5264 | 97.996573 |  | 1832.5264 | 97.977954 |
| 1834.3907 | 98.958329 |  | 1834.3907 | 98.085599 |  | 1834.3907 | 98.299307 |
| 1836.2549 | 99.303211 |  | 1836.2549 | 98.317184 |  | 1836.2549 | 98.949524 |
| 1838.1191 | 99.329212 |  | 1838.1191 | 98.321331 |  | 1838.1191 | 99.044539 |
| 1839.9833 | 99.221992 |  | 1839.9833 | 98.222804 |  | 1839.9833 | 98.788688 |
| 1841.8475 | 99.371541 |  | 1841.8475 | 98.30861 |  | 1841.8475 | 99.083744 |
| 1843.7117 | 99.589126 |  | 1843.7117 | 98.458921 |  | 1843.7117 | 99.69479 |
| 1845.576 | 99.29224 |  | 1845.576 | 98.337631 |  | 1845.576 | 99.011929 |
| 1847.4402 | 98.676428 |  | 1847.4402 | 97.991942 |  | 1847.4402 | 97.738569 |
| 1849.3044 | 98.919873 |  | 1849.3044 | 98.03994 |  | 1849.3044 | 98.235426 |
| 1851.1686 | 99.119644 |  | 1851.1686 | 98.060532 |  | 1851.1686 | 98.595822 |
| 1853.0328 | 99.321392 |  | 1853.0328 | 98.188718 |  | 1853.0328 | 98.917963 |
| 1854.8971 | 99.403094 |  | 1854.8971 | 98.335547 |  | 1854.8971 | 98.946297 |
| 1856.7613 | 99.392266 |  | 1856.7613 | 98.30657 |  | 1856.7613 | 98.980615 |
| 1858.6255 | 99.321126 |  | 1858.6255 | 98.185138 |  | 1858.6255 | 98.946482 |
| 1860.4897 | 99.258109 |  | 1860.4897 | 98.195879 |  | 1860.4897 | 98.935115 |
| 1862.3539 | 99.227711 |  | 1862.3539 | 98.237381 |  | 1862.3539 | 98.831069 |
| 1864.2181 | 99.264478 |  | 1864.2181 | 98.271625 |  | 1864.2181 | 98.959116 |
| 1866.0824 | 99.321509 |  | 1866.0824 | 98.296841 |  | 1866.0824 | 99.194398 |
| 1867.9466 | 99.453464 |  | 1867.9466 | 98.359932 |  | 1867.9466 | 99.53578 |
| 1869.8108 | 99.08003 |  | 1869.8108 | 98.230769 |  | 1869.8108 | 99.00297 |
| 1871.675 | 98.66504 |  | 1871.675 | 97.940052 |  | 1871.675 | 97.924795 |
| 1873.5392 | 98.960419 |  | 1873.5392 | 98.028578 |  | 1873.5392 | 98.155454 |
| 1875.4035 | 99.281763 |  | 1875.4035 | 98.231233 |  | 1875.4035 | 98.880292 |
| 1877.2677 | 99.35968 |  | 1877.2677 | 98.287892 |  | 1877.2677 | 98.954021 |
| 1879.1319 | 99.436323 |  | 1879.1319 | 98.306112 |  | 1879.1319 | 98.969072 |
| 1880.9961 | 99.348045 |  | 1880.9961 | 98.266262 |  | 1880.9961 | 98.928297 |
| 1882.8603 | 99.360686 |  | 1882.8603 | 98.281702 |  | 1882.8603 | 98.921067 |
| 1884.7245 | 99.417076 |  | 1884.7245 | 98.313096 |  | 1884.7245 | 98.96922 |
| 1886.5888 | 99.351444 |  | 1886.5888 | 98.282634 |  | 1886.5888 | 99.006534 |
| 1888.453 | 99.339928 |  | 1888.453 | 98.271968 |  | 1888.453 | 99.103971 |
| 1890.3172 | 99.397865 |  | 1890.3172 | 98.351828 |  | 1890.3172 | 99.272174 |
| 1892.1814 | 99.041149 |  | 1892.1814 | 98.185714 |  | 1892.1814 | 98.536878 |
| 1894.0456 | 99.142678 |  | 1894.0456 | 98.152789 |  | 1894.0456 | 98.688084 |
| 1895.9099 | 99.240986 |  | 1895.9099 | 98.137097 |  | 1895.9099 | 98.968206 |
| 1897.7741 | 99.032385 |  | 1897.7741 | 98.047249 |  | 1897.7741 | 98.755344 |
| 1899.6383 | 99.077189 |  | 1899.6383 | 98.143489 |  | 1899.6383 | 98.76563 |
| 1901.5025 | 99.270816 |  | 1901.5025 | 98.274755 |  | 1901.5025 | 98.99758 |
| 1903.3667 | 99.270862 |  | 1903.3667 | 98.243833 |  | 1903.3667 | 99.007094 |
| 1905.2309 | 99.271128 |  | 1905.2309 | 98.187677 |  | 1905.2309 | 98.960347 |
| 1907.0952 | 99.371315 |  | 1907.0952 | 98.324391 |  | 1907.0952 | 98.92789 |
| 1908.9594 | 99.441492 |  | 1908.9594 | 98.429979 |  | 1908.9594 | 99.081016 |
| 1910.8236 | 99.254373 |  | 1910.8236 | 98.323284 |  | 1910.8236 | 98.897386 |
| 1912.6878 | 99.151725 |  | 1912.6878 | 98.255831 |  | 1912.6878 | 98.676191 |
| 1914.552 | 99.318599 |  | 1914.552 | 98.31665 |  | 1914.552 | 98.682244 |
| 1916.4162 | 99.502672 |  | 1916.4162 | 98.411238 |  | 1916.4162 | 99.013826 |
| 1918.2805 | 99.544254 |  | 1918.2805 | 98.530122 |  | 1918.2805 | 99.373458 |
| 1920.1447 | 99.159835 |  | 1920.1447 | 98.459291 |  | 1920.1447 | 98.956704 |
| 1922.0089 | 99.093028 |  | 1922.0089 | 98.315979 |  | 1922.0089 | 98.689834 |
| 1923.8731 | 99.34279 |  | 1923.8731 | 98.266296 |  | 1923.8731 | 98.998073 |
| 1925.7373 | 99.158862 |  | 1925.7373 | 98.172528 |  | 1925.7373 | 98.628202 |
| 1927.6016 | 99.310517 |  | 1927.6016 | 98.337247 |  | 1927.6016 | 98.872066 |
| 1929.4658 | 99.36267 |  | 1929.4658 | 98.4588 |  | 1929.4658 | 98.965074 |
| 1931.33 | 99.411671 |  | 1931.33 | 98.458191 |  | 1931.33 | 99.019806 |
| 1933.1942 | 99.470281 |  | 1933.1942 | 98.573794 |  | 1933.1942 | 99.034312 |
| 1935.0584 | 99.539313 |  | 1935.0584 | 98.696889 |  | 1935.0584 | 99.076687 |
| 1936.9226 | 99.483118 |  | 1936.9226 | 98.549637 |  | 1936.9226 | 98.926621 |
| 1938.7869 | 99.499483 |  | 1938.7869 | 98.42331 |  | 1938.7869 | 99.058299 |
| 1940.6511 | 99.342715 |  | 1940.6511 | 98.349393 |  | 1940.6511 | 99.075684 |
| 1942.5153 | 99.469458 |  | 1942.5153 | 98.449526 |  | 1942.5153 | 99.304518 |
| 1944.3795 | 99.351449 |  | 1944.3795 | 98.37557 |  | 1944.3795 | 98.769888 |
| 1946.2437 | 99.162772 |  | 1946.2437 | 98.289798 |  | 1946.2437 | 98.534794 |
| 1948.108 | 99.27018 |  | 1948.108 | 98.561591 |  | 1948.108 | 98.93784 |
| 1949.9722 | 99.485557 |  | 1949.9722 | 98.725235 |  | 1949.9722 | 99.143291 |
| 1951.8364 | 99.582502 |  | 1951.8364 | 98.666238 |  | 1951.8364 | 98.990865 |
| 1953.7006 | 99.618909 |  | 1953.7006 | 98.73221 |  | 1953.7006 | 99.112491 |
| 1955.5648 | 99.667655 |  | 1955.5648 | 98.79645 |  | 1955.5648 | 99.199425 |
| 1957.429 | 99.687973 |  | 1957.429 | 98.867918 |  | 1957.429 | 99.230521 |
| 1959.2933 | 99.617174 |  | 1959.2933 | 98.82108 |  | 1959.2933 | 98.95838 |
| 1961.1575 | 99.599721 |  | 1961.1575 | 98.670021 |  | 1961.1575 | 99.113728 |
| 1963.0217 | 99.477088 |  | 1963.0217 | 98.619945 |  | 1963.0217 | 99.240045 |
| 1964.8859 | 99.417846 |  | 1964.8859 | 98.758327 |  | 1964.8859 | 99.238389 |
| 1966.7501 | 99.70157 |  | 1966.7501 | 99.049614 |  | 1966.7501 | 98.989293 |
| 1968.6144 | 99.99088 |  | 1968.6144 | 99.147596 |  | 1968.6144 | 98.885134 |
| 1970.4786 | 99.704852 |  | 1970.4786 | 98.833475 |  | 1970.4786 | 98.600032 |
| 1972.3428 | 99.506321 |  | 1972.3428 | 98.770638 |  | 1972.3428 | 98.917161 |
| 1974.207 | 99.354674 |  | 1974.207 | 98.94695 |  | 1974.207 | 99.073512 |
| 1976.0712 | 99.420805 |  | 1976.0712 | 98.943081 |  | 1976.0712 | 99.140031 |
| 1977.9354 | 99.549753 |  | 1977.9354 | 99.024264 |  | 1977.9354 | 98.767692 |
| 1979.7997 | 99.529097 |  | 1979.7997 | 98.963955 |  | 1979.7997 | 98.660988 |
| 1981.6639 | 99.477755 |  | 1981.6639 | 98.890703 |  | 1981.6639 | 98.463734 |
| 1983.5281 | 99.561682 |  | 1983.5281 | 98.843332 |  | 1983.5281 | 98.808289 |
| 1985.3923 | 99.498952 |  | 1985.3923 | 98.876406 |  | 1985.3923 | 98.975244 |
| 1987.2565 | 99.536747 |  | 1987.2565 | 99.063201 |  | 1987.2565 | 99.129654 |
| 1989.1208 | 99.6051 |  | 1989.1208 | 98.894805 |  | 1989.1208 | 99.020669 |
| 1990.985 | 99.469766 |  | 1990.985 | 98.514219 |  | 1990.985 | 99.080056 |
| 1992.8492 | 99.256697 |  | 1992.8492 | 98.421675 |  | 1992.8492 | 99.04393 |
| 1994.7134 | 99.146596 |  | 1994.7134 | 98.642255 |  | 1994.7134 | 98.639464 |
| 1996.5776 | 99.311246 |  | 1996.5776 | 98.961453 |  | 1996.5776 | 98.382842 |
| 1998.4418 | 99.728077 |  | 1998.4418 | 99.185974 |  | 1998.4418 | 98.767938 |
| 2000.3061 | 99.593751 |  | 2000.3061 | 98.980472 |  | 2000.3061 | 98.976357 |
| 2002.1703 | 99.63411 |  | 2002.1703 | 98.828112 |  | 2002.1703 | 99.128587 |
| 2004.0345 | 99.583448 |  | 2004.0345 | 98.943627 |  | 2004.0345 | 99.035224 |
| 2005.8987 | 99.408428 |  | 2005.8987 | 98.967841 |  | 2005.8987 | 99.100124 |
| 2007.7629 | 99.242613 |  | 2007.7629 | 98.61775 |  | 2007.7629 | 99.078173 |
| 2009.6272 | 99.377637 |  | 2009.6272 | 98.561137 |  | 2009.6272 | 98.93099 |
| 2011.4914 | 99.592845 |  | 2011.4914 | 98.682318 |  | 2011.4914 | 98.722504 |
| 2013.3556 | 99.705286 |  | 2013.3556 | 98.930378 |  | 2013.3556 | 98.984239 |
| 2015.2198 | 99.633382 |  | 2015.2198 | 99.132719 |  | 2015.2198 | 99.070602 |
| 2017.084 | 99.698446 |  | 2017.084 | 99.084372 |  | 2017.084 | 99.032654 |
| 2018.9482 | 99.460432 |  | 2018.9482 | 98.916871 |  | 2018.9482 | 98.769975 |
| 2020.8125 | 99.297888 |  | 2020.8125 | 98.774969 |  | 2020.8125 | 98.65241 |
| 2022.6767 | 99.376032 |  | 2022.6767 | 98.92868 |  | 2022.6767 | 98.373565 |
| 2024.5409 | 99.772417 |  | 2024.5409 | 99.083055 |  | 2024.5409 | 98.673224 |
| 2026.4051 | 100.02948 |  | 2026.4051 | 99.385601 |  | 2026.4051 | 98.893507 |
| 2028.2693 | 99.824836 |  | 2028.2693 | 99.440002 |  | 2028.2693 | 99.009244 |
| 2030.1336 | 99.333287 |  | 2030.1336 | 99.106124 |  | 2030.1336 | 98.944867 |
| 2031.9978 | 99.399382 |  | 2031.9978 | 99.086567 |  | 2031.9978 | 99.303431 |
| 2033.862 | 99.455236 |  | 2033.862 | 98.921038 |  | 2033.862 | 99.036667 |
| 2035.7262 | 99.487728 |  | 2035.7262 | 98.807371 |  | 2035.7262 | 98.835503 |
| 2037.5904 | 99.502969 |  | 2037.5904 | 98.929387 |  | 2037.5904 | 98.540327 |
| 2039.4546 | 99.582906 |  | 2039.4546 | 99.025723 |  | 2039.4546 | 98.554028 |
| 2041.3189 | 99.719092 |  | 2041.3189 | 99.10501 |  | 2041.3189 | 98.561455 |
| 2043.1831 | 99.655557 |  | 2043.1831 | 99.06048 |  | 2043.1831 | 98.463005 |
| 2045.0473 | 99.33798 |  | 2045.0473 | 98.853188 |  | 2045.0473 | 98.486365 |
| 2046.9115 | 99.387341 |  | 2046.9115 | 98.840872 |  | 2046.9115 | 98.871828 |
| 2048.7757 | 99.563975 |  | 2048.7757 | 98.807314 |  | 2048.7757 | 98.652286 |
| 2050.64 | 99.85758 |  | 2050.64 | 98.783053 |  | 2050.64 | 98.427194 |
| 2052.5042 | 99.646036 |  | 2052.5042 | 98.557032 |  | 2052.5042 | 98.542081 |
| 2054.3684 | 99.467401 |  | 2054.3684 | 98.663584 |  | 2054.3684 | 98.96121 |
| 2056.2326 | 99.511232 |  | 2056.2326 | 98.714358 |  | 2056.2326 | 98.786477 |
| 2058.0968 | 99.429324 |  | 2058.0968 | 98.456745 |  | 2058.0968 | 98.581365 |
| 2059.961 | 99.393661 |  | 2059.961 | 98.462556 |  | 2059.961 | 98.677443 |
| 2061.8253 | 99.367798 |  | 2061.8253 | 98.439761 |  | 2061.8253 | 98.868967 |
| 2063.6895 | 99.229757 |  | 2063.6895 | 98.322832 |  | 2063.6895 | 98.62778 |
| 2065.5537 | 99.413058 |  | 2065.5537 | 98.336836 |  | 2065.5537 | 98.655651 |
| 2067.4179 | 99.424331 |  | 2067.4179 | 98.448733 |  | 2067.4179 | 98.582382 |
| 2069.2821 | 99.38152 |  | 2069.2821 | 98.508443 |  | 2069.2821 | 98.741867 |
| 2071.1464 | 99.332155 |  | 2071.1464 | 98.32197 |  | 2071.1464 | 98.630585 |
| 2073.0106 | 99.206484 |  | 2073.0106 | 98.154348 |  | 2073.0106 | 98.552507 |
| 2074.8748 | 99.168153 |  | 2074.8748 | 98.139981 |  | 2074.8748 | 98.558179 |
| 2076.739 | 99.282523 |  | 2076.739 | 98.304874 |  | 2076.739 | 98.611852 |
| 2078.6032 | 99.360091 |  | 2078.6032 | 98.454711 |  | 2078.6032 | 98.627929 |
| 2080.4674 | 99.381701 |  | 2080.4674 | 98.438271 |  | 2080.4674 | 98.592062 |
| 2082.3317 | 99.232351 |  | 2082.3317 | 98.304465 |  | 2082.3317 | 98.528803 |
| 2084.1959 | 99.289648 |  | 2084.1959 | 98.293552 |  | 2084.1959 | 98.83852 |
| 2086.0601 | 99.323514 |  | 2086.0601 | 98.284739 |  | 2086.0601 | 98.911158 |
| 2087.9243 | 99.316868 |  | 2087.9243 | 98.237273 |  | 2087.9243 | 98.808257 |
| 2089.7885 | 99.269844 |  | 2089.7885 | 98.180852 |  | 2089.7885 | 98.693029 |
| 2091.6528 | 99.367887 |  | 2091.6528 | 98.239605 |  | 2091.6528 | 98.712159 |
| 2093.517 | 99.528966 |  | 2093.517 | 98.436038 |  | 2093.517 | 98.745219 |
| 2095.3812 | 99.538097 |  | 2095.3812 | 98.474032 |  | 2095.3812 | 98.973569 |
| 2097.2454 | 99.392843 |  | 2097.2454 | 98.389224 |  | 2097.2454 | 98.932624 |
| 2099.1096 | 99.29202 |  | 2099.1096 | 98.288754 |  | 2099.1096 | 98.722312 |
| 2100.9738 | 99.198885 |  | 2100.9738 | 98.255027 |  | 2100.9738 | 98.564584 |
| 2102.8381 | 99.275774 |  | 2102.8381 | 98.363755 |  | 2102.8381 | 98.647207 |
| 2104.7023 | 99.283882 |  | 2104.7023 | 98.31231 |  | 2104.7023 | 98.792575 |
| 2106.5665 | 99.292529 |  | 2106.5665 | 98.184331 |  | 2106.5665 | 98.867168 |
| 2108.4307 | 99.217571 |  | 2108.4307 | 98.027368 |  | 2108.4307 | 98.59931 |
| 2110.2949 | 99.273257 |  | 2110.2949 | 98.063119 |  | 2110.2949 | 98.574207 |
| 2112.1592 | 99.351309 |  | 2112.1592 | 98.308903 |  | 2112.1592 | 98.637364 |
| 2114.0234 | 99.289273 |  | 2114.0234 | 98.347399 |  | 2114.0234 | 98.769327 |
| 2115.8876 | 99.264493 |  | 2115.8876 | 98.262555 |  | 2115.8876 | 98.815034 |
| 2117.7518 | 99.422688 |  | 2117.7518 | 98.338041 |  | 2117.7518 | 98.783459 |
| 2119.616 | 99.455417 |  | 2119.616 | 98.352882 |  | 2119.616 | 98.693149 |
| 2121.4802 | 99.422355 |  | 2121.4802 | 98.258935 |  | 2121.4802 | 98.801854 |
| 2123.3445 | 99.343238 |  | 2123.3445 | 98.286996 |  | 2123.3445 | 98.856164 |
| 2125.2087 | 99.298969 |  | 2125.2087 | 98.357804 |  | 2125.2087 | 98.948429 |
| 2127.0729 | 99.310159 |  | 2127.0729 | 98.250873 |  | 2127.0729 | 99.103921 |
| 2128.9371 | 99.46054 |  | 2128.9371 | 98.256769 |  | 2128.9371 | 99.221428 |
| 2130.8013 | 99.484861 |  | 2130.8013 | 98.431099 |  | 2130.8013 | 99.083884 |
| 2132.6656 | 99.344876 |  | 2132.6656 | 98.465733 |  | 2132.6656 | 99.191541 |
| 2134.5298 | 99.330798 |  | 2134.5298 | 98.458014 |  | 2134.5298 | 99.416608 |
| 2136.394 | 99.588104 |  | 2136.394 | 98.565419 |  | 2136.394 | 99.365154 |
| 2138.2582 | 99.704146 |  | 2138.2582 | 98.719032 |  | 2138.2582 | 99.063767 |
| 2140.1224 | 99.694435 |  | 2140.1224 | 98.804109 |  | 2140.1224 | 99.120085 |
| 2141.9866 | 99.671925 |  | 2141.9866 | 98.801612 |  | 2141.9866 | 99.034604 |
| 2143.8509 | 99.558763 |  | 2143.8509 | 98.71516 |  | 2143.8509 | 99.124695 |
| 2145.7151 | 99.480375 |  | 2145.7151 | 98.893318 |  | 2145.7151 | 99.079787 |
| 2147.5793 | 99.720548 |  | 2147.5793 | 99.052513 |  | 2147.5793 | 98.940969 |
| 2149.4435 | 99.883012 |  | 2149.4435 | 99.236006 |  | 2149.4435 | 98.934319 |
| 2151.3077 | 100.07951 |  | 2151.3077 | 99.382184 |  | 2151.3077 | 99.139964 |
| 2153.172 | 99.890475 |  | 2153.172 | 99.371521 |  | 2153.172 | 99.223011 |
| 2155.0362 | 99.677031 |  | 2155.0362 | 99.294756 |  | 2155.0362 | 99.43433 |
| 2156.9004 | 99.622524 |  | 2156.9004 | 99.261175 |  | 2156.9004 | 99.460255 |
| 2158.7646 | 99.762098 |  | 2158.7646 | 99.464813 |  | 2158.7646 | 99.329126 |
| 2160.6288 | 99.655052 |  | 2160.6288 | 99.433903 |  | 2160.6288 | 98.953586 |
| 2162.493 | 99.573502 |  | 2162.493 | 99.408549 |  | 2162.493 | 98.941992 |
| 2164.3573 | 99.533099 |  | 2164.3573 | 99.260469 |  | 2164.3573 | 98.647514 |
| 2166.2215 | 99.65582 |  | 2166.2215 | 99.399453 |  | 2166.2215 | 98.688543 |
| 2168.0857 | 99.543378 |  | 2168.0857 | 99.198335 |  | 2168.0857 | 98.789872 |
| 2169.9499 | 99.370039 |  | 2169.9499 | 98.806707 |  | 2169.9499 | 98.673811 |
| 2171.8141 | 99.433879 |  | 2171.8141 | 98.774864 |  | 2171.8141 | 98.387758 |
| 2173.6784 | 99.589353 |  | 2173.6784 | 98.712617 |  | 2173.6784 | 98.530577 |
| 2175.5426 | 99.836181 |  | 2175.5426 | 98.895176 |  | 2175.5426 | 98.556642 |
| 2177.4068 | 99.888555 |  | 2177.4068 | 99.095358 |  | 2177.4068 | 98.658414 |
| 2179.271 | 99.634569 |  | 2179.271 | 98.877115 |  | 2179.271 | 98.763319 |
| 2181.1352 | 99.507487 |  | 2181.1352 | 98.860465 |  | 2181.1352 | 99.036261 |
| 2182.9994 | 99.565025 |  | 2182.9994 | 98.936381 |  | 2182.9994 | 99.034166 |
| 2184.8637 | 99.5171 |  | 2184.8637 | 98.696696 |  | 2184.8637 | 98.890075 |
| 2186.7279 | 99.275817 |  | 2186.7279 | 98.565821 |  | 2186.7279 | 98.615227 |
| 2188.5921 | 98.919044 |  | 2188.5921 | 98.478945 |  | 2188.5921 | 98.530519 |
| 2190.4563 | 99.07262 |  | 2190.4563 | 98.438396 |  | 2190.4563 | 98.651811 |
| 2192.3205 | 99.603177 |  | 2192.3205 | 98.668194 |  | 2192.3205 | 98.852386 |
| 2194.1848 | 99.838334 |  | 2194.1848 | 98.942951 |  | 2194.1848 | 98.761117 |
| 2196.049 | 99.837033 |  | 2196.049 | 99.090188 |  | 2196.049 | 98.769554 |
| 2197.9132 | 99.545828 |  | 2197.9132 | 99.004453 |  | 2197.9132 | 98.867209 |
| 2199.7774 | 99.542861 |  | 2199.7774 | 98.936224 |  | 2199.7774 | 99.003103 |
| 2201.6416 | 99.428721 |  | 2201.6416 | 98.743339 |  | 2201.6416 | 98.868935 |
| 2203.5058 | 99.147085 |  | 2203.5058 | 98.492506 |  | 2203.5058 | 98.667831 |
| 2205.3701 | 99.253438 |  | 2205.3701 | 98.393135 |  | 2205.3701 | 98.618102 |
| 2207.2343 | 99.454641 |  | 2207.2343 | 98.43796 |  | 2207.2343 | 98.716222 |
| 2209.0985 | 99.281175 |  | 2209.0985 | 98.457188 |  | 2209.0985 | 98.816447 |
| 2210.9627 | 99.002939 |  | 2210.9627 | 98.286921 |  | 2210.9627 | 98.967459 |
| 2212.8269 | 99.019199 |  | 2212.8269 | 98.177653 |  | 2212.8269 | 98.733529 |
| 2214.6912 | 99.14407 |  | 2214.6912 | 98.0359 |  | 2214.6912 | 98.674254 |
| 2216.5554 | 99.214986 |  | 2216.5554 | 98.218928 |  | 2216.5554 | 98.676853 |
| 2218.4196 | 99.379099 |  | 2218.4196 | 98.51686 |  | 2218.4196 | 98.64478 |
| 2220.2838 | 99.372916 |  | 2220.2838 | 98.433666 |  | 2220.2838 | 98.616693 |
| 2222.148 | 99.43747 |  | 2222.148 | 98.394476 |  | 2222.148 | 98.714833 |
| 2224.0122 | 99.47319 |  | 2224.0122 | 98.540249 |  | 2224.0122 | 98.650886 |
| 2225.8765 | 99.257513 |  | 2225.8765 | 98.436966 |  | 2225.8765 | 98.696096 |
| 2227.7407 | 99.321962 |  | 2227.7407 | 98.363825 |  | 2227.7407 | 98.665622 |
| 2229.6049 | 99.625458 |  | 2229.6049 | 98.434435 |  | 2229.6049 | 98.812892 |
| 2231.4691 | 99.509828 |  | 2231.4691 | 98.449525 |  | 2231.4691 | 98.77177 |
| 2233.3333 | 99.335511 |  | 2233.3333 | 98.326652 |  | 2233.3333 | 98.62445 |
| 2235.1976 | 99.293248 |  | 2235.1976 | 98.333104 |  | 2235.1976 | 98.629799 |
| 2237.0618 | 99.268321 |  | 2237.0618 | 98.307962 |  | 2237.0618 | 98.63464 |
| 2238.926 | 99.229404 |  | 2238.926 | 98.191368 |  | 2238.926 | 98.65409 |
| 2240.7902 | 99.292187 |  | 2240.7902 | 98.257921 |  | 2240.7902 | 98.842994 |
| 2242.6544 | 99.220761 |  | 2242.6544 | 98.279445 |  | 2242.6544 | 98.825208 |
| 2244.5186 | 99.175184 |  | 2244.5186 | 98.168006 |  | 2244.5186 | 98.616396 |
| 2246.3829 | 99.171631 |  | 2246.3829 | 98.070474 |  | 2246.3829 | 98.533689 |
| 2248.2471 | 99.410358 |  | 2248.2471 | 98.231963 |  | 2248.2471 | 98.670452 |
| 2250.1113 | 99.506003 |  | 2250.1113 | 98.264932 |  | 2250.1113 | 98.683441 |
| 2251.9755 | 99.115866 |  | 2251.9755 | 98.06519 |  | 2251.9755 | 98.657731 |
| 2253.8397 | 99.121073 |  | 2253.8397 | 98.00424 |  | 2253.8397 | 98.679119 |
| 2255.704 | 99.375326 |  | 2255.704 | 98.051696 |  | 2255.704 | 98.710835 |
| 2257.5682 | 99.139861 |  | 2257.5682 | 97.999784 |  | 2257.5682 | 98.53685 |
| 2259.4324 | 98.944972 |  | 2259.4324 | 97.906875 |  | 2259.4324 | 98.454288 |
| 2261.2966 | 99.057493 |  | 2261.2966 | 97.96495 |  | 2261.2966 | 98.557888 |
| 2263.1608 | 99.070298 |  | 2263.1608 | 97.96449 |  | 2263.1608 | 98.730306 |
| 2265.025 | 99.050096 |  | 2265.025 | 97.947836 |  | 2265.025 | 98.487652 |
| 2266.8893 | 99.229085 |  | 2266.8893 | 98.103808 |  | 2266.8893 | 98.385537 |
| 2268.7535 | 99.213497 |  | 2268.7535 | 98.144114 |  | 2268.7535 | 98.581779 |
| 2270.6177 | 99.01219 |  | 2270.6177 | 98.013908 |  | 2270.6177 | 98.669062 |
| 2272.4819 | 99.040119 |  | 2272.4819 | 97.889919 |  | 2272.4819 | 98.555012 |
| 2274.3461 | 99.066555 |  | 2274.3461 | 97.880211 |  | 2274.3461 | 98.5092 |
| 2276.2104 | 98.994936 |  | 2276.2104 | 97.852571 |  | 2276.2104 | 98.446943 |
| 2278.0746 | 99.004343 |  | 2278.0746 | 97.760514 |  | 2278.0746 | 98.43865 |
| 2279.9388 | 99.037808 |  | 2279.9388 | 97.708692 |  | 2279.9388 | 98.399618 |
| 2281.803 | 99.088341 |  | 2281.803 | 97.773859 |  | 2281.803 | 98.358915 |
| 2283.6672 | 98.999777 |  | 2283.6672 | 97.728677 |  | 2283.6672 | 98.342304 |
| 2285.5314 | 98.913934 |  | 2285.5314 | 97.722718 |  | 2285.5314 | 98.349654 |
| 2287.3957 | 98.936429 |  | 2287.3957 | 97.726838 |  | 2287.3957 | 98.317219 |
| 2289.2599 | 98.985647 |  | 2289.2599 | 97.727403 |  | 2289.2599 | 98.323089 |
| 2291.1241 | 98.990889 |  | 2291.1241 | 97.689579 |  | 2291.1241 | 98.313483 |
| 2292.9883 | 98.934306 |  | 2292.9883 | 97.650603 |  | 2292.9883 | 98.362031 |
| 2294.8525 | 98.907407 |  | 2294.8525 | 97.698741 |  | 2294.8525 | 98.339505 |
| 2296.7168 | 98.919113 |  | 2296.7168 | 97.741588 |  | 2296.7168 | 98.381096 |
| 2298.581 | 98.864456 |  | 2298.581 | 97.746769 |  | 2298.581 | 98.497418 |
| 2300.4452 | 98.805314 |  | 2300.4452 | 97.76151 |  | 2300.4452 | 98.55892 |
| 2302.3094 | 98.789951 |  | 2302.3094 | 97.784315 |  | 2302.3094 | 98.530398 |
| 2304.1736 | 98.744728 |  | 2304.1736 | 97.770276 |  | 2304.1736 | 98.547068 |
| 2306.0378 | 98.638548 |  | 2306.0378 | 97.688401 |  | 2306.0378 | 98.545684 |
| 2307.9021 | 98.628686 |  | 2307.9021 | 97.700883 |  | 2307.9021 | 98.554088 |
| 2309.7663 | 98.596148 |  | 2309.7663 | 97.743818 |  | 2309.7663 | 98.476466 |
| 2311.6305 | 98.459625 |  | 2311.6305 | 97.726531 |  | 2311.6305 | 98.429095 |
| 2313.4947 | 98.288725 |  | 2313.4947 | 97.69493 |  | 2313.4947 | 98.487912 |
| 2315.3589 | 98.226638 |  | 2315.3589 | 97.722937 |  | 2315.3589 | 98.589523 |
| 2317.2232 | 98.174304 |  | 2317.2232 | 97.702368 |  | 2317.2232 | 98.581474 |
| 2319.0874 | 98.043011 |  | 2319.0874 | 97.615618 |  | 2319.0874 | 98.529187 |
| 2320.9516 | 97.890909 |  | 2320.9516 | 97.619154 |  | 2320.9516 | 98.506855 |
| 2322.8158 | 97.73635 |  | 2322.8158 | 97.601184 |  | 2322.8158 | 98.484105 |
| 2324.68 | 97.605471 |  | 2324.68 | 97.570948 |  | 2324.68 | 98.371906 |
| 2326.5442 | 97.525546 |  | 2326.5442 | 97.608285 |  | 2326.5442 | 98.37385 |
| 2328.4085 | 97.549495 |  | 2328.4085 | 97.68895 |  | 2328.4085 | 98.618852 |
| 2330.2727 | 97.575949 |  | 2330.2727 | 97.74284 |  | 2330.2727 | 98.860357 |
| 2332.1369 | 97.543651 |  | 2332.1369 | 97.787726 |  | 2332.1369 | 98.868969 |
| 2334.0011 | 97.51849 |  | 2334.0011 | 97.832802 |  | 2334.0011 | 98.797604 |
| 2335.8653 | 97.424626 |  | 2335.8653 | 97.797173 |  | 2335.8653 | 98.720536 |
| 2337.7295 | 97.453415 |  | 2337.7295 | 97.800249 |  | 2337.7295 | 98.776976 |
| 2339.5938 | 97.490215 |  | 2339.5938 | 97.787972 |  | 2339.5938 | 98.845737 |
| 2341.458 | 97.375775 |  | 2341.458 | 97.685384 |  | 2341.458 | 98.882079 |
| 2343.3222 | 97.214131 |  | 2343.3222 | 97.606301 |  | 2343.3222 | 98.741251 |
| 2345.1864 | 97.189278 |  | 2345.1864 | 97.634368 |  | 2345.1864 | 98.538397 |
| 2347.0506 | 97.236621 |  | 2347.0506 | 97.68874 |  | 2347.0506 | 98.404003 |
| 2348.9149 | 97.483599 |  | 2348.9149 | 97.706695 |  | 2348.9149 | 98.347818 |
| 2350.7791 | 97.891623 |  | 2350.7791 | 97.72684 |  | 2350.7791 | 98.631487 |
| 2352.6433 | 98.076427 |  | 2352.6433 | 97.866277 |  | 2352.6433 | 99.222001 |
| 2354.5075 | 97.793743 |  | 2354.5075 | 97.976117 |  | 2354.5075 | 99.529565 |
| 2356.3717 | 97.389647 |  | 2356.3717 | 97.985953 |  | 2356.3717 | 99.402949 |
| 2358.2359 | 97.05147 |  | 2358.2359 | 97.966033 |  | 2358.2359 | 99.132619 |
| 2360.1002 | 96.888477 |  | 2360.1002 | 97.912252 |  | 2360.1002 | 98.983069 |
| 2361.9644 | 96.87564 |  | 2361.9644 | 97.856303 |  | 2361.9644 | 98.979309 |
| 2363.8286 | 96.842263 |  | 2363.8286 | 97.871768 |  | 2363.8286 | 98.961423 |
| 2365.6928 | 96.909203 |  | 2365.6928 | 97.939076 |  | 2365.6928 | 98.879475 |
| 2367.557 | 97.003728 |  | 2367.557 | 97.8965 |  | 2367.557 | 98.694834 |
| 2369.4213 | 97.06504 |  | 2369.4213 | 97.848137 |  | 2369.4213 | 98.532262 |
| 2371.2855 | 97.060738 |  | 2371.2855 | 97.777745 |  | 2371.2855 | 98.432235 |
| 2373.1497 | 97.23255 |  | 2373.1497 | 97.720245 |  | 2373.1497 | 98.409503 |
| 2375.0139 | 97.569135 |  | 2375.0139 | 97.730139 |  | 2375.0139 | 98.429568 |
| 2376.8781 | 97.773272 |  | 2376.8781 | 97.720195 |  | 2376.8781 | 98.339633 |
| 2378.7423 | 97.984134 |  | 2378.7423 | 97.698962 |  | 2378.7423 | 98.250693 |
| 2380.6066 | 98.252049 |  | 2380.6066 | 97.665985 |  | 2380.6066 | 98.222612 |
| 2382.4708 | 98.473696 |  | 2382.4708 | 97.629364 |  | 2382.4708 | 98.319897 |
| 2384.335 | 98.713942 |  | 2384.335 | 97.65189 |  | 2384.335 | 98.454732 |
| 2386.1992 | 98.964707 |  | 2386.1992 | 97.704005 |  | 2386.1992 | 98.520023 |
| 2388.0634 | 99.085661 |  | 2388.0634 | 97.696486 |  | 2388.0634 | 98.523262 |
| 2389.9277 | 99.09838 |  | 2389.9277 | 97.69488 |  | 2389.9277 | 98.531828 |
| 2391.7919 | 99.152358 |  | 2391.7919 | 97.755194 |  | 2391.7919 | 98.558078 |
| 2393.6561 | 99.209764 |  | 2393.6561 | 97.799913 |  | 2393.6561 | 98.602761 |
| 2395.5203 | 99.164257 |  | 2395.5203 | 97.783392 |  | 2395.5203 | 98.627619 |
| 2397.3845 | 99.131736 |  | 2397.3845 | 97.806736 |  | 2397.3845 | 98.625577 |
| 2399.2487 | 99.149782 |  | 2399.2487 | 97.804637 |  | 2399.2487 | 98.611914 |
| 2401.113 | 99.139066 |  | 2401.113 | 97.709976 |  | 2401.113 | 98.644723 |
| 2402.9772 | 99.081071 |  | 2402.9772 | 97.669502 |  | 2402.9772 | 98.63281 |
| 2404.8414 | 99.081926 |  | 2404.8414 | 97.775658 |  | 2404.8414 | 98.56679 |
| 2406.7056 | 99.136928 |  | 2406.7056 | 97.840365 |  | 2406.7056 | 98.579552 |
| 2408.5698 | 99.141659 |  | 2408.5698 | 97.756982 |  | 2408.5698 | 98.62458 |
| 2410.4341 | 99.101086 |  | 2410.4341 | 97.688077 |  | 2410.4341 | 98.584032 |
| 2412.2983 | 99.124685 |  | 2412.2983 | 97.714895 |  | 2412.2983 | 98.550343 |
| 2414.1625 | 99.12332 |  | 2414.1625 | 97.717357 |  | 2414.1625 | 98.543098 |
| 2416.0267 | 99.116466 |  | 2416.0267 | 97.668955 |  | 2416.0267 | 98.589602 |
| 2417.8909 | 99.171968 |  | 2417.8909 | 97.700156 |  | 2417.8909 | 98.621765 |
| 2419.7551 | 99.15652 |  | 2419.7551 | 97.688375 |  | 2419.7551 | 98.600451 |
| 2421.6194 | 99.120992 |  | 2421.6194 | 97.629395 |  | 2421.6194 | 98.518379 |
| 2423.4836 | 99.118938 |  | 2423.4836 | 97.63131 |  | 2423.4836 | 98.505998 |
| 2425.3478 | 99.070363 |  | 2425.3478 | 97.608987 |  | 2425.3478 | 98.537676 |
| 2427.212 | 99.069995 |  | 2427.212 | 97.653168 |  | 2427.212 | 98.578027 |
| 2429.0762 | 99.130799 |  | 2429.0762 | 97.746098 |  | 2429.0762 | 98.566174 |
| 2430.9405 | 99.165105 |  | 2430.9405 | 97.730747 |  | 2430.9405 | 98.550145 |
| 2432.8047 | 99.145783 |  | 2432.8047 | 97.662422 |  | 2432.8047 | 98.493149 |
| 2434.6689 | 99.089466 |  | 2434.6689 | 97.646259 |  | 2434.6689 | 98.516731 |
| 2436.5331 | 99.050994 |  | 2436.5331 | 97.62474 |  | 2436.5331 | 98.578962 |
| 2438.3973 | 99.019971 |  | 2438.3973 | 97.627293 |  | 2438.3973 | 98.585413 |
| 2440.2615 | 99.016933 |  | 2440.2615 | 97.668727 |  | 2440.2615 | 98.48542 |
| 2442.1258 | 99.05977 |  | 2442.1258 | 97.676302 |  | 2442.1258 | 98.428354 |
| 2443.99 | 99.09719 |  | 2443.99 | 97.61824 |  | 2443.99 | 98.459228 |
| 2445.8542 | 99.114766 |  | 2445.8542 | 97.573556 |  | 2445.8542 | 98.441297 |
| 2447.7184 | 99.126213 |  | 2447.7184 | 97.620708 |  | 2447.7184 | 98.440096 |
| 2449.5826 | 99.081677 |  | 2449.5826 | 97.637576 |  | 2449.5826 | 98.466118 |
| 2451.4469 | 99.068662 |  | 2451.4469 | 97.616701 |  | 2451.4469 | 98.434272 |
| 2453.3111 | 99.1083 |  | 2453.3111 | 97.644441 |  | 2453.3111 | 98.477565 |
| 2455.1753 | 99.12973 |  | 2455.1753 | 97.667441 |  | 2455.1753 | 98.478558 |
| 2457.0395 | 99.144929 |  | 2457.0395 | 97.680861 |  | 2457.0395 | 98.431918 |
| 2458.9037 | 99.09982 |  | 2458.9037 | 97.663353 |  | 2458.9037 | 98.460634 |
| 2460.7679 | 99.064391 |  | 2460.7679 | 97.63811 |  | 2460.7679 | 98.487821 |
| 2462.6322 | 99.055821 |  | 2462.6322 | 97.57214 |  | 2462.6322 | 98.444257 |
| 2464.4964 | 99.050604 |  | 2464.4964 | 97.538174 |  | 2464.4964 | 98.418401 |
| 2466.3606 | 99.053132 |  | 2466.3606 | 97.547821 |  | 2466.3606 | 98.353192 |
| 2468.2248 | 99.117446 |  | 2468.2248 | 97.578538 |  | 2468.2248 | 98.345278 |
| 2470.089 | 99.173496 |  | 2470.089 | 97.639347 |  | 2470.089 | 98.413047 |
| 2471.9533 | 99.163885 |  | 2471.9533 | 97.693527 |  | 2471.9533 | 98.404572 |
| 2473.8175 | 99.116363 |  | 2473.8175 | 97.647892 |  | 2473.8175 | 98.334322 |
| 2475.6817 | 99.100289 |  | 2475.6817 | 97.558535 |  | 2475.6817 | 98.317919 |
| 2477.5459 | 99.093034 |  | 2477.5459 | 97.561558 |  | 2477.5459 | 98.307553 |
| 2479.4101 | 99.028282 |  | 2479.4101 | 97.571638 |  | 2479.4101 | 98.370358 |
| 2481.2743 | 99.027174 |  | 2481.2743 | 97.537412 |  | 2481.2743 | 98.482501 |
| 2483.1386 | 99.09771 |  | 2483.1386 | 97.561259 |  | 2483.1386 | 98.516788 |
| 2485.0028 | 99.155536 |  | 2485.0028 | 97.606355 |  | 2485.0028 | 98.435831 |
| 2486.867 | 99.171754 |  | 2486.867 | 97.632482 |  | 2486.867 | 98.385584 |
| 2488.7312 | 99.145486 |  | 2488.7312 | 97.626647 |  | 2488.7312 | 98.342093 |
| 2490.5954 | 99.111977 |  | 2490.5954 | 97.588345 |  | 2490.5954 | 98.2955 |
| 2492.4597 | 99.095345 |  | 2492.4597 | 97.57081 |  | 2492.4597 | 98.313006 |
| 2494.3239 | 99.025927 |  | 2494.3239 | 97.520075 |  | 2494.3239 | 98.346063 |
| 2496.1881 | 98.96869 |  | 2496.1881 | 97.434694 |  | 2496.1881 | 98.313533 |
| 2498.0523 | 99.068302 |  | 2498.0523 | 97.474209 |  | 2498.0523 | 98.31329 |
| 2499.9165 | 99.11612 |  | 2499.9165 | 97.530869 |  | 2499.9165 | 98.3442 |
| 2501.7807 | 99.022341 |  | 2501.7807 | 97.522912 |  | 2501.7807 | 98.382424 |
| 2503.645 | 99.042691 |  | 2503.645 | 97.515047 |  | 2503.645 | 98.354166 |
| 2505.5092 | 99.078583 |  | 2505.5092 | 97.528986 |  | 2505.5092 | 98.350838 |
| 2507.3734 | 99.031722 |  | 2507.3734 | 97.542122 |  | 2507.3734 | 98.372492 |
| 2509.2376 | 99.10104 |  | 2509.2376 | 97.58295 |  | 2509.2376 | 98.326184 |
| 2511.1018 | 99.121122 |  | 2511.1018 | 97.593978 |  | 2511.1018 | 98.29019 |
| 2512.9661 | 99.028463 |  | 2512.9661 | 97.531833 |  | 2512.9661 | 98.315905 |
| 2514.8303 | 99.03256 |  | 2514.8303 | 97.514949 |  | 2514.8303 | 98.332398 |
| 2516.6945 | 99.073022 |  | 2516.6945 | 97.566708 |  | 2516.6945 | 98.409061 |
| 2518.5587 | 99.082065 |  | 2518.5587 | 97.584558 |  | 2518.5587 | 98.452785 |
| 2520.4229 | 99.04211 |  | 2520.4229 | 97.515708 |  | 2520.4229 | 98.400823 |
| 2522.2871 | 99.008163 |  | 2522.2871 | 97.442799 |  | 2522.2871 | 98.324388 |
| 2524.1514 | 99.065289 |  | 2524.1514 | 97.46326 |  | 2524.1514 | 98.337301 |
| 2526.0156 | 99.102232 |  | 2526.0156 | 97.508931 |  | 2526.0156 | 98.391964 |
| 2527.8798 | 99.091475 |  | 2527.8798 | 97.522959 |  | 2527.8798 | 98.316228 |
| 2529.744 | 99.048184 |  | 2529.744 | 97.485388 |  | 2529.744 | 98.237085 |
| 2531.6082 | 99.016006 |  | 2531.6082 | 97.4229 |  | 2531.6082 | 98.277858 |
| 2533.4725 | 99.051964 |  | 2533.4725 | 97.455312 |  | 2533.4725 | 98.292548 |
| 2535.3367 | 99.083729 |  | 2535.3367 | 97.522135 |  | 2535.3367 | 98.309843 |
| 2537.2009 | 99.081256 |  | 2537.2009 | 97.495289 |  | 2537.2009 | 98.315269 |
| 2539.0651 | 99.094734 |  | 2539.0651 | 97.486961 |  | 2539.0651 | 98.21982 |
| 2540.9293 | 99.10477 |  | 2540.9293 | 97.534749 |  | 2540.9293 | 98.19171 |
| 2542.7935 | 99.090646 |  | 2542.7935 | 97.517599 |  | 2542.7935 | 98.236345 |
| 2544.6578 | 99.081339 |  | 2544.6578 | 97.482157 |  | 2544.6578 | 98.246951 |
| 2546.522 | 99.076091 |  | 2546.522 | 97.487092 |  | 2546.522 | 98.289211 |
| 2548.3862 | 99.082792 |  | 2548.3862 | 97.518843 |  | 2548.3862 | 98.312592 |
| 2550.2504 | 99.041192 |  | 2550.2504 | 97.468027 |  | 2550.2504 | 98.25116 |
| 2552.1146 | 98.941365 |  | 2552.1146 | 97.360646 |  | 2552.1146 | 98.235244 |
| 2553.9789 | 98.932089 |  | 2553.9789 | 97.324969 |  | 2553.9789 | 98.271056 |
| 2555.8431 | 99.025896 |  | 2555.8431 | 97.353799 |  | 2555.8431 | 98.316443 |
| 2557.7073 | 99.028118 |  | 2557.7073 | 97.367508 |  | 2557.7073 | 98.333539 |
| 2559.5715 | 98.981261 |  | 2559.5715 | 97.362482 |  | 2559.5715 | 98.288986 |
| 2561.4357 | 99.033913 |  | 2561.4357 | 97.45618 |  | 2561.4357 | 98.218362 |
| 2563.2999 | 99.073934 |  | 2563.2999 | 97.547166 |  | 2563.2999 | 98.160226 |
| 2565.1642 | 99.062382 |  | 2565.1642 | 97.480083 |  | 2565.1642 | 98.183183 |
| 2567.0284 | 99.100135 |  | 2567.0284 | 97.40245 |  | 2567.0284 | 98.252149 |
| 2568.8926 | 99.06783 |  | 2568.8926 | 97.389127 |  | 2568.8926 | 98.300389 |
| 2570.7568 | 99.018299 |  | 2570.7568 | 97.347936 |  | 2570.7568 | 98.288379 |
| 2572.621 | 99.020504 |  | 2572.621 | 97.308979 |  | 2572.621 | 98.239219 |
| 2574.4853 | 98.995933 |  | 2574.4853 | 97.315962 |  | 2574.4853 | 98.222315 |
| 2576.3495 | 98.977057 |  | 2576.3495 | 97.336342 |  | 2576.3495 | 98.2158 |
| 2578.2137 | 98.954416 |  | 2578.2137 | 97.347118 |  | 2578.2137 | 98.217981 |
| 2580.0779 | 98.96727 |  | 2580.0779 | 97.347151 |  | 2580.0779 | 98.196196 |
| 2581.9421 | 99.026877 |  | 2581.9421 | 97.389854 |  | 2581.9421 | 98.165185 |
| 2583.8063 | 99.033304 |  | 2583.8063 | 97.409814 |  | 2583.8063 | 98.168184 |
| 2585.6706 | 98.98395 |  | 2585.6706 | 97.32387 |  | 2585.6706 | 98.140504 |
| 2587.5348 | 98.966985 |  | 2587.5348 | 97.263766 |  | 2587.5348 | 98.134088 |
| 2589.399 | 99.010614 |  | 2589.399 | 97.318311 |  | 2589.399 | 98.155171 |
| 2591.2632 | 99.006892 |  | 2591.2632 | 97.30244 |  | 2591.2632 | 98.104984 |
| 2593.1274 | 98.970683 |  | 2593.1274 | 97.281656 |  | 2593.1274 | 98.101261 |
| 2594.9917 | 98.965005 |  | 2594.9917 | 97.317166 |  | 2594.9917 | 98.168236 |
| 2596.8559 | 98.96491 |  | 2596.8559 | 97.258217 |  | 2596.8559 | 98.161877 |
| 2598.7201 | 98.948611 |  | 2598.7201 | 97.217289 |  | 2598.7201 | 98.116191 |
| 2600.5843 | 98.949808 |  | 2600.5843 | 97.24513 |  | 2600.5843 | 98.127513 |
| 2602.4485 | 98.961497 |  | 2602.4485 | 97.252237 |  | 2602.4485 | 98.125546 |
| 2604.3127 | 98.943079 |  | 2604.3127 | 97.239008 |  | 2604.3127 | 98.073765 |
| 2606.177 | 98.940534 |  | 2606.177 | 97.217752 |  | 2606.177 | 98.045319 |
| 2608.0412 | 98.975459 |  | 2608.0412 | 97.212526 |  | 2608.0412 | 98.066853 |
| 2609.9054 | 98.954322 |  | 2609.9054 | 97.176228 |  | 2609.9054 | 98.051664 |
| 2611.7696 | 98.919093 |  | 2611.7696 | 97.146044 |  | 2611.7696 | 98.014926 |
| 2613.6338 | 98.917091 |  | 2613.6338 | 97.168296 |  | 2613.6338 | 98.046418 |
| 2615.4981 | 98.914673 |  | 2615.4981 | 97.166862 |  | 2615.4981 | 98.082646 |
| 2617.3623 | 98.919269 |  | 2617.3623 | 97.153676 |  | 2617.3623 | 98.05351 |
| 2619.2265 | 98.936973 |  | 2619.2265 | 97.174848 |  | 2619.2265 | 98.037308 |
| 2621.0907 | 98.942451 |  | 2621.0907 | 97.1777 |  | 2621.0907 | 98.047453 |
| 2622.9549 | 98.903331 |  | 2622.9549 | 97.133279 |  | 2622.9549 | 98.032826 |
| 2624.8191 | 98.861226 |  | 2624.8191 | 97.101935 |  | 2624.8191 | 98.001993 |
| 2626.6834 | 98.866949 |  | 2626.6834 | 97.08266 |  | 2626.6834 | 97.970257 |
| 2628.5476 | 98.883003 |  | 2628.5476 | 97.08088 |  | 2628.5476 | 97.939888 |
| 2630.4118 | 98.928544 |  | 2630.4118 | 97.127327 |  | 2630.4118 | 97.964332 |
| 2632.276 | 98.945681 |  | 2632.276 | 97.122936 |  | 2632.276 | 97.962582 |
| 2634.1402 | 98.893636 |  | 2634.1402 | 97.075447 |  | 2634.1402 | 97.919126 |
| 2636.0045 | 98.891307 |  | 2636.0045 | 97.089205 |  | 2636.0045 | 97.905025 |
| 2637.8687 | 98.919582 |  | 2637.8687 | 97.129937 |  | 2637.8687 | 97.916415 |
| 2639.7329 | 98.883908 |  | 2639.7329 | 97.094021 |  | 2639.7329 | 97.930847 |
| 2641.5971 | 98.850727 |  | 2641.5971 | 97.05174 |  | 2641.5971 | 97.951903 |
| 2643.4613 | 98.848206 |  | 2643.4613 | 97.048803 |  | 2643.4613 | 97.938979 |
| 2645.3255 | 98.859585 |  | 2645.3255 | 97.026822 |  | 2645.3255 | 97.895775 |
| 2647.1898 | 98.851767 |  | 2647.1898 | 97.0055 |  | 2647.1898 | 97.846637 |
| 2649.054 | 98.859795 |  | 2649.054 | 96.988815 |  | 2649.054 | 97.864695 |
| 2650.9182 | 98.915429 |  | 2650.9182 | 96.994884 |  | 2650.9182 | 97.910037 |
| 2652.7824 | 98.896726 |  | 2652.7824 | 96.991601 |  | 2652.7824 | 97.884115 |
| 2654.6466 | 98.838092 |  | 2654.6466 | 96.944407 |  | 2654.6466 | 97.851875 |
| 2656.5109 | 98.843182 |  | 2656.5109 | 96.948173 |  | 2656.5109 | 97.864231 |
| 2658.3751 | 98.845937 |  | 2658.3751 | 96.97099 |  | 2658.3751 | 97.893346 |
| 2660.2393 | 98.837932 |  | 2660.2393 | 96.969132 |  | 2660.2393 | 97.924666 |
| 2662.1035 | 98.868396 |  | 2662.1035 | 96.982452 |  | 2662.1035 | 97.915915 |
| 2663.9677 | 98.892332 |  | 2663.9677 | 97.020228 |  | 2663.9677 | 97.897944 |
| 2665.8319 | 98.888458 |  | 2665.8319 | 97.043667 |  | 2665.8319 | 97.897348 |
| 2667.6962 | 98.920009 |  | 2667.6962 | 97.03473 |  | 2667.6962 | 97.919004 |
| 2669.5604 | 98.936116 |  | 2669.5604 | 97.03164 |  | 2669.5604 | 97.94411 |
| 2671.4246 | 98.90702 |  | 2671.4246 | 97.026662 |  | 2671.4246 | 97.97731 |
| 2673.2888 | 98.889963 |  | 2673.2888 | 96.991639 |  | 2673.2888 | 97.97984 |
| 2675.153 | 98.904714 |  | 2675.153 | 96.986619 |  | 2675.153 | 97.954154 |
| 2677.0173 | 98.905907 |  | 2677.0173 | 96.980658 |  | 2677.0173 | 97.941826 |
| 2678.8815 | 98.913633 |  | 2678.8815 | 96.947186 |  | 2678.8815 | 97.944823 |
| 2680.7457 | 98.921929 |  | 2680.7457 | 96.931453 |  | 2680.7457 | 97.945786 |
| 2682.6099 | 98.896864 |  | 2682.6099 | 96.937893 |  | 2682.6099 | 97.96504 |
| 2684.4741 | 98.889633 |  | 2684.4741 | 96.948965 |  | 2684.4741 | 97.980841 |
| 2686.3383 | 98.899062 |  | 2686.3383 | 96.950355 |  | 2686.3383 | 97.984095 |
| 2688.2026 | 98.881694 |  | 2688.2026 | 96.956638 |  | 2688.2026 | 97.975913 |
| 2690.0668 | 98.870154 |  | 2690.0668 | 96.971863 |  | 2690.0668 | 97.962524 |
| 2691.931 | 98.879714 |  | 2691.931 | 96.962012 |  | 2691.931 | 97.911729 |
| 2693.7952 | 98.876761 |  | 2693.7952 | 96.948338 |  | 2693.7952 | 97.875878 |
| 2695.6594 | 98.863953 |  | 2695.6594 | 96.967584 |  | 2695.6594 | 97.891598 |
| 2697.5237 | 98.867252 |  | 2697.5237 | 96.971628 |  | 2697.5237 | 97.934622 |
| 2699.3879 | 98.891847 |  | 2699.3879 | 96.951195 |  | 2699.3879 | 97.956453 |
| 2701.2521 | 98.916573 |  | 2701.2521 | 96.951634 |  | 2701.2521 | 97.965221 |
| 2703.1163 | 98.928243 |  | 2703.1163 | 96.950059 |  | 2703.1163 | 97.964997 |
| 2704.9805 | 98.922376 |  | 2704.9805 | 96.935508 |  | 2704.9805 | 97.96226 |
| 2706.8447 | 98.880192 |  | 2706.8447 | 96.88134 |  | 2706.8447 | 97.963961 |
| 2708.709 | 98.861382 |  | 2708.709 | 96.853791 |  | 2708.709 | 97.966572 |
| 2710.5732 | 98.888527 |  | 2710.5732 | 96.90688 |  | 2710.5732 | 97.943627 |
| 2712.4374 | 98.899854 |  | 2712.4374 | 96.931135 |  | 2712.4374 | 97.96166 |
| 2714.3016 | 98.875141 |  | 2714.3016 | 96.880248 |  | 2714.3016 | 97.998658 |
| 2716.1658 | 98.849591 |  | 2716.1658 | 96.844973 |  | 2716.1658 | 97.981357 |
| 2718.0301 | 98.864202 |  | 2718.0301 | 96.86561 |  | 2718.0301 | 97.965003 |
| 2719.8943 | 98.913573 |  | 2719.8943 | 96.889276 |  | 2719.8943 | 97.982634 |
| 2721.7585 | 98.916529 |  | 2721.7585 | 96.872658 |  | 2721.7585 | 97.957027 |
| 2723.6227 | 98.866108 |  | 2723.6227 | 96.842488 |  | 2723.6227 | 97.913613 |
| 2725.4869 | 98.854167 |  | 2725.4869 | 96.850904 |  | 2725.4869 | 97.91837 |
| 2727.3511 | 98.876712 |  | 2727.3511 | 96.86862 |  | 2727.3511 | 97.938445 |
| 2729.2154 | 98.862778 |  | 2729.2154 | 96.833239 |  | 2729.2154 | 97.957448 |
| 2731.0796 | 98.862161 |  | 2731.0796 | 96.817969 |  | 2731.0796 | 97.995791 |
| 2732.9438 | 98.895409 |  | 2732.9438 | 96.842792 |  | 2732.9438 | 97.967482 |
| 2734.808 | 98.881085 |  | 2734.808 | 96.834981 |  | 2734.808 | 97.943458 |
| 2736.6722 | 98.817666 |  | 2736.6722 | 96.776173 |  | 2736.6722 | 97.968183 |
| 2738.5365 | 98.845696 |  | 2738.5365 | 96.76264 |  | 2738.5365 | 97.952209 |
| 2740.4007 | 98.885699 |  | 2740.4007 | 96.783932 |  | 2740.4007 | 97.896234 |
| 2742.2649 | 98.87622 |  | 2742.2649 | 96.789432 |  | 2742.2649 | 97.912131 |
| 2744.1291 | 98.848049 |  | 2744.1291 | 96.801025 |  | 2744.1291 | 97.913314 |
| 2745.9933 | 98.859246 |  | 2745.9933 | 96.814247 |  | 2745.9933 | 97.902541 |
| 2747.8575 | 98.883363 |  | 2747.8575 | 96.81708 |  | 2747.8575 | 97.898809 |
| 2749.7218 | 98.842241 |  | 2749.7218 | 96.811364 |  | 2749.7218 | 97.87003 |
| 2751.586 | 98.80786 |  | 2751.586 | 96.781588 |  | 2751.586 | 97.850863 |
| 2753.4502 | 98.827673 |  | 2753.4502 | 96.74552 |  | 2753.4502 | 97.875304 |
| 2755.3144 | 98.812955 |  | 2755.3144 | 96.727238 |  | 2755.3144 | 97.85662 |
| 2757.1786 | 98.803984 |  | 2757.1786 | 96.709657 |  | 2757.1786 | 97.82273 |
| 2759.0428 | 98.845051 |  | 2759.0428 | 96.704033 |  | 2759.0428 | 97.841497 |
| 2760.9071 | 98.874944 |  | 2760.9071 | 96.717974 |  | 2760.9071 | 97.867442 |
| 2762.7713 | 98.877817 |  | 2762.7713 | 96.754888 |  | 2762.7713 | 97.844959 |
| 2764.6355 | 98.884531 |  | 2764.6355 | 96.781425 |  | 2764.6355 | 97.831697 |
| 2766.4997 | 98.883851 |  | 2766.4997 | 96.757282 |  | 2766.4997 | 97.830109 |
| 2768.3639 | 98.861749 |  | 2768.3639 | 96.712774 |  | 2768.3639 | 97.850154 |
| 2770.2282 | 98.85291 |  | 2770.2282 | 96.694137 |  | 2770.2282 | 97.845518 |
| 2772.0924 | 98.837141 |  | 2772.0924 | 96.676283 |  | 2772.0924 | 97.805549 |
| 2773.9566 | 98.804558 |  | 2773.9566 | 96.651088 |  | 2773.9566 | 97.7673 |
| 2775.8208 | 98.79904 |  | 2775.8208 | 96.625752 |  | 2775.8208 | 97.77354 |
| 2777.685 | 98.785486 |  | 2777.685 | 96.620474 |  | 2777.685 | 97.783966 |
| 2779.5492 | 98.788021 |  | 2779.5492 | 96.64816 |  | 2779.5492 | 97.796563 |
| 2781.4135 | 98.820406 |  | 2781.4135 | 96.669644 |  | 2781.4135 | 97.784068 |
| 2783.2777 | 98.827352 |  | 2783.2777 | 96.675236 |  | 2783.2777 | 97.781266 |
| 2785.1419 | 98.817473 |  | 2785.1419 | 96.656253 |  | 2785.1419 | 97.760114 |
| 2787.0061 | 98.866552 |  | 2787.0061 | 96.654886 |  | 2787.0061 | 97.751001 |
| 2788.8703 | 98.869325 |  | 2788.8703 | 96.656386 |  | 2788.8703 | 97.774664 |
| 2790.7346 | 98.822965 |  | 2790.7346 | 96.61622 |  | 2790.7346 | 97.749604 |
| 2792.5988 | 98.792812 |  | 2792.5988 | 96.581202 |  | 2792.5988 | 97.7149 |
| 2794.463 | 98.814522 |  | 2794.463 | 96.588814 |  | 2794.463 | 97.734534 |
| 2796.3272 | 98.826637 |  | 2796.3272 | 96.587048 |  | 2796.3272 | 97.72566 |
| 2798.1914 | 98.820121 |  | 2798.1914 | 96.581487 |  | 2798.1914 | 97.675501 |
| 2800.0556 | 98.831494 |  | 2800.0556 | 96.572419 |  | 2800.0556 | 97.648065 |
| 2801.9199 | 98.828811 |  | 2801.9199 | 96.524746 |  | 2801.9199 | 97.716496 |
| 2803.7841 | 98.794314 |  | 2803.7841 | 96.488745 |  | 2803.7841 | 97.773683 |
| 2805.6483 | 98.770654 |  | 2805.6483 | 96.490642 |  | 2805.6483 | 97.74477 |
| 2807.5125 | 98.767175 |  | 2807.5125 | 96.506314 |  | 2807.5125 | 97.692614 |
| 2809.3767 | 98.75978 |  | 2809.3767 | 96.491066 |  | 2809.3767 | 97.677357 |
| 2811.241 | 98.757873 |  | 2811.241 | 96.45758 |  | 2811.241 | 97.674446 |
| 2813.1052 | 98.771877 |  | 2813.1052 | 96.422112 |  | 2813.1052 | 97.674656 |
| 2814.9694 | 98.790407 |  | 2814.9694 | 96.405419 |  | 2814.9694 | 97.711408 |
| 2816.8336 | 98.790694 |  | 2816.8336 | 96.401144 |  | 2816.8336 | 97.780123 |
| 2818.6978 | 98.750775 |  | 2818.6978 | 96.385565 |  | 2818.6978 | 97.771322 |
| 2820.562 | 98.706228 |  | 2820.562 | 96.340234 |  | 2820.562 | 97.724094 |
| 2822.4263 | 98.697167 |  | 2822.4263 | 96.30024 |  | 2822.4263 | 97.661237 |
| 2824.2905 | 98.714226 |  | 2824.2905 | 96.288731 |  | 2824.2905 | 97.657211 |
| 2826.1547 | 98.758109 |  | 2826.1547 | 96.296946 |  | 2826.1547 | 97.67462 |
| 2828.0189 | 98.802735 |  | 2828.0189 | 96.312558 |  | 2828.0189 | 97.67783 |
| 2829.8831 | 98.781504 |  | 2829.8831 | 96.270066 |  | 2829.8831 | 97.642874 |
| 2831.7474 | 98.73973 |  | 2831.7474 | 96.20607 |  | 2831.7474 | 97.603586 |
| 2833.6116 | 98.707941 |  | 2833.6116 | 96.137211 |  | 2833.6116 | 97.555503 |
| 2835.4758 | 98.711914 |  | 2835.4758 | 96.084545 |  | 2835.4758 | 97.528842 |
| 2837.34 | 98.706964 |  | 2837.34 | 96.024736 |  | 2837.34 | 97.511861 |
| 2839.2042 | 98.685518 |  | 2839.2042 | 95.956312 |  | 2839.2042 | 97.483097 |
| 2841.0684 | 98.640536 |  | 2841.0684 | 95.848901 |  | 2841.0684 | 97.471012 |
| 2842.9327 | 98.631419 |  | 2842.9327 | 95.702203 |  | 2842.9327 | 97.521769 |
| 2844.7969 | 98.632569 |  | 2844.7969 | 95.584468 |  | 2844.7969 | 97.539052 |
| 2846.6611 | 98.620361 |  | 2846.6611 | 95.499592 |  | 2846.6611 | 97.525946 |
| 2848.5253 | 98.589523 |  | 2848.5253 | 95.342469 |  | 2848.5253 | 97.483803 |
| 2850.3895 | 98.560675 |  | 2850.3895 | 95.174103 |  | 2850.3895 | 97.455679 |
| 2852.2538 | 98.527076 |  | 2852.2538 | 95.114377 |  | 2852.2538 | 97.446078 |
| 2854.118 | 98.517979 |  | 2854.118 | 95.178538 |  | 2854.118 | 97.452903 |
| 2855.9822 | 98.539764 |  | 2855.9822 | 95.341459 |  | 2855.9822 | 97.471101 |
| 2857.8464 | 98.586785 |  | 2857.8464 | 95.498624 |  | 2857.8464 | 97.469649 |
| 2859.7106 | 98.611695 |  | 2859.7106 | 95.543072 |  | 2859.7106 | 97.428031 |
| 2861.5748 | 98.604356 |  | 2861.5748 | 95.569449 |  | 2861.5748 | 97.388312 |
| 2863.4391 | 98.567798 |  | 2863.4391 | 95.605763 |  | 2863.4391 | 97.368578 |
| 2865.3033 | 98.592808 |  | 2865.3033 | 95.680163 |  | 2865.3033 | 97.401597 |
| 2867.1675 | 98.601068 |  | 2867.1675 | 95.669382 |  | 2867.1675 | 97.402539 |
| 2869.0317 | 98.60315 |  | 2869.0317 | 95.631587 |  | 2869.0317 | 97.39245 |
| 2870.8959 | 98.631155 |  | 2870.8959 | 95.639498 |  | 2870.8959 | 97.383512 |
| 2872.7602 | 98.643947 |  | 2872.7602 | 95.611677 |  | 2872.7602 | 97.362187 |
| 2874.6244 | 98.615318 |  | 2874.6244 | 95.577295 |  | 2874.6244 | 97.284789 |
| 2876.4886 | 98.560901 |  | 2876.4886 | 95.558509 |  | 2876.4886 | 97.208864 |
| 2878.3528 | 98.595743 |  | 2878.3528 | 95.517881 |  | 2878.3528 | 97.178067 |
| 2880.217 | 98.64488 |  | 2880.217 | 95.513029 |  | 2880.217 | 97.226806 |
| 2882.0812 | 98.591944 |  | 2882.0812 | 95.507356 |  | 2882.0812 | 97.279038 |
| 2883.9455 | 98.579269 |  | 2883.9455 | 95.526493 |  | 2883.9455 | 97.294241 |
| 2885.8097 | 98.574831 |  | 2885.8097 | 95.548732 |  | 2885.8097 | 97.261792 |
| 2887.6739 | 98.543584 |  | 2887.6739 | 95.521588 |  | 2887.6739 | 97.239499 |
| 2889.5381 | 98.53824 |  | 2889.5381 | 95.458396 |  | 2889.5381 | 97.201388 |
| 2891.4023 | 98.559601 |  | 2891.4023 | 95.404675 |  | 2891.4023 | 97.139841 |
| 2893.2666 | 98.488177 |  | 2893.2666 | 95.329251 |  | 2893.2666 | 97.110014 |
| 2895.1308 | 98.493379 |  | 2895.1308 | 95.338399 |  | 2895.1308 | 97.132066 |
| 2896.995 | 98.598951 |  | 2896.995 | 95.363825 |  | 2896.995 | 97.191886 |
| 2898.8592 | 98.625692 |  | 2898.8592 | 95.30751 |  | 2898.8592 | 97.269635 |
| 2900.7234 | 98.578842 |  | 2900.7234 | 95.240568 |  | 2900.7234 | 97.37202 |
| 2902.5876 | 98.52721 |  | 2902.5876 | 95.217364 |  | 2902.5876 | 97.415555 |
| 2904.4519 | 98.490773 |  | 2904.4519 | 95.197027 |  | 2904.4519 | 97.392281 |
| 2906.3161 | 98.48687 |  | 2906.3161 | 95.126465 |  | 2906.3161 | 97.383279 |
| 2908.1803 | 98.46962 |  | 2908.1803 | 95.020884 |  | 2908.1803 | 97.343091 |
| 2910.0445 | 98.484785 |  | 2910.0445 | 94.968889 |  | 2910.0445 | 97.325071 |
| 2911.9087 | 98.508842 |  | 2911.9087 | 94.887673 |  | 2911.9087 | 97.190412 |
| 2913.773 | 98.511552 |  | 2913.773 | 94.713294 |  | 2913.773 | 96.997187 |
| 2915.6372 | 98.442636 |  | 2915.6372 | 94.488515 |  | 2915.6372 | 96.922109 |
| 2917.5014 | 98.415001 |  | 2917.5014 | 94.362366 |  | 2917.5014 | 97.034329 |
| 2919.3656 | 98.394845 |  | 2919.3656 | 94.305448 |  | 2919.3656 | 97.199166 |
| 2921.2298 | 98.335053 |  | 2921.2298 | 94.305805 |  | 2921.2298 | 97.371719 |
| 2923.094 | 98.361755 |  | 2923.094 | 94.413845 |  | 2923.094 | 97.473654 |
| 2924.9583 | 98.403626 |  | 2924.9583 | 94.537161 |  | 2924.9583 | 97.518095 |
| 2926.8225 | 98.423629 |  | 2926.8225 | 94.725988 |  | 2926.8225 | 97.551644 |
| 2928.6867 | 98.465346 |  | 2928.6867 | 94.912077 |  | 2928.6867 | 97.53287 |
| 2930.5509 | 98.479655 |  | 2930.5509 | 95.028378 |  | 2930.5509 | 97.473949 |
| 2932.4151 | 98.476843 |  | 2932.4151 | 95.116075 |  | 2932.4151 | 97.451403 |
| 2934.2794 | 98.484083 |  | 2934.2794 | 95.183555 |  | 2934.2794 | 97.405536 |
| 2936.1436 | 98.512536 |  | 2936.1436 | 95.300011 |  | 2936.1436 | 97.343509 |
| 2938.0078 | 98.543039 |  | 2938.0078 | 95.396068 |  | 2938.0078 | 97.279308 |
| 2939.872 | 98.632388 |  | 2939.872 | 95.494935 |  | 2939.872 | 97.176023 |
| 2941.7362 | 98.61968 |  | 2941.7362 | 95.529335 |  | 2941.7362 | 96.966672 |
| 2943.6004 | 98.61797 |  | 2943.6004 | 95.560051 |  | 2943.6004 | 96.821176 |
| 2945.4647 | 98.656764 |  | 2945.4647 | 95.603849 |  | 2945.4647 | 96.727983 |
| 2947.3289 | 98.627964 |  | 2947.3289 | 95.621954 |  | 2947.3289 | 96.743538 |
| 2949.1931 | 98.615772 |  | 2949.1931 | 95.652214 |  | 2949.1931 | 96.863525 |
| 2951.0573 | 98.60911 |  | 2951.0573 | 95.676035 |  | 2951.0573 | 97.1032 |
| 2952.9215 | 98.647215 |  | 2952.9215 | 95.720737 |  | 2952.9215 | 97.349408 |
| 2954.7858 | 98.656645 |  | 2954.7858 | 95.762023 |  | 2954.7858 | 97.547905 |
| 2956.65 | 98.657365 |  | 2956.65 | 95.779821 |  | 2956.65 | 97.67733 |
| 2958.5142 | 98.691131 |  | 2958.5142 | 95.780718 |  | 2958.5142 | 97.771677 |
| 2960.3784 | 98.654536 |  | 2960.3784 | 95.76168 |  | 2960.3784 | 97.786605 |
| 2962.2426 | 98.615807 |  | 2962.2426 | 95.81939 |  | 2962.2426 | 97.77468 |
| 2964.1068 | 98.648231 |  | 2964.1068 | 95.906477 |  | 2964.1068 | 97.780547 |
| 2965.9711 | 98.680986 |  | 2965.9711 | 95.952038 |  | 2965.9711 | 97.813332 |
| 2967.8353 | 98.661001 |  | 2967.8353 | 95.95389 |  | 2967.8353 | 97.820888 |
| 2969.6995 | 98.66706 |  | 2969.6995 | 96.032227 |  | 2969.6995 | 97.77704 |
| 2971.5637 | 98.719287 |  | 2971.5637 | 96.105823 |  | 2971.5637 | 97.739796 |
| 2973.4279 | 98.69179 |  | 2973.4279 | 96.065561 |  | 2973.4279 | 97.783417 |
| 2975.2922 | 98.737073 |  | 2975.2922 | 96.09561 |  | 2975.2922 | 97.764009 |
| 2977.1564 | 98.774114 |  | 2977.1564 | 96.128174 |  | 2977.1564 | 97.741399 |
| 2979.0206 | 98.691581 |  | 2979.0206 | 96.084196 |  | 2979.0206 | 97.746592 |
| 2980.8848 | 98.660068 |  | 2980.8848 | 96.085546 |  | 2980.8848 | 97.697689 |
| 2982.749 | 98.647454 |  | 2982.749 | 96.078968 |  | 2982.749 | 97.666031 |
| 2984.6132 | 98.670024 |  | 2984.6132 | 96.102582 |  | 2984.6132 | 97.723889 |
| 2986.4775 | 98.738641 |  | 2986.4775 | 96.204108 |  | 2986.4775 | 97.737319 |
| 2988.3417 | 98.754802 |  | 2988.3417 | 96.310801 |  | 2988.3417 | 97.719469 |
| 2990.2059 | 98.716603 |  | 2990.2059 | 96.280939 |  | 2990.2059 | 97.708409 |
| 2992.0701 | 98.711286 |  | 2992.0701 | 96.240848 |  | 2992.0701 | 97.718001 |
| 2993.9343 | 98.681935 |  | 2993.9343 | 96.213057 |  | 2993.9343 | 97.683687 |
| 2995.7986 | 98.680639 |  | 2995.7986 | 96.18499 |  | 2995.7986 | 97.661236 |
| 2997.6628 | 98.74552 |  | 2997.6628 | 96.226409 |  | 2997.6628 | 97.635379 |
| 2999.527 | 98.792716 |  | 2999.527 | 96.276638 |  | 2999.527 | 97.62047 |
| 3001.3912 | 98.781404 |  | 3001.3912 | 96.231776 |  | 3001.3912 | 97.628568 |
| 3003.2554 | 98.796094 |  | 3003.2554 | 96.1949 |  | 3003.2554 | 97.708842 |
| 3005.1196 | 98.814933 |  | 3005.1196 | 96.244716 |  | 3005.1196 | 97.689309 |
| 3006.9839 | 98.802202 |  | 3006.9839 | 96.267727 |  | 3006.9839 | 97.625036 |
| 3008.8481 | 98.753853 |  | 3008.8481 | 96.195551 |  | 3008.8481 | 97.611597 |
| 3010.7123 | 98.770414 |  | 3010.7123 | 96.200664 |  | 3010.7123 | 97.620861 |
| 3012.5765 | 98.792205 |  | 3012.5765 | 96.237239 |  | 3012.5765 | 97.582025 |
| 3014.4407 | 98.764361 |  | 3014.4407 | 96.192111 |  | 3014.4407 | 97.537874 |
| 3016.305 | 98.733755 |  | 3016.305 | 96.13142 |  | 3016.305 | 97.46317 |
| 3018.1692 | 98.706987 |  | 3018.1692 | 96.096846 |  | 3018.1692 | 97.466493 |
| 3020.0334 | 98.696839 |  | 3020.0334 | 96.054839 |  | 3020.0334 | 97.476552 |
| 3021.8976 | 98.728934 |  | 3021.8976 | 96.060633 |  | 3021.8976 | 97.489157 |
| 3023.7618 | 98.744133 |  | 3023.7618 | 96.093193 |  | 3023.7618 | 97.52421 |
| 3025.626 | 98.769985 |  | 3025.626 | 96.128767 |  | 3025.626 | 97.507562 |
| 3027.4903 | 98.728494 |  | 3027.4903 | 96.083724 |  | 3027.4903 | 97.437526 |
| 3029.3545 | 98.674701 |  | 3029.3545 | 96.01028 |  | 3029.3545 | 97.484427 |
| 3031.2187 | 98.659534 |  | 3031.2187 | 95.989759 |  | 3031.2187 | 97.516428 |
| 3033.0829 | 98.676672 |  | 3033.0829 | 96.025565 |  | 3033.0829 | 97.466792 |
| 3034.9471 | 98.66841 |  | 3034.9471 | 96.021358 |  | 3034.9471 | 97.317382 |
| 3036.8114 | 98.652353 |  | 3036.8114 | 96.003119 |  | 3036.8114 | 97.264152 |
| 3038.6756 | 98.671785 |  | 3038.6756 | 96.008212 |  | 3038.6756 | 97.306863 |
| 3040.5398 | 98.707586 |  | 3040.5398 | 96.003564 |  | 3040.5398 | 97.365329 |
| 3042.404 | 98.757886 |  | 3042.404 | 96.013656 |  | 3042.404 | 97.381644 |
| 3044.2682 | 98.712469 |  | 3044.2682 | 95.960147 |  | 3044.2682 | 97.347147 |
| 3046.1324 | 98.679222 |  | 3046.1324 | 95.915439 |  | 3046.1324 | 97.285592 |
| 3047.9967 | 98.70783 |  | 3047.9967 | 95.914627 |  | 3047.9967 | 97.296893 |
| 3049.8609 | 98.723147 |  | 3049.8609 | 95.897684 |  | 3049.8609 | 97.347785 |
| 3051.7251 | 98.780405 |  | 3051.7251 | 95.922786 |  | 3051.7251 | 97.28808 |
| 3053.5893 | 98.717771 |  | 3053.5893 | 95.88809 |  | 3053.5893 | 97.166281 |
| 3055.4535 | 98.682323 |  | 3055.4535 | 95.856925 |  | 3055.4535 | 97.147535 |
| 3057.3178 | 98.726081 |  | 3057.3178 | 95.821245 |  | 3057.3178 | 97.131137 |
| 3059.182 | 98.696468 |  | 3059.182 | 95.763649 |  | 3059.182 | 97.126043 |
| 3061.0462 | 98.67661 |  | 3061.0462 | 95.777082 |  | 3061.0462 | 97.122219 |
| 3062.9104 | 98.708244 |  | 3062.9104 | 95.805014 |  | 3062.9104 | 97.100366 |
| 3064.7746 | 98.683901 |  | 3064.7746 | 95.832618 |  | 3064.7746 | 97.088077 |
| 3066.6388 | 98.66967 |  | 3066.6388 | 95.83376 |  | 3066.6388 | 97.113425 |
| 3068.5031 | 98.67381 |  | 3068.5031 | 95.782244 |  | 3068.5031 | 97.040495 |
| 3070.3673 | 98.6466 |  | 3070.3673 | 95.708406 |  | 3070.3673 | 96.916924 |
| 3072.2315 | 98.672924 |  | 3072.2315 | 95.661383 |  | 3072.2315 | 96.93107 |
| 3074.0957 | 98.692402 |  | 3074.0957 | 95.671549 |  | 3074.0957 | 97.037761 |
| 3075.9599 | 98.640475 |  | 3075.9599 | 95.688449 |  | 3075.9599 | 97.053558 |
| 3077.8242 | 98.621909 |  | 3077.8242 | 95.697249 |  | 3077.8242 | 97.053113 |
| 3079.6884 | 98.653109 |  | 3079.6884 | 95.74971 |  | 3079.6884 | 96.984818 |
| 3081.5526 | 98.621629 |  | 3081.5526 | 95.755983 |  | 3081.5526 | 96.922014 |
| 3083.4168 | 98.572736 |  | 3083.4168 | 95.68207 |  | 3083.4168 | 96.833826 |
| 3085.281 | 98.550869 |  | 3085.281 | 95.597357 |  | 3085.281 | 96.821903 |
| 3087.1452 | 98.575635 |  | 3087.1452 | 95.539402 |  | 3087.1452 | 96.775489 |
| 3089.0095 | 98.648852 |  | 3089.0095 | 95.542849 |  | 3089.0095 | 96.753267 |
| 3090.8737 | 98.622197 |  | 3090.8737 | 95.516266 |  | 3090.8737 | 96.77088 |
| 3092.7379 | 98.569742 |  | 3092.7379 | 95.54103 |  | 3092.7379 | 96.798832 |
| 3094.6021 | 98.651857 |  | 3094.6021 | 95.635825 |  | 3094.6021 | 96.72938 |
| 3096.4663 | 98.640702 |  | 3096.4663 | 95.65554 |  | 3096.4663 | 96.725545 |
| 3098.3306 | 98.578496 |  | 3098.3306 | 95.610072 |  | 3098.3306 | 96.712252 |
| 3100.1948 | 98.567632 |  | 3100.1948 | 95.578357 |  | 3100.1948 | 96.661506 |
| 3102.059 | 98.542561 |  | 3102.059 | 95.52468 |  | 3102.059 | 96.668658 |
| 3103.9232 | 98.600323 |  | 3103.9232 | 95.493714 |  | 3103.9232 | 96.649502 |
| 3105.7874 | 98.617032 |  | 3105.7874 | 95.514965 |  | 3105.7874 | 96.57456 |
| 3107.6516 | 98.644462 |  | 3107.6516 | 95.600793 |  | 3107.6516 | 96.578162 |
| 3109.5159 | 98.650164 |  | 3109.5159 | 95.606171 |  | 3109.5159 | 96.582139 |
| 3111.3801 | 98.590822 |  | 3111.3801 | 95.491774 |  | 3111.3801 | 96.552299 |
| 3113.2443 | 98.595058 |  | 3113.2443 | 95.435309 |  | 3113.2443 | 96.54979 |
| 3115.1085 | 98.593528 |  | 3115.1085 | 95.448645 |  | 3115.1085 | 96.558651 |
| 3116.9727 | 98.594483 |  | 3116.9727 | 95.437742 |  | 3116.9727 | 96.521369 |
| 3118.837 | 98.556364 |  | 3118.837 | 95.378763 |  | 3118.837 | 96.460914 |
| 3120.7012 | 98.492106 |  | 3120.7012 | 95.298337 |  | 3120.7012 | 96.346657 |
| 3122.5654 | 98.531438 |  | 3122.5654 | 95.268359 |  | 3122.5654 | 96.308139 |
| 3124.4296 | 98.564539 |  | 3124.4296 | 95.294625 |  | 3124.4296 | 96.35912 |
| 3126.2938 | 98.525242 |  | 3126.2938 | 95.309527 |  | 3126.2938 | 96.388162 |
| 3128.158 | 98.54591 |  | 3128.158 | 95.323032 |  | 3128.158 | 96.33263 |
| 3130.0223 | 98.552768 |  | 3130.0223 | 95.292717 |  | 3130.0223 | 96.303142 |
| 3131.8865 | 98.545189 |  | 3131.8865 | 95.247439 |  | 3131.8865 | 96.216845 |
| 3133.7507 | 98.587519 |  | 3133.7507 | 95.305027 |  | 3133.7507 | 96.259936 |
| 3135.6149 | 98.561966 |  | 3135.6149 | 95.313368 |  | 3135.6149 | 96.248878 |
| 3137.4791 | 98.513099 |  | 3137.4791 | 95.209761 |  | 3137.4791 | 96.127452 |
| 3139.3434 | 98.530634 |  | 3139.3434 | 95.156662 |  | 3139.3434 | 96.089709 |
| 3141.2076 | 98.53093 |  | 3141.2076 | 95.190778 |  | 3141.2076 | 96.165219 |
| 3143.0718 | 98.501259 |  | 3143.0718 | 95.166833 |  | 3143.0718 | 96.144516 |
| 3144.936 | 98.656117 |  | 3144.936 | 95.180813 |  | 3144.936 | 96.134166 |
| 3146.8002 | 98.672504 |  | 3146.8002 | 95.188425 |  | 3146.8002 | 96.093201 |
| 3148.6644 | 98.541716 |  | 3148.6644 | 95.144538 |  | 3148.6644 | 96.104815 |
| 3150.5287 | 98.47879 |  | 3150.5287 | 95.065612 |  | 3150.5287 | 96.079438 |
| 3152.3929 | 98.50949 |  | 3152.3929 | 95.064835 |  | 3152.3929 | 96.015549 |
| 3154.2571 | 98.515935 |  | 3154.2571 | 95.038325 |  | 3154.2571 | 95.993511 |
| 3156.1213 | 98.503831 |  | 3156.1213 | 95.00791 |  | 3156.1213 | 96.016657 |
| 3157.9855 | 98.468993 |  | 3157.9855 | 94.977803 |  | 3157.9855 | 96.001969 |
| 3159.8498 | 98.480974 |  | 3159.8498 | 94.989792 |  | 3159.8498 | 95.980207 |
| 3161.714 | 98.51407 |  | 3161.714 | 95.031723 |  | 3161.714 | 95.920693 |
| 3163.5782 | 98.541155 |  | 3163.5782 | 94.987309 |  | 3163.5782 | 95.934343 |
| 3165.4424 | 98.546673 |  | 3165.4424 | 94.891696 |  | 3165.4424 | 95.947916 |
| 3167.3066 | 98.538392 |  | 3167.3066 | 94.919216 |  | 3167.3066 | 95.854135 |
| 3169.1708 | 98.48592 |  | 3169.1708 | 94.896066 |  | 3169.1708 | 95.774312 |
| 3171.0351 | 98.434977 |  | 3171.0351 | 94.866031 |  | 3171.0351 | 95.792043 |
| 3172.8993 | 98.414272 |  | 3172.8993 | 94.788623 |  | 3172.8993 | 95.7659 |
| 3174.7635 | 98.430825 |  | 3174.7635 | 94.711656 |  | 3174.7635 | 95.755376 |
| 3176.6277 | 98.409686 |  | 3176.6277 | 94.725139 |  | 3176.6277 | 95.80017 |
| 3178.4919 | 98.374115 |  | 3178.4919 | 94.744304 |  | 3178.4919 | 95.898202 |
| 3180.3561 | 98.361794 |  | 3180.3561 | 94.678343 |  | 3180.3561 | 95.874058 |
| 3182.2204 | 98.359765 |  | 3182.2204 | 94.616032 |  | 3182.2204 | 95.750699 |
| 3184.0846 | 98.416705 |  | 3184.0846 | 94.687647 |  | 3184.0846 | 95.671515 |
| 3185.9488 | 98.407983 |  | 3185.9488 | 94.689749 |  | 3185.9488 | 95.706056 |
| 3187.813 | 98.387599 |  | 3187.813 | 94.653439 |  | 3187.813 | 95.675037 |
| 3189.6772 | 98.4079 |  | 3189.6772 | 94.704226 |  | 3189.6772 | 95.651484 |
| 3191.5415 | 98.417998 |  | 3191.5415 | 94.662688 |  | 3191.5415 | 95.608916 |
| 3193.4057 | 98.406457 |  | 3193.4057 | 94.590458 |  | 3193.4057 | 95.642789 |
| 3195.2699 | 98.378951 |  | 3195.2699 | 94.568525 |  | 3195.2699 | 95.652673 |
| 3197.1341 | 98.397785 |  | 3197.1341 | 94.557618 |  | 3197.1341 | 95.650745 |
| 3198.9983 | 98.360537 |  | 3198.9983 | 94.494611 |  | 3198.9983 | 95.630727 |
| 3200.8625 | 98.306053 |  | 3200.8625 | 94.454545 |  | 3200.8625 | 95.659769 |
| 3202.7268 | 98.23714 |  | 3202.7268 | 94.399779 |  | 3202.7268 | 95.613882 |
| 3204.591 | 98.242689 |  | 3204.591 | 94.435259 |  | 3204.591 | 95.572279 |
| 3206.4552 | 98.394869 |  | 3206.4552 | 94.518574 |  | 3206.4552 | 95.584559 |
| 3208.3194 | 98.439731 |  | 3208.3194 | 94.498196 |  | 3208.3194 | 95.590676 |
| 3210.1836 | 98.355319 |  | 3210.1836 | 94.432712 |  | 3210.1836 | 95.513654 |
| 3212.0479 | 98.300089 |  | 3212.0479 | 94.383112 |  | 3212.0479 | 95.611019 |
| 3213.9121 | 98.262248 |  | 3213.9121 | 94.342432 |  | 3213.9121 | 95.624075 |
| 3215.7763 | 98.291708 |  | 3215.7763 | 94.281562 |  | 3215.7763 | 95.578775 |
| 3217.6405 | 98.376126 |  | 3217.6405 | 94.312811 |  | 3217.6405 | 95.555261 |
| 3219.5047 | 98.297666 |  | 3219.5047 | 94.266832 |  | 3219.5047 | 95.561552 |
| 3221.3689 | 98.265166 |  | 3221.3689 | 94.252503 |  | 3221.3689 | 95.550522 |
| 3223.2332 | 98.349942 |  | 3223.2332 | 94.300037 |  | 3223.2332 | 95.513119 |
| 3225.0974 | 98.331201 |  | 3225.0974 | 94.196773 |  | 3225.0974 | 95.534532 |
| 3226.9616 | 98.337743 |  | 3226.9616 | 94.186299 |  | 3226.9616 | 95.65504 |
| 3228.8258 | 98.403916 |  | 3228.8258 | 94.232299 |  | 3228.8258 | 95.74161 |
| 3230.69 | 98.385305 |  | 3230.69 | 94.235323 |  | 3230.69 | 95.645452 |
| 3232.5543 | 98.281132 |  | 3232.5543 | 94.150039 |  | 3232.5543 | 95.43434 |
| 3234.4185 | 98.163816 |  | 3234.4185 | 94.064263 |  | 3234.4185 | 95.447128 |
| 3236.2827 | 98.229922 |  | 3236.2827 | 94.051034 |  | 3236.2827 | 95.520142 |
| 3238.1469 | 98.280387 |  | 3238.1469 | 94.022381 |  | 3238.1469 | 95.531218 |
| 3240.0111 | 98.211441 |  | 3240.0111 | 94.001984 |  | 3240.0111 | 95.496941 |
| 3241.8753 | 98.234174 |  | 3241.8753 | 94.096178 |  | 3241.8753 | 95.534546 |
| 3243.7396 | 98.25049 |  | 3243.7396 | 94.111275 |  | 3243.7396 | 95.533073 |
| 3245.6038 | 98.190208 |  | 3245.6038 | 93.992045 |  | 3245.6038 | 95.586462 |
| 3247.468 | 98.16232 |  | 3247.468 | 93.940984 |  | 3247.468 | 95.577287 |
| 3249.3322 | 98.241463 |  | 3249.3322 | 93.998589 |  | 3249.3322 | 95.471609 |
| 3251.1964 | 98.23442 |  | 3251.1964 | 93.983456 |  | 3251.1964 | 95.470353 |
| 3253.0607 | 98.217084 |  | 3253.0607 | 94.028585 |  | 3253.0607 | 95.643163 |
| 3254.9249 | 98.260444 |  | 3254.9249 | 94.052769 |  | 3254.9249 | 95.619797 |
| 3256.7891 | 98.246154 |  | 3256.7891 | 93.970042 |  | 3256.7891 | 95.502904 |
| 3258.6533 | 98.193598 |  | 3258.6533 | 93.815443 |  | 3258.6533 | 95.440684 |
| 3260.5175 | 98.115607 |  | 3260.5175 | 93.735584 |  | 3260.5175 | 95.525725 |
| 3262.3817 | 98.122971 |  | 3262.3817 | 93.815432 |  | 3262.3817 | 95.592104 |
| 3264.246 | 98.174283 |  | 3264.246 | 93.883388 |  | 3264.246 | 95.572863 |
| 3266.1102 | 98.207843 |  | 3266.1102 | 93.829888 |  | 3266.1102 | 95.57231 |
| 3267.9744 | 98.177651 |  | 3267.9744 | 93.743507 |  | 3267.9744 | 95.533785 |
| 3269.8386 | 98.212121 |  | 3269.8386 | 93.783283 |  | 3269.8386 | 95.504536 |
| 3271.7028 | 98.260697 |  | 3271.7028 | 93.929012 |  | 3271.7028 | 95.572436 |
| 3273.5671 | 98.156161 |  | 3273.5671 | 93.878106 |  | 3273.5671 | 95.50043 |
| 3275.4313 | 98.164362 |  | 3275.4313 | 93.834375 |  | 3275.4313 | 95.576536 |
| 3277.2955 | 98.154503 |  | 3277.2955 | 93.816092 |  | 3277.2955 | 95.618239 |
| 3279.1597 | 98.129061 |  | 3279.1597 | 93.818282 |  | 3279.1597 | 95.520653 |
| 3281.0239 | 98.150967 |  | 3281.0239 | 93.816982 |  | 3281.0239 | 95.585035 |
| 3282.8881 | 98.141461 |  | 3282.8881 | 93.788335 |  | 3282.8881 | 95.700298 |
| 3284.7524 | 98.161438 |  | 3284.7524 | 93.748654 |  | 3284.7524 | 95.715861 |
| 3286.6166 | 98.188173 |  | 3286.6166 | 93.721215 |  | 3286.6166 | 95.716537 |
| 3288.4808 | 98.16879 |  | 3288.4808 | 93.724299 |  | 3288.4808 | 95.689488 |
| 3290.345 | 98.215638 |  | 3290.345 | 93.787435 |  | 3290.345 | 95.713234 |
| 3292.2092 | 98.199471 |  | 3292.2092 | 93.791692 |  | 3292.2092 | 95.660611 |
| 3294.0735 | 98.150379 |  | 3294.0735 | 93.83251 |  | 3294.0735 | 95.622101 |
| 3295.9377 | 98.146246 |  | 3295.9377 | 93.852427 |  | 3295.9377 | 95.635974 |
| 3297.8019 | 98.218703 |  | 3297.8019 | 93.828423 |  | 3297.8019 | 95.676002 |
| 3299.6661 | 98.174985 |  | 3299.6661 | 93.832508 |  | 3299.6661 | 95.707589 |
| 3301.5303 | 98.097164 |  | 3301.5303 | 93.832302 |  | 3301.5303 | 95.708658 |
| 3303.3945 | 98.146192 |  | 3303.3945 | 93.793742 |  | 3303.3945 | 95.631469 |
| 3305.2588 | 98.185267 |  | 3305.2588 | 93.821599 |  | 3305.2588 | 95.682531 |
| 3307.123 | 98.150887 |  | 3307.123 | 93.798766 |  | 3307.123 | 95.742526 |
| 3308.9872 | 98.122542 |  | 3308.9872 | 93.767026 |  | 3308.9872 | 95.824097 |
| 3310.8514 | 98.084873 |  | 3310.8514 | 93.792363 |  | 3310.8514 | 95.849269 |
| 3312.7156 | 98.086462 |  | 3312.7156 | 93.82161 |  | 3312.7156 | 95.717803 |
| 3314.5799 | 98.185902 |  | 3314.5799 | 93.886872 |  | 3314.5799 | 95.725694 |
| 3316.4441 | 98.251318 |  | 3316.4441 | 93.920273 |  | 3316.4441 | 95.842475 |
| 3318.3083 | 98.234661 |  | 3318.3083 | 93.91945 |  | 3318.3083 | 95.846556 |
| 3320.1725 | 98.283374 |  | 3320.1725 | 93.944964 |  | 3320.1725 | 95.872863 |
| 3322.0367 | 98.283846 |  | 3322.0367 | 93.901274 |  | 3322.0367 | 95.850303 |
| 3323.9009 | 98.229216 |  | 3323.9009 | 93.879572 |  | 3323.9009 | 95.96997 |
| 3325.7652 | 98.178885 |  | 3325.7652 | 93.868412 |  | 3325.7652 | 95.940983 |
| 3327.6294 | 98.088992 |  | 3327.6294 | 93.850414 |  | 3327.6294 | 95.963371 |
| 3329.4936 | 98.067452 |  | 3329.4936 | 93.884288 |  | 3329.4936 | 95.977025 |
| 3331.3578 | 98.173158 |  | 3331.3578 | 93.876509 |  | 3331.3578 | 95.987533 |
| 3333.222 | 98.239168 |  | 3333.222 | 93.854146 |  | 3333.222 | 96.079435 |
| 3335.0863 | 98.258782 |  | 3335.0863 | 93.89972 |  | 3335.0863 | 96.209702 |
| 3336.9505 | 98.198849 |  | 3336.9505 | 93.856118 |  | 3336.9505 | 96.269055 |
| 3338.8147 | 98.177875 |  | 3338.8147 | 93.856805 |  | 3338.8147 | 96.229579 |
| 3340.6789 | 98.142948 |  | 3340.6789 | 93.931544 |  | 3340.6789 | 96.113343 |
| 3342.5431 | 98.131062 |  | 3342.5431 | 93.979253 |  | 3342.5431 | 96.128731 |
| 3344.4073 | 98.234562 |  | 3344.4073 | 93.942512 |  | 3344.4073 | 96.140433 |
| 3346.2716 | 98.274429 |  | 3346.2716 | 93.867539 |  | 3346.2716 | 96.108497 |
| 3348.1358 | 98.219744 |  | 3348.1358 | 93.945417 |  | 3348.1358 | 96.217501 |
| 3350 | 98.274012 |  | 3350 | 94.136066 |  | 3350 | 96.290316 |
| 3351.8642 | 98.22018 |  | 3351.8642 | 94.064442 |  | 3351.8642 | 96.224833 |
| 3353.7284 | 98.17218 |  | 3353.7284 | 93.993611 |  | 3353.7284 | 96.251462 |
| 3355.5927 | 98.230965 |  | 3355.5927 | 94.046361 |  | 3355.5927 | 96.304257 |
| 3357.4569 | 98.290551 |  | 3357.4569 | 94.078916 |  | 3357.4569 | 96.415908 |
| 3359.3211 | 98.259792 |  | 3359.3211 | 94.06774 |  | 3359.3211 | 96.472075 |
| 3361.1853 | 98.178828 |  | 3361.1853 | 94.133941 |  | 3361.1853 | 96.488434 |
| 3363.0495 | 98.155948 |  | 3363.0495 | 94.110101 |  | 3363.0495 | 96.440767 |
| 3364.9137 | 98.209145 |  | 3364.9137 | 94.062859 |  | 3364.9137 | 96.423609 |
| 3366.778 | 98.292571 |  | 3366.778 | 94.181197 |  | 3366.778 | 96.404457 |
| 3368.6422 | 98.281933 |  | 3368.6422 | 94.254227 |  | 3368.6422 | 96.498421 |
| 3370.5064 | 98.20522 |  | 3370.5064 | 94.266152 |  | 3370.5064 | 96.465399 |
| 3372.3706 | 98.26253 |  | 3372.3706 | 94.306669 |  | 3372.3706 | 96.389796 |
| 3374.2348 | 98.292297 |  | 3374.2348 | 94.244477 |  | 3374.2348 | 96.451264 |
| 3376.0991 | 98.323136 |  | 3376.0991 | 94.317238 |  | 3376.0991 | 96.558833 |
| 3377.9633 | 98.333578 |  | 3377.9633 | 94.429091 |  | 3377.9633 | 96.652715 |
| 3379.8275 | 98.290579 |  | 3379.8275 | 94.417509 |  | 3379.8275 | 96.854039 |
| 3381.6917 | 98.326897 |  | 3381.6917 | 94.401636 |  | 3381.6917 | 96.934297 |
| 3383.5559 | 98.303562 |  | 3383.5559 | 94.248275 |  | 3383.5559 | 96.791861 |
| 3385.4201 | 98.286233 |  | 3385.4201 | 94.276455 |  | 3385.4201 | 96.602966 |
| 3387.2844 | 98.344728 |  | 3387.2844 | 94.437174 |  | 3387.2844 | 96.54558 |
| 3389.1486 | 98.433391 |  | 3389.1486 | 94.517162 |  | 3389.1486 | 96.514944 |
| 3391.0128 | 98.530733 |  | 3391.0128 | 94.509895 |  | 3391.0128 | 96.583341 |
| 3392.877 | 98.477567 |  | 3392.877 | 94.522743 |  | 3392.877 | 96.591188 |
| 3394.7412 | 98.404608 |  | 3394.7412 | 94.635496 |  | 3394.7412 | 96.663074 |
| 3396.6055 | 98.356529 |  | 3396.6055 | 94.570025 |  | 3396.6055 | 96.569267 |
| 3398.4697 | 98.335179 |  | 3398.4697 | 94.376644 |  | 3398.4697 | 96.398372 |
| 3400.3339 | 98.293379 |  | 3400.3339 | 94.365475 |  | 3400.3339 | 96.378147 |
| 3402.1981 | 98.216345 |  | 3402.1981 | 94.460836 |  | 3402.1981 | 96.303867 |
| 3404.0623 | 98.344652 |  | 3404.0623 | 94.610841 |  | 3404.0623 | 96.408699 |
| 3405.9265 | 98.493864 |  | 3405.9265 | 94.675726 |  | 3405.9265 | 96.59346 |
| 3407.7908 | 98.42165 |  | 3407.7908 | 94.665101 |  | 3407.7908 | 96.560223 |
| 3409.655 | 98.38357 |  | 3409.655 | 94.70091 |  | 3409.655 | 96.597397 |
| 3411.5192 | 98.456926 |  | 3411.5192 | 94.770505 |  | 3411.5192 | 96.643675 |
| 3413.3834 | 98.455749 |  | 3413.3834 | 94.767112 |  | 3413.3834 | 96.681044 |
| 3415.2476 | 98.347294 |  | 3415.2476 | 94.690724 |  | 3415.2476 | 96.697618 |
| 3417.1119 | 98.319499 |  | 3417.1119 | 94.774907 |  | 3417.1119 | 96.802159 |
| 3418.9761 | 98.424851 |  | 3418.9761 | 94.831188 |  | 3418.9761 | 97.025367 |
| 3420.8403 | 98.466896 |  | 3420.8403 | 94.750018 |  | 3420.8403 | 97.250933 |
| 3422.7045 | 98.405849 |  | 3422.7045 | 94.685638 |  | 3422.7045 | 97.326146 |
| 3424.5687 | 98.360948 |  | 3424.5687 | 94.833019 |  | 3424.5687 | 97.194591 |
| 3426.4329 | 98.414953 |  | 3426.4329 | 94.98288 |  | 3426.4329 | 97.100264 |
| 3428.2972 | 98.586577 |  | 3428.2972 | 94.935731 |  | 3428.2972 | 97.29113 |
| 3430.1614 | 98.541639 |  | 3430.1614 | 94.93128 |  | 3430.1614 | 97.506556 |
| 3432.0256 | 98.490689 |  | 3432.0256 | 95.027561 |  | 3432.0256 | 97.540704 |
| 3433.8898 | 98.401586 |  | 3433.8898 | 95.052931 |  | 3433.8898 | 97.483034 |
| 3435.754 | 98.35641 |  | 3435.754 | 95.030508 |  | 3435.754 | 97.579424 |
| 3437.6183 | 98.417117 |  | 3437.6183 | 95.099702 |  | 3437.6183 | 97.706874 |
| 3439.4825 | 98.449709 |  | 3439.4825 | 95.090553 |  | 3439.4825 | 97.689529 |
| 3441.3467 | 98.458012 |  | 3441.3467 | 95.079191 |  | 3441.3467 | 97.671149 |
| 3443.2109 | 98.469765 |  | 3443.2109 | 95.182772 |  | 3443.2109 | 97.950261 |
| 3445.0751 | 98.445025 |  | 3445.0751 | 95.20639 |  | 3445.0751 | 98.070244 |
| 3446.9393 | 98.425237 |  | 3446.9393 | 95.12747 |  | 3446.9393 | 97.863523 |
| 3448.8036 | 98.532802 |  | 3448.8036 | 95.179372 |  | 3448.8036 | 97.745838 |
| 3450.6678 | 98.557596 |  | 3450.6678 | 95.264063 |  | 3450.6678 | 97.586444 |
| 3452.532 | 98.432271 |  | 3452.532 | 95.254195 |  | 3452.532 | 97.454835 |
| 3454.3962 | 98.555256 |  | 3454.3962 | 95.307729 |  | 3454.3962 | 97.839394 |
| 3456.2604 | 98.7042 |  | 3456.2604 | 95.434046 |  | 3456.2604 | 98.024158 |
| 3458.1247 | 98.675615 |  | 3458.1247 | 95.49251 |  | 3458.1247 | 98.003275 |
| 3459.9889 | 98.520152 |  | 3459.9889 | 95.524492 |  | 3459.9889 | 98.040312 |
| 3461.8531 | 98.572932 |  | 3461.8531 | 95.679019 |  | 3461.8531 | 98.055284 |
| 3463.7173 | 98.718727 |  | 3463.7173 | 95.735342 |  | 3463.7173 | 98.053048 |
| 3465.5815 | 98.655857 |  | 3465.5815 | 95.706766 |  | 3465.5815 | 98.117027 |
| 3467.4457 | 98.591064 |  | 3467.4457 | 95.578087 |  | 3467.4457 | 97.988155 |
| 3469.31 | 98.599012 |  | 3469.31 | 95.517551 |  | 3469.31 | 97.88273 |
| 3471.1742 | 98.552744 |  | 3471.1742 | 95.610936 |  | 3471.1742 | 97.867203 |
| 3473.0384 | 98.589683 |  | 3473.0384 | 95.720739 |  | 3473.0384 | 97.878034 |
| 3474.9026 | 98.740971 |  | 3474.9026 | 95.846659 |  | 3474.9026 | 98.104504 |
| 3476.7668 | 98.808374 |  | 3476.7668 | 95.976876 |  | 3476.7668 | 98.308323 |
| 3478.6311 | 98.644399 |  | 3478.6311 | 95.946009 |  | 3478.6311 | 98.237289 |
| 3480.4953 | 98.516899 |  | 3480.4953 | 95.862796 |  | 3480.4953 | 98.142001 |
| 3482.3595 | 98.610806 |  | 3482.3595 | 95.894654 |  | 3482.3595 | 98.383268 |
| 3484.2237 | 98.734876 |  | 3484.2237 | 95.939733 |  | 3484.2237 | 98.447329 |
| 3486.0879 | 98.719591 |  | 3486.0879 | 95.936245 |  | 3486.0879 | 98.055863 |
| 3487.9521 | 98.716422 |  | 3487.9521 | 95.965087 |  | 3487.9521 | 97.963446 |
| 3489.8164 | 98.721817 |  | 3489.8164 | 96.02484 |  | 3489.8164 | 98.244905 |
| 3491.6806 | 98.631823 |  | 3491.6806 | 96.019408 |  | 3491.6806 | 98.146494 |
| 3493.5448 | 98.654752 |  | 3493.5448 | 96.132987 |  | 3493.5448 | 98.000448 |
| 3495.409 | 98.731155 |  | 3495.409 | 96.227312 |  | 3495.409 | 98.240861 |
| 3497.2732 | 98.744092 |  | 3497.2732 | 96.210786 |  | 3497.2732 | 98.418638 |
| 3499.1375 | 98.797135 |  | 3499.1375 | 96.258439 |  | 3499.1375 | 98.361793 |
| 3501.0017 | 98.722156 |  | 3501.0017 | 96.212304 |  | 3501.0017 | 98.302562 |
| 3502.8659 | 98.891567 |  | 3502.8659 | 96.381503 |  | 3502.8659 | 98.507455 |
| 3504.7301 | 99.106424 |  | 3504.7301 | 96.62106 |  | 3504.7301 | 98.569776 |
| 3506.5943 | 98.8038 |  | 3506.5943 | 96.446017 |  | 3506.5943 | 98.174983 |
| 3508.4585 | 98.481965 |  | 3508.4585 | 96.199569 |  | 3508.4585 | 97.909057 |
| 3510.3228 | 98.728194 |  | 3510.3228 | 96.288093 |  | 3510.3228 | 98.224425 |
| 3512.187 | 98.959307 |  | 3512.187 | 96.365543 |  | 3512.187 | 98.447708 |
| 3514.0512 | 98.801327 |  | 3514.0512 | 96.21435 |  | 3514.0512 | 98.051144 |
| 3515.9154 | 98.677105 |  | 3515.9154 | 96.177806 |  | 3515.9154 | 97.924575 |
| 3517.7796 | 98.707807 |  | 3517.7796 | 96.346244 |  | 3517.7796 | 98.187889 |
| 3519.6439 | 98.842656 |  | 3519.6439 | 96.506239 |  | 3519.6439 | 98.4217 |
| 3521.5081 | 98.985193 |  | 3521.5081 | 96.508648 |  | 3521.5081 | 98.74079 |
| 3523.3723 | 99.030844 |  | 3523.3723 | 96.60179 |  | 3523.3723 | 98.829761 |
| 3525.2365 | 98.988728 |  | 3525.2365 | 96.875661 |  | 3525.2365 | 98.842671 |
| 3527.1007 | 98.813838 |  | 3527.1007 | 96.83427 |  | 3527.1007 | 98.288309 |
| 3528.9649 | 98.707168 |  | 3528.9649 | 96.669722 |  | 3528.9649 | 97.770127 |
| 3530.8292 | 98.869802 |  | 3530.8292 | 96.773797 |  | 3530.8292 | 97.940875 |
| 3532.6934 | 98.649825 |  | 3532.6934 | 96.580304 |  | 3532.6934 | 97.813919 |
| 3534.5576 | 98.341113 |  | 3534.5576 | 96.275638 |  | 3534.5576 | 97.754161 |
| 3536.4218 | 98.436414 |  | 3536.4218 | 96.244502 |  | 3536.4218 | 98.15797 |
| 3538.286 | 98.703785 |  | 3538.286 | 96.43199 |  | 3538.286 | 98.544078 |
| 3540.1503 | 98.840661 |  | 3540.1503 | 96.674017 |  | 3540.1503 | 98.366454 |
| 3542.0145 | 98.815004 |  | 3542.0145 | 96.834169 |  | 3542.0145 | 98.153603 |
| 3543.8787 | 98.933242 |  | 3543.8787 | 96.91585 |  | 3543.8787 | 98.611955 |
| 3545.7429 | 99.021271 |  | 3545.7429 | 96.886765 |  | 3545.7429 | 98.8865 |
| 3547.6071 | 98.995534 |  | 3547.6071 | 96.919212 |  | 3547.6071 | 98.791989 |
| 3549.4713 | 98.625818 |  | 3549.4713 | 96.829912 |  | 3549.4713 | 98.085992 |
| 3551.3356 | 98.274471 |  | 3551.3356 | 96.640762 |  | 3551.3356 | 97.431005 |
| 3553.1998 | 98.445298 |  | 3553.1998 | 96.704143 |  | 3553.1998 | 97.884104 |
| 3555.064 | 98.817806 |  | 3555.064 | 96.880248 |  | 3555.064 | 98.370428 |
| 3556.9282 | 98.807606 |  | 3556.9282 | 96.958112 |  | 3556.9282 | 97.982203 |
| 3558.7924 | 98.6311 |  | 3558.7924 | 96.856763 |  | 3558.7924 | 97.948789 |
| 3560.6567 | 98.832061 |  | 3560.6567 | 96.932869 |  | 3560.6567 | 98.479908 |
| 3562.5209 | 99.007396 |  | 3562.5209 | 97.139778 |  | 3562.5209 | 98.798263 |
| 3564.3851 | 99.073985 |  | 3564.3851 | 97.254172 |  | 3564.3851 | 98.889545 |
| 3566.2493 | 99.199637 |  | 3566.2493 | 97.347209 |  | 3566.2493 | 99.002862 |
| 3568.1135 | 99.3095 |  | 3568.1135 | 97.598155 |  | 3568.1135 | 99.362895 |
| 3569.9777 | 98.856587 |  | 3569.9777 | 97.3604 |  | 3569.9777 | 98.451694 |
| 3571.842 | 97.674647 |  | 3571.842 | 96.539139 |  | 3571.842 | 96.146216 |
| 3573.7062 | 97.665365 |  | 3573.7062 | 96.608878 |  | 3573.7062 | 96.368483 |
| 3575.5704 | 98.545589 |  | 3575.5704 | 96.972979 |  | 3575.5704 | 97.90458 |
| 3577.4346 | 99.00819 |  | 3577.4346 | 97.129151 |  | 3577.4346 | 98.616559 |
| 3579.2988 | 98.921157 |  | 3579.2988 | 97.270855 |  | 3579.2988 | 98.462305 |
| 3581.1631 | 98.765711 |  | 3581.1631 | 97.287703 |  | 3581.1631 | 98.285199 |
| 3583.0273 | 98.774599 |  | 3583.0273 | 97.166811 |  | 3583.0273 | 98.385671 |
| 3584.8915 | 98.767478 |  | 3584.8915 | 97.200476 |  | 3584.8915 | 98.385058 |
| 3586.7557 | 98.92076 |  | 3586.7557 | 97.526114 |  | 3586.7557 | 98.618345 |
| 3588.6199 | 99.207021 |  | 3588.6199 | 97.839499 |  | 3588.6199 | 99.3073 |
| 3590.4841 | 98.903496 |  | 3590.4841 | 97.649112 |  | 3590.4841 | 98.836359 |
| 3592.3484 | 97.841972 |  | 3592.3484 | 96.892022 |  | 3592.3484 | 96.582307 |
| 3594.2126 | 97.920009 |  | 3594.2126 | 96.79792 |  | 3594.2126 | 96.729019 |
| 3596.0768 | 98.747177 |  | 3596.0768 | 97.209384 |  | 3596.0768 | 98.376507 |
| 3597.941 | 98.870027 |  | 3597.941 | 97.347857 |  | 3597.941 | 98.609903 |
| 3599.8052 | 98.477862 |  | 3599.8052 | 97.262283 |  | 3599.8052 | 97.750544 |
| 3601.6694 | 98.576123 |  | 3601.6694 | 97.343488 |  | 3601.6694 | 97.78456 |
| 3603.5337 | 98.773854 |  | 3603.5337 | 97.387935 |  | 3603.5337 | 98.09892 |
| 3605.3979 | 98.623964 |  | 3605.3979 | 97.26033 |  | 3605.3979 | 97.881505 |
| 3607.2621 | 98.725613 |  | 3607.2621 | 97.332778 |  | 3607.2621 | 98.192686 |
| 3609.1263 | 99.220332 |  | 3609.1263 | 97.686882 |  | 3609.1263 | 99.253011 |
| 3610.9905 | 99.340889 |  | 3610.9905 | 97.822508 |  | 3610.9905 | 99.415795 |
| 3612.8548 | 98.736591 |  | 3612.8548 | 97.566036 |  | 3612.8548 | 98.13201 |
| 3614.719 | 98.588763 |  | 3614.719 | 97.582012 |  | 3614.719 | 97.96972 |
| 3616.5832 | 98.765845 |  | 3616.5832 | 97.740101 |  | 3616.5832 | 98.255733 |
| 3618.4474 | 98.547825 |  | 3618.4474 | 97.567987 |  | 3618.4474 | 97.697948 |
| 3620.3116 | 98.811663 |  | 3620.3116 | 97.686291 |  | 3620.3116 | 98.205163 |
| 3622.1758 | 98.90739 |  | 3622.1758 | 97.777048 |  | 3622.1758 | 98.254775 |
| 3624.0401 | 98.16858 |  | 3624.0401 | 97.320959 |  | 3624.0401 | 96.67946 |
| 3625.9043 | 98.230983 |  | 3625.9043 | 97.297835 |  | 3625.9043 | 96.979579 |
| 3627.7685 | 98.94681 |  | 3627.7685 | 97.841802 |  | 3627.7685 | 98.656479 |
| 3629.6327 | 99.438526 |  | 3629.6327 | 98.259374 |  | 3629.6327 | 100.22993 |
| 3631.4969 | 99.491587 |  | 3631.4969 | 98.120715 |  | 3631.4969 | 100.22519 |
| 3633.3612 | 97.934316 |  | 3633.3612 | 97.048278 |  | 3633.3612 | 96.315607 |
| 3635.2254 | 97.418298 |  | 3635.2254 | 96.73917 |  | 3635.2254 | 95.514233 |
| 3637.0896 | 98.416387 |  | 3637.0896 | 97.324503 |  | 3637.0896 | 97.388673 |
| 3638.9538 | 98.678303 |  | 3638.9538 | 97.497233 |  | 3638.9538 | 97.893268 |
| 3640.818 | 98.76959 |  | 3640.818 | 97.537187 |  | 3640.818 | 98.230599 |
| 3642.6822 | 99.101777 |  | 3642.6822 | 97.827384 |  | 3642.6822 | 98.75555 |
| 3644.5465 | 99.241194 |  | 3644.5465 | 98.01711 |  | 3644.5465 | 98.814748 |
| 3646.4107 | 99.307645 |  | 3646.4107 | 98.131402 |  | 3646.4107 | 99.008765 |
| 3648.2749 | 99.513603 |  | 3648.2749 | 98.383479 |  | 3648.2749 | 99.9687 |
| 3650.1391 | 99.862932 |  | 3650.1391 | 98.701542 |  | 3650.1391 | 101.19772 |
| 3652.0033 | 98.961392 |  | 3652.0033 | 98.262811 |  | 3652.0033 | 99.145544 |
| 3653.8676 | 97.25763 |  | 3653.8676 | 97.248989 |  | 3653.8676 | 95.064484 |
| 3655.7318 | 97.172516 |  | 3655.7318 | 97.038459 |  | 3655.7318 | 94.846269 |
| 3657.596 | 98.403656 |  | 3657.596 | 97.58491 |  | 3657.596 | 97.309502 |
| 3659.4602 | 99.15187 |  | 3659.4602 | 97.91077 |  | 3659.4602 | 98.595829 |
| 3661.3244 | 98.504374 |  | 3661.3244 | 97.581816 |  | 3661.3244 | 97.360605 |
| 3663.1886 | 98.570022 |  | 3663.1886 | 97.654777 |  | 3663.1886 | 97.516135 |
| 3665.0529 | 99.150219 |  | 3665.0529 | 98.002015 |  | 3665.0529 | 98.486898 |
| 3666.9171 | 99.32326 |  | 3666.9171 | 98.155147 |  | 3666.9171 | 98.905197 |
| 3668.7813 | 99.509078 |  | 3668.7813 | 98.349362 |  | 3668.7813 | 99.465423 |
| 3670.6455 | 99.875992 |  | 3670.6455 | 98.681324 |  | 3670.6455 | 100.42184 |
| 3672.5097 | 100.00752 |  | 3672.5097 | 98.859305 |  | 3672.5097 | 100.61537 |
| 3674.374 | 98.583267 |  | 3674.374 | 98.129604 |  | 3674.374 | 97.625578 |
| 3676.2382 | 98.074402 |  | 3676.2382 | 97.851548 |  | 3676.2382 | 97.349096 |
| 3678.1024 | 99.24922 |  | 3678.1024 | 98.410459 |  | 3678.1024 | 99.356167 |
| 3679.9666 | 98.218492 |  | 3679.9666 | 97.737462 |  | 3679.9666 | 96.622724 |
| 3681.8308 | 97.286459 |  | 3681.8308 | 97.190439 |  | 3681.8308 | 95.344354 |
| 3683.695 | 98.244826 |  | 3683.695 | 97.616316 |  | 3683.695 | 97.047933 |
| 3685.5593 | 98.846962 |  | 3685.5593 | 97.87614 |  | 3685.5593 | 98.076141 |
| 3687.4235 | 99.210074 |  | 3687.4235 | 98.156876 |  | 3687.4235 | 98.992245 |
| 3689.2877 | 99.605259 |  | 3689.2877 | 98.48263 |  | 3689.2877 | 99.963495 |
| 3691.1519 | 99.858682 |  | 3691.1519 | 98.723643 |  | 3691.1519 | 100.31978 |
| 3693.0161 | 98.951735 |  | 3693.0161 | 98.303918 |  | 3693.0161 | 98.094464 |
| 3694.8804 | 97.671527 |  | 3694.8804 | 97.447974 |  | 3694.8804 | 95.872692 |
| 3696.7446 | 97.880573 |  | 3696.7446 | 97.404157 |  | 3696.7446 | 96.390469 |
| 3698.6088 | 98.690613 |  | 3698.6088 | 97.873046 |  | 3698.6088 | 97.74146 |
| 3700.473 | 99.137822 |  | 3700.473 | 98.213622 |  | 3700.473 | 98.500976 |
| 3702.3372 | 99.229914 |  | 3702.3372 | 98.252749 |  | 3702.3372 | 98.893807 |
| 3704.2014 | 99.31834 |  | 3704.2014 | 98.282968 |  | 3704.2014 | 99.273224 |
| 3706.0657 | 98.828055 |  | 3706.0657 | 98.061994 |  | 3706.0657 | 98.25947 |
| 3707.9299 | 98.544341 |  | 3707.9299 | 97.859515 |  | 3707.9299 | 97.653039 |
| 3709.7941 | 99.165988 |  | 3709.7941 | 98.211953 |  | 3709.7941 | 98.754313 |
| 3711.6583 | 99.650252 |  | 3711.6583 | 98.641281 |  | 3711.6583 | 99.888679 |
| 3713.5225 | 99.484638 |  | 3713.5225 | 98.65242 |  | 3713.5225 | 99.71856 |
| 3715.3868 | 98.483091 |  | 3715.3868 | 98.078303 |  | 3715.3868 | 97.34675 |
| 3717.251 | 97.609518 |  | 3717.251 | 97.490488 |  | 3717.251 | 95.770846 |
| 3719.1152 | 98.125377 |  | 3719.1152 | 97.660484 |  | 3719.1152 | 96.92289 |
| 3720.9794 | 99.068525 |  | 3720.9794 | 98.189817 |  | 3720.9794 | 98.548302 |
| 3722.8436 | 99.300753 |  | 3722.8436 | 98.39882 |  | 3722.8436 | 99.023675 |
| 3724.7078 | 99.050872 |  | 3724.7078 | 98.293916 |  | 3724.7078 | 98.94397 |
| 3726.5721 | 98.768153 |  | 3726.5721 | 98.121455 |  | 3726.5721 | 98.406962 |
| 3728.4363 | 98.617354 |  | 3728.4363 | 98.046787 |  | 3728.4363 | 97.842162 |
| 3730.3005 | 98.513852 |  | 3730.3005 | 98.032161 |  | 3730.3005 | 97.579628 |
| 3732.1647 | 98.589337 |  | 3732.1647 | 98.084387 |  | 3732.1647 | 97.871962 |
| 3734.0289 | 99.36431 |  | 3734.0289 | 98.457834 |  | 3734.0289 | 99.623502 |
| 3735.8932 | 99.818362 |  | 3735.8932 | 98.728017 |  | 3735.8932 | 100.55846 |
| 3737.7574 | 99.100121 |  | 3737.7574 | 98.575435 |  | 3737.7574 | 98.971488 |
| 3739.6216 | 98.015898 |  | 3739.6216 | 98.082375 |  | 3739.6216 | 96.538398 |
| 3741.4858 | 97.406353 |  | 3741.4858 | 97.519118 |  | 3741.4858 | 95.544568 |
| 3743.35 | 98.127353 |  | 3743.35 | 97.741507 |  | 3743.35 | 97.1695 |
| 3745.2142 | 99.185291 |  | 3745.2142 | 98.336027 |  | 3745.2142 | 99.340266 |
| 3747.0785 | 99.919479 |  | 3747.0785 | 98.851076 |  | 3747.0785 | 100.64286 |
| 3748.9427 | 98.685421 |  | 3748.9427 | 98.261392 |  | 3748.9427 | 97.718449 |
| 3750.8069 | 97.756379 |  | 3750.8069 | 97.773887 |  | 3750.8069 | 96.625538 |
| 3752.6711 | 98.783305 |  | 3752.6711 | 98.290405 |  | 3752.6711 | 98.711526 |
| 3754.5353 | 98.558546 |  | 3754.5353 | 98.124406 |  | 3754.5353 | 97.551244 |
| 3756.3996 | 97.234443 |  | 3756.3996 | 97.249977 |  | 3756.3996 | 95.102811 |
| 3758.2638 | 97.561937 |  | 3758.2638 | 97.304847 |  | 3758.2638 | 96.105283 |
| 3760.128 | 98.634487 |  | 3760.128 | 97.867856 |  | 3760.128 | 98.034019 |
| 3761.9922 | 99.073057 |  | 3761.9922 | 98.150456 |  | 3761.9922 | 98.565745 |
| 3763.8564 | 98.548324 |  | 3763.8564 | 97.876681 |  | 3763.8564 | 97.468908 |
| 3765.7206 | 98.356675 |  | 3765.7206 | 97.718654 |  | 3765.7206 | 97.31175 |
| 3767.5849 | 99.070627 |  | 3767.5849 | 98.096889 |  | 3767.5849 | 98.639211 |
| 3769.4491 | 99.184353 |  | 3769.4491 | 98.213586 |  | 3769.4491 | 98.747589 |
| 3771.3133 | 98.788883 |  | 3771.3133 | 98.00962 |  | 3771.3133 | 98.144173 |
| 3773.1775 | 98.926071 |  | 3773.1775 | 97.978213 |  | 3773.1775 | 98.136776 |
| 3775.0417 | 98.511116 |  | 3775.0417 | 97.691156 |  | 3775.0417 | 97.117915 |
| 3776.906 | 98.534531 |  | 3776.906 | 97.714903 |  | 3776.906 | 97.367745 |
| 3778.7702 | 99.098402 |  | 3778.7702 | 98.075902 |  | 3778.7702 | 98.522032 |
| 3780.6344 | 99.36113 |  | 3780.6344 | 98.290738 |  | 3780.6344 | 99.222033 |
| 3782.4986 | 99.362174 |  | 3782.4986 | 98.270957 |  | 3782.4986 | 99.002618 |
| 3784.3628 | 98.75092 |  | 3784.3628 | 97.829101 |  | 3784.3628 | 97.584944 |
| 3786.227 | 98.666218 |  | 3786.227 | 97.720273 |  | 3786.227 | 97.694114 |
| 3788.0913 | 99.077113 |  | 3788.0913 | 97.971993 |  | 3788.0913 | 98.39974 |
| 3789.9555 | 98.903934 |  | 3789.9555 | 97.956552 |  | 3789.9555 | 97.921304 |
| 3791.8197 | 98.873125 |  | 3791.8197 | 97.959971 |  | 3791.8197 | 97.970771 |
| 3793.6839 | 99.177439 |  | 3793.6839 | 98.111932 |  | 3793.6839 | 98.51312 |
| 3795.5481 | 99.326361 |  | 3795.5481 | 98.221859 |  | 3795.5481 | 98.78128 |
| 3797.4124 | 99.473336 |  | 3797.4124 | 98.363775 |  | 3797.4124 | 99.291022 |
| 3799.2766 | 99.588901 |  | 3799.2766 | 98.471935 |  | 3799.2766 | 99.793407 |
| 3801.1408 | 98.965722 |  | 3801.1408 | 98.13006 |  | 3801.1408 | 98.836068 |
| 3803.005 | 98.952841 |  | 3803.005 | 98.184783 |  | 3803.005 | 98.929056 |
| 3804.8692 | 99.311669 |  | 3804.8692 | 98.460408 |  | 3804.8692 | 98.929345 |
| 3806.7334 | 97.916308 |  | 3806.7334 | 97.626153 |  | 3806.7334 | 96.251581 |
| 3808.5977 | 98.107544 |  | 3808.5977 | 97.614502 |  | 3808.5977 | 97.123353 |
| 3810.4619 | 98.943479 |  | 3810.4619 | 98.003075 |  | 3810.4619 | 98.26355 |
| 3812.3261 | 98.434316 |  | 3812.3261 | 97.67368 |  | 3812.3261 | 96.896741 |
| 3814.1903 | 98.660274 |  | 3814.1903 | 97.79788 |  | 3814.1903 | 97.542885 |
| 3816.0545 | 99.410684 |  | 3816.0545 | 98.252293 |  | 3816.0545 | 99.162319 |
| 3817.9188 | 99.939554 |  | 3817.9188 | 98.604549 |  | 3817.9188 | 100.40528 |
| 3819.783 | 99.58776 |  | 3819.783 | 98.46697 |  | 3819.783 | 99.468242 |
| 3821.6472 | 98.368784 |  | 3821.6472 | 97.880741 |  | 3821.6472 | 97.533231 |
| 3823.5114 | 98.935734 |  | 3823.5114 | 98.223362 |  | 3823.5114 | 98.709453 |
| 3825.3756 | 98.592732 |  | 3825.3756 | 97.952203 |  | 3825.3756 | 97.241886 |
| 3827.2398 | 97.648395 |  | 3827.2398 | 97.250116 |  | 3827.2398 | 95.771869 |
| 3829.1041 | 98.604277 |  | 3829.1041 | 97.694227 |  | 3829.1041 | 97.7874 |
| 3830.9683 | 99.011761 |  | 3830.9683 | 98.016866 |  | 3830.9683 | 98.322559 |
| 3832.8325 | 98.874463 |  | 3832.8325 | 98.083898 |  | 3832.8325 | 98.232914 |
| 3834.6967 | 99.320518 |  | 3834.6967 | 98.336328 |  | 3834.6967 | 99.115214 |
| 3836.5609 | 99.234585 |  | 3836.5609 | 98.30269 |  | 3836.5609 | 98.912772 |
| 3838.4252 | 99.128067 |  | 3838.4252 | 98.312651 |  | 3838.4252 | 98.869707 |
| 3840.2894 | 99.297951 |  | 3840.2894 | 98.413555 |  | 3840.2894 | 99.087382 |
| 3842.1536 | 98.727448 |  | 3842.1536 | 98.073378 |  | 3842.1536 | 97.811678 |
| 3844.0178 | 97.821096 |  | 3844.0178 | 97.492248 |  | 3844.0178 | 96.186997 |
| 3845.882 | 97.98077 |  | 3845.882 | 97.512778 |  | 3845.882 | 96.596949 |
| 3847.7462 | 98.407775 |  | 3847.7462 | 97.664596 |  | 3847.7462 | 97.28621 |
| 3849.6105 | 98.645059 |  | 3849.6105 | 97.762432 |  | 3849.6105 | 97.680123 |
| 3851.4747 | 99.131979 |  | 3851.4747 | 98.145362 |  | 3851.4747 | 98.915531 |
| 3853.3389 | 99.685476 |  | 3853.3389 | 98.562054 |  | 3853.3389 | 100.39128 |
| 3855.2031 | 100.06339 |  | 3855.2031 | 98.89477 |  | 3855.2031 | 101.35727 |
| 3857.0673 | 99.162438 |  | 3857.0673 | 98.452615 |  | 3857.0673 | 98.501142 |
| 3858.9316 | 96.83467 |  | 3858.9316 | 96.939956 |  | 3858.9316 | 94.192226 |
| 3860.7958 | 97.370254 |  | 3860.7958 | 97.054448 |  | 3860.7958 | 95.490308 |
| 3862.66 | 98.55054 |  | 3862.66 | 97.655078 |  | 3862.66 | 97.626944 |
| 3864.5242 | 99.370535 |  | 3864.5242 | 98.144305 |  | 3864.5242 | 99.143207 |
| 3866.3884 | 99.557664 |  | 3866.3884 | 98.315628 |  | 3866.3884 | 99.28747 |
| 3868.2526 | 99.050918 |  | 3868.2526 | 98.103709 |  | 3868.2526 | 98.191213 |
| 3870.1169 | 98.257921 |  | 3870.1169 | 97.674364 |  | 3870.1169 | 97.239257 |
| 3871.9811 | 98.870672 |  | 3871.9811 | 97.994644 |  | 3871.9811 | 98.877226 |
| 3873.8453 | 98.97356 |  | 3873.8453 | 98.017691 |  | 3873.8453 | 98.446666 |
| 3875.7095 | 97.852872 |  | 3875.7095 | 97.392216 |  | 3875.7095 | 96.436345 |
| 3877.5737 | 98.312584 |  | 3877.5737 | 97.656301 |  | 3877.5737 | 97.247445 |
| 3879.438 | 98.411904 |  | 3879.438 | 97.690933 |  | 3879.438 | 97.274664 |
| 3881.3022 | 98.826197 |  | 3881.3022 | 97.907676 |  | 3881.3022 | 98.261705 |
| 3883.1664 | 99.67404 |  | 3883.1664 | 98.414944 |  | 3883.1664 | 99.73421 |
| 3885.0306 | 99.010707 |  | 3885.0306 | 98.120208 |  | 3885.0306 | 98.130642 |
| 3886.8948 | 98.422972 |  | 3886.8948 | 97.780764 |  | 3886.8948 | 97.303923 |
| 3888.759 | 99.046535 |  | 3888.759 | 98.052199 |  | 3888.759 | 98.258835 |
| 3890.6233 | 98.452149 |  | 3890.6233 | 97.642061 |  | 3890.6233 | 97.041658 |
| 3892.4875 | 98.43064 |  | 3892.4875 | 97.618304 |  | 3892.4875 | 97.593325 |
| 3894.3517 | 99.327767 |  | 3894.3517 | 98.14826 |  | 3894.3517 | 99.141003 |
| 3896.2159 | 98.757863 |  | 3896.2159 | 97.893292 |  | 3896.2159 | 97.81367 |
| 3898.0801 | 98.226644 |  | 3898.0801 | 97.661476 |  | 3898.0801 | 97.1859 |
| 3899.9444 | 98.935686 |  | 3899.9444 | 98.09032 |  | 3899.9444 | 98.696099 |
| 3901.8086 | 99.566181 |  | 3901.8086 | 98.476706 |  | 3901.8086 | 99.872197 |
| 3903.6728 | 99.144716 |  | 3903.6728 | 98.233726 |  | 3903.6728 | 99.049111 |
| 3905.537 | 98.347442 |  | 3905.537 | 97.759586 |  | 3905.537 | 97.698769 |
| 3907.4012 | 98.199473 |  | 3907.4012 | 97.579522 |  | 3907.4012 | 96.972346 |
| 3909.2654 | 97.960547 |  | 3909.2654 | 97.268181 |  | 3909.2654 | 96.095922 |
| 3911.1297 | 98.040473 |  | 3911.1297 | 97.2249 |  | 3911.1297 | 96.505664 |
| 3912.9939 | 98.696146 |  | 3912.9939 | 97.615989 |  | 3912.9939 | 97.78482 |
| 3914.8581 | 99.145584 |  | 3914.8581 | 97.964982 |  | 3914.8581 | 98.556888 |
| 3916.7223 | 99.221501 |  | 3916.7223 | 98.104457 |  | 3916.7223 | 98.766215 |
| 3918.5865 | 99.380851 |  | 3918.5865 | 98.211079 |  | 3918.5865 | 99.135621 |
| 3920.4508 | 99.339226 |  | 3920.4508 | 98.133195 |  | 3920.4508 | 98.859628 |
| 3922.315 | 98.74303 |  | 3922.315 | 97.779499 |  | 3922.315 | 97.786564 |
| 3924.1792 | 98.555986 |  | 3924.1792 | 97.67134 |  | 3924.1792 | 97.60735 |
| 3926.0434 | 98.809604 |  | 3926.0434 | 97.823335 |  | 3926.0434 | 98.092476 |
| 3927.9076 | 99.095899 |  | 3927.9076 | 97.987228 |  | 3927.9076 | 98.411564 |
| 3929.7718 | 98.772983 |  | 3929.7718 | 97.807493 |  | 3929.7718 | 97.716302 |
| 3931.6361 | 98.833207 |  | 3931.6361 | 97.839287 |  | 3931.6361 | 97.970557 |
| 3933.5003 | 99.108783 |  | 3933.5003 | 97.94925 |  | 3933.5003 | 98.63524 |
| 3935.3645 | 98.906676 |  | 3935.3645 | 97.8703 |  | 3935.3645 | 98.317079 |
| 3937.2287 | 98.485423 |  | 3937.2287 | 97.650252 |  | 3937.2287 | 97.462417 |
| 3939.0929 | 98.517943 |  | 3939.0929 | 97.637365 |  | 3939.0929 | 97.517173 |
| 3940.9572 | 98.96049 |  | 3940.9572 | 97.87528 |  | 3940.9572 | 98.221068 |
| 3942.8214 | 99.141706 |  | 3942.8214 | 98.014824 |  | 3942.8214 | 98.526598 |
| 3944.6856 | 99.208999 |  | 3944.6856 | 98.091093 |  | 3944.6856 | 98.816395 |
| 3946.5498 | 99.167346 |  | 3946.5498 | 98.071194 |  | 3946.5498 | 98.477543 |
| 3948.414 | 98.843668 |  | 3948.414 | 97.902754 |  | 3948.414 | 97.685768 |
| 3950.2782 | 98.950271 |  | 3950.2782 | 97.918878 |  | 3950.2782 | 98.13922 |
| 3952.1425 | 99.053605 |  | 3952.1425 | 97.924877 |  | 3952.1425 | 98.314457 |
| 3954.0067 | 98.786084 |  | 3954.0067 | 97.8022 |  | 3954.0067 | 97.747493 |
| 3955.8709 | 98.719085 |  | 3955.8709 | 97.748599 |  | 3955.8709 | 97.728209 |
| 3957.7351 | 98.905598 |  | 3957.7351 | 97.810443 |  | 3957.7351 | 98.104366 |
| 3959.5993 | 99.059216 |  | 3959.5993 | 97.927261 |  | 3959.5993 | 98.319823 |
| 3961.4636 | 99.032107 |  | 3961.4636 | 97.954562 |  | 3961.4636 | 98.294397 |
| 3963.3278 | 99.006667 |  | 3963.3278 | 97.93108 |  | 3963.3278 | 98.263004 |
| 3965.192 | 99.038124 |  | 3965.192 | 99.550132 |  | 3965.192 | 98.233932 |
| 3967.0562 | 98.922414 |  | 3967.0562 | 99.555776 |  | 3967.0562 | 98.027318 |
| 3968.9204 | 98.891865 |  | 3968.9204 | 99.561421 |  | 3968.9204 | 98.12025 |
| 3970.7846 | 99.045759 |  | 3970.7846 | 99.567065 |  | 3970.7846 | 98.380686 |
| 3972.6489 | 99.123284 |  | 3972.6489 | 99.572709 |  | 3972.6489 | 98.453762 |
| 3974.5131 | 99.028814 |  | 3974.5131 | 99.578354 |  | 3974.5131 | 98.375795 |
| 3976.3773 | 99.024504 |  | 3976.3773 | 99.583998 |  | 3976.3773 | 98.342923 |
| 3978.2415 | 99.093067 |  | 3978.2415 | 99.589642 |  | 3978.2415 | 98.225991 |
| 3980.1057 | 99.010812 |  | 3980.1057 | 99.595286 |  | 3980.1057 | 98.058719 |
| 3981.97 | 98.959667 |  | 3981.97 | 99.600931 |  | 3981.97 | 98.064606 |
| 3983.8342 | 99.010501 |  | 3983.8342 | 99.606575 |  | 3983.8342 | 98.232306 |
| 3985.6984 | 99.072497 |  | 3985.6984 | 99.612219 |  | 3985.6984 | 98.28705 |
| 3987.5626 | 99.025549 |  | 3987.5626 | 99.617863 |  | 3987.5626 | 98.274551 |
| 3989.4268 | 98.967837 |  | 3989.4268 | 99.623508 |  | 3989.4268 | 98.257917 |
| 3991.291 | 99.036288 |  | 3991.291 | 99.629152 |  | 3991.291 | 98.278 |
| 3993.1553 | 99.070339 |  | 3993.1553 | 99.634796 |  | 3993.1553 | 98.318712 |
| 3995.0195 | 98.948612 |  | 3995.0195 | 99.64044 |  | 3995.0195 | 98.202399 |
| 3996.8837 | 98.902884 |  | 3996.8837 | 99.646085 |  | 3996.8837 | 98.219282 |
| 3998.7479 | 98.992027 |  | 3998.7479 | 99.651729 |  | 3998.7479 | 98.313467 |
| 4000.6121 | 99.016493 |  | 4000.6121 | 99.657373 |  | 4000.6121 | 98.210563 |

**SEM Images of RS, A and D**

**

**

**Supplementary Figure 4: SEM Image of Native RS**

**

**

**Supplementary Figure 5: SEM Image of PWW pretreated RS (A)**

**

**

**Supplementary Figure 6: SEM Image of Distilled water pretreated RS (D)**

**X-ray spectra of Native RS**

**X-ray spectra of PWW pretreated RS (A)**

**X-ray spectra of Distilled water pretreated RS (D)**

**Energy dispersive X-ray spectroscopy (EDX) analysis results**

**Supplementary Figure 7: EDX test report of Native RS**

**Supplementary Figure 8: EDX test report of PWW pretreated RS (A)**

**Supplementary Figure 9: EDX test report of Distilled water pretreated RS (D)**

**Supplementary Table 2: Compositional analysis results of native and pretreated rice straw with standard deviation**

| Components analysed (%) | RS | A | D |
| --- | --- | --- | --- |
| Cellulose | 43.04 ± 2.02 | 53.16 ± 1.84 | 44.79 ± 1.34 |
| Hemicellulose | 28.59 ± 1.7 | 21.36 ± 2.46 | 27.44 ± 1.47 |
| Lignin | 19.06 ± 1.3 | 9.86 ± 1.36 | 18.88 ± 1.05 |
| Extractives | 1.97 ± 0.32 | 1.57 ± 0.22 | 1.84 ± 0.43 |
| Ash content | 11.82 ± 0.65 | 17.67 ± 0.83 | 11.22 ± 0.31 |
| Acid soluble silica | 89.60 ± 3.56 | 55.30 ± 4.12 | 88.98 ± 4.73 |
| Reducing sugar | 3.26 ± 0.34 | 16.83 ± 1.24 | 5.28 ± 0.27 |
| Volatile solids | 88.28 ± 4.5 | 82.33 ± 4.26 | 88.78 ± 0.19 |
| Crystallinity index | 54.55 ± 0.68 | 31.90 ± 0.33 | 52.10 ± 0.51 |
| Moisture content | 4.29 ± 0.14 | 3.99 ± 0.13 | 4.21 ± 0.12 |
| Protein | 4.28 ± 0.20 | 3.02 ± 0.14 | 4.19 ± 0.17 |
| Phosphorus | 0.09 ± 0.02 | 0.25 ± 0.08 | 0.08 ± 0.03 |
| Nitrogen | 0.71 ± 0.10 | 0.52 ± 0.05 | 0.65 ± 0.07 |
| C/N ratio | 45.34 ± 1.12 | 79.98 ± 1.33 | 64.09 ± 1.01 |
| Potassium | 1.61 ± 0.11 | 1.03 ± 0.07 | 1.32 ± 0.16 |
| Iron^*^ | 727.62 ± 0.23 | 501.40 ± 0.25 | 654.21 ± 0.12 |
| Zinc^*^ | 14.96 ± 0.08 | 18.07 ± 0.11 | 14.45 ± 0.07 |
| Sulphur^*^ | 17.81 ± 0.13 | 17.62 ± 0.27 | 17.21 ± 0.09 |

^*^μg/g _Dry weight_
